# CD4 and LAG-3 from sharks to humans: related molecules with motifs for opposing functions

**DOI:** 10.3389/fimmu.2023.1267743

**Published:** 2023-12-21

**Authors:** Fumio Takizawa, Keiichiro Hashimoto, Ryuichiro Miyazawa, Yuko Ohta, Ana Veríssimo, Martin F. Flajnik, David Parra, Kotaro Tokunaga, Hiroaki Suetake, J. Oriol Sunyer, Johannes M. Dijkstra

**Affiliations:** ^1^ Faculty of Marine Science and Technology, Fukui Prefectural University, Obama, Fukui, Japan; ^2^ Emeritus Professor, Center for Medical Science, Fujita Health University, Toyoake, Aichi, Japan; ^3^ Department of Pathobiology, School of Veterinary Medicine, University of Pennsylvania, Philadelphia, PA, United States; ^4^ Department of Microbiology and Immunology, University of Maryland, Baltimore, MD, United States; ^5^ CIBIO‐InBIO, Research Center in Biodiversity and Genetic Resources, University of Porto, Vairão, Portugal; ^6^ BIOPOLIS Program in Genomics, Biodiversity and Land Planning, CIBIO, Vairão, Portugal; ^7^ HIPRA, Aqua Department, Amer, Girona, Spain; ^8^ Aqua World Ibaraki Prefectural Oarai Aquarium, Oarai, Japan; ^9^ Center for Medical Science, Fujita Health University, Toyoake, Aichi, Japan

**Keywords:** CD4, LAG-3, evolution, shark, cytoplasmic tail, activation motif, inhibitory motif, ITIM

## Abstract

CD4 and LAG-3 are related molecules that are receptors for MHC class II molecules. Their major functional differences are situated in their cytoplasmic tails, in which CD4 has an activation motif and LAG-3 an inhibitory motif. Here, we identify shark *LAG-3* and show that a previously identified shark *CD4-like* gene has a genomic location, expression pattern, and motifs similar to *CD4* in other vertebrates. In nurse shark (*Ginglymostoma cirratum*) and cloudy catshark (*Scyliorhinus torazame*), the highest *CD4* expression was consistently found in the thymus whereas such was not the case for *LAG-3*. Throughout jawed vertebrates, the CD4 cytoplasmic tail possesses a Cx(C/H) motif for binding kinase LCK, and the LAG-3 cytoplasmic tail possesses (F/Y)xxL(D/E) including the previously determined FxxL inhibitory motif resembling an immunoreceptor tyrosine-based inhibition motif (ITIM). On the other hand, the acidic end of the mammalian LAG-3 cytoplasmic tail, which is believed to have an inhibitory function as well, was acquired later in evolution. The present study also identified *CD4-1*, *CD4-2*, and *LAG-3* in the primitive ray-finned fishes bichirs, sturgeons, and gars, and experimentally determined these sequences for sterlet sturgeon (*Acipenser ruthenus*). Therefore, with CD4-1 and CD4-2 already known in teleosts (modern ray-finned fish), these two CD4 lineages have now been found within all major clades of ray-finned fish. Although different from each other, the cytoplasmic tails of ray-finned fish CD4-1 and chondrichthyan CD4 not only contain the Cx(C/H) motif but also an additional highly conserved motif which we expect to confer a function. Thus, although restricted to some species and gene copies, in evolution both CD4 and LAG-3 molecules appear to have acquired functional motifs besides their canonical Cx(C/H) and ITIM-like motifs, respectively. The presence of CD4 and LAG-3 molecules with seemingly opposing functions from the level of sharks, the oldest living vertebrates with a human-like adaptive immune system, underlines their importance for the jawed vertebrate immune system. It also emphasizes the general need of the immune system to always find a balance, leading to trade-offs, between activating and inhibiting processes.

## 1 Introduction

Cluster of differentiation 4 (CD4) and lymphocyte activation gene-3 (LAG-3, CD223) are related molecules encoded by duplicated genes that are closely linked in the genome (1). They share the ability to bind major histocompatibility complex (MHC) class II molecules (2, 3) that present peptide antigens at the surface of professional antigen-presenting cells such as B cells, macrophages, and dendritic cells (4). Besides MHC class II, additional ligands have been found for both CD4 (5) and LAG-3 (reviewed in (6)). However, in multiple fish species that independently lost MHC class II genes, the (intact) *CD4* and *LAG-3* genes appear to have been lost as well (7–10), which—at least in fish—argues against CD4 and LAG-3 having important MHC-independent functions. CD4 is an activating co-receptor within T-cell-receptor(TCR)αβ/CD3/CD4 complexes (11) on helper T (Th) cells and subsets of regulatory T (Treg) cells. CD4 lowers the threshold for a stimulatory interaction between the TCR complex and the peptide/MHC complex of an antigen-presenting cell (12–14). Lymphocyte cell-specific protein-tyrosine kinase (LCK), bound to the CxC motif in the CD4 cytoplasmic tail, activates the TCR complex by phosphorylation of immunoreceptor tyrosine-based activation motifs (ITAMs) of CD3 (11, 15, 16).

LAG-3 derives its name from being upregulated in activated natural killer (NK) and T cells (1), and also is constitutively expressed on a subset of Tregs (17). LAG-3 is an inhibitory receptor that limits the activation and proliferation of the LAG-3-expressing cells (18–20) and has been identified as an “immune checkpoint” molecule that can be targeted by antibodies to enhance anti-cancer immune responses (reviewed in (21)). LAG-3 inhibitory function is mediated through its cytoplasmic tail (22, 23), and LAG-3 can even in the absence of MHC class II interfere with TCR/CD3 signaling (24). However, there are still many questions on the mechanism of action of LAG-3, and, for example, how it binds MHC class II is not even known (6).

Both CD4 and LAG-3 are type I transmembrane molecules with four extracellular immunoglobulin-like (Ig-like) domains, named D1-to-D4 starting from the N-terminus. LAG-3 was recently found to be able to form homodimer structures, mediated by D2 domain interaction (25), and, to some extent, CD4 probably functions in a homodimeric form as well (26–28). CD4 and LAG-3 bind MHC class II with their D1 domains, neither with a very high affinity, but LAG-3 more strongly than CD4 (25, 29–32). The absence of high-affinity binding interactions probably helps explain why the sequence conservation of the CD4 and LAG-3 ectodomain sequences is relatively poor. This has led to delays and difficulties in *CD4* and *LAG-3* gene identification across wide species borders (e.g., (33)).

In 2004 (34) and 2006 (33), in teleost (modern bony) fish, two types of CD4 molecules were found that eventually were named CD4-1 and CD4-2 (35). In rainbow trout, we established monoclonal antibodies against both CD4 types and found that they are expressed together or individually on similar cell types as CD4 in mammals, namely subsets of T cells (CD4-1^+^/CD4-2^+^ or CD4-2^+^) and macrophages (CD4-1^+^) (36). Differences in function between CD4-1 and CD4-2 have not been established yet. Teleost fish also possess a *LAG-3* gene (37). However, studies of LAG-3 protein and function have, to the best of our knowledge, only been performed in mammals. In the present study, *CD4-1*, *CD4-2*, and *LAG-3* have been identified for the first time in primitive ray-finned fishes, and for sterlet sturgeon we confirmed the sequences experimentally.

In 2014, Venkatesh et al. (38) stated that in sharks the Th and Treg cell system could not be the same as in mammals, because they believed several pivotal cytokines to be absent and deemed the *CD4/LAG-3*-family (*CD4-like*) gene that they identified unsuitable for representing CD4 function. Their proposed absence of certain cytokines was shown to be flawed and driven by the difficulties of observing gene homologies across broad species borders (39, 40). In the present study, we identified *LAG-3* genes in sharks, and show that the *CD4-like* gene identified by Venkatesh et al. (38) can be characterized as *CD4* based on a number of properties, including gene location, expression pattern, and sequence motifs.

The present study describes CD4 and LAG-3 across jawed vertebrate species and is not only important for researchers with an interest in the evolution of the immune system, but also should help to better understand, now and in the future, the still enigmatic LAG-3 by identifying its conserved features. The most important conserved features that distinguish between CD4 and LAG-3 appear to be situated in their cytoplasmic tails, being a Cx(C/H) motif in CD4 and an ITIM-like motif in LAG-3.

## 2 Materials and methods

### 2.1 Identification and analysis of sequences

*CD4* and *LAG-3* sequences were found and retrieved with the help of similarity searches and gene prediction software (FGENESH (41)) from various datasets at the National Center for Biotechnology Information (NCBI) (https://pubmed.ncbi.nlm.nih.gov, accessed on 1 April 2023). Deduced CD4 and LAG-3 sequences were aligned by hand as described previously, in consideration of intron/exon organization, structural elements, and evolutionary likelihood of events (42). Leader sequences were predicted by SignalP software (https://services.healthtech.dtu.dk/services/SignalP-5.0/ accessed on 1 April 2023). Secondary structures in the cytoplasmic tail were predicted by Jpred4 software (43). Phylogenetic tree analyses were performed with MEGA7 software (44).

### 2.2 cDNA and gDNA sequence analysis of *CD4-1*, *CD4-2*, and *LAG-3* in sturgeon

Sterlet sturgeon (*Acipenser ruthenus*) was obtained from a local pet shop in Spain and euthanized by a tricaine methanesulfonate (MS-222; Sigma-Aldrich) overdose in the fish facilities at the Universitat Autónoma de in Barcelona. Spleen, gill, and gut were collected and kept in RNAlater (Sigma-Aldrich) for RNA and DNA extraction. The total RNA of these tissues was purified with Rneasy plus mini kit (Qiagen) and reverse-transcribed into cDNA using The High Capacity cDNA Reverse Transcription Kit (Applied Biosystems) following the manufacturer’s instructions. Genomic DNA from sturgeon spleen was extracted with Dneasy Blood & Tissue Kit (Qiagen). PCR reactions for ORF cloning of sterlet sturgeon *CD4-1*, *CD4-2*, and *LAG-3* were performed with Phusion Hot Start High-Fidelity DNA Polymerase (New England BioLabs), synthesized cDNA, and gene-specific primers according to the manufacturer’s guidebook. Likewise, gDNA was amplified with Phusion Hot Start High-Fidelity DNA Polymerase, sturgeon gDNA, and gene-specific primers to confirm exon/intron boundaries in immunoglobulin domain coding sequences of *CD4-1*, *CD4-2*, and *LAG-3*. PCR conditions were 98 °C for 30 s, followed by 35 cycles of 98 °C for 10 s, 60 °C for 5 s, and 72 °C for 30 s. The sequences of primers for cDNA and gDNA cloning, and the cloning strategies, are explained in **Supplementary File 1**. PCR products were cloned into vector pGEM-T Easy (Promega) and sequenced using BigDye Terminator v3.1 Cycle Sequencing Kit (Applied Biosystems) and an Applied Bio-systems 3130 Genetic Analyzer (Applied Biosystems). The cDNA sequences of sterlet sturgeon *CD4-1*, *CD4-2*, and *LAG-3* are deposited in GenBank under the accession numbers LC745920, LC745921, and LC745922, respectively, and the genomic sequences as LC746166-to-LC746172.

### 2.3 Sequencing and expression analysis of *CD4* and *LAG-3* in cloudy catshark

Cloudy catshark (*Scyliorhinus torazame*) individuals were obtained and sampled in Ibaraki Prefectural Oarai Aquarium. After euthanasia of fish with an overdose of MS-222 (500 mg/L; Sigma) followed by decapitation, brain, gill, thymus, spleen, liver, pancreas, spiral intestine, and muscle were collected in RNAiso plus (Takara Bio) with 5 mm zirconium beads and were homogenized using Tissue Lyser (Qiagen). After addition of chloroform to the homogenized samples, the total RNA in an upper aqueous phase was purified by ISOSPIN Cell & Tissue RNA (Nippon Gene) with DNAse I treatment according to the manufacturer’s manual. Genomic DNA from spleen was extracted with DNeasy Blood & Tissue Kit (Qiagen). For primer sequences and a detailed explanation of the amplification strategies, see **Supplementary File 1**. All animal procedures were approved by the Institutional Animal Care and Use Committees of the Fukui Prefectural University (2023-F4-1).

For determining sequences, total RNA from gill tissue was reverse-transcribed into cDNA for 5’- and 3’-RACE PCR with SMARTer RACE 5’/3’ Kit (Takara Bio) for full-length cDNA cloning of catshark *CD4* and *LAG-3* genes. PCR reactions for RACE and ORF cloning were performed with KOD One PCR Master Mix -Blue- (Toyobo) according to the manufacturer’s guidebook. PCR conditions were 98 °C for 30 s, followed by 35 cycles of 98 °C for 10 s, 60 °C for 5 s, and 68 °C for 30 s. PCR products were cloned into pCR2.1 Vector with TA Cloning Kit (Invitrogen), and sequenced using BigDye Terminator v3.1 Cycle Sequencing Kit (Applied Biosystems) and a 3500 Genetic Analyzer (Applied Biosystems). The cDNA sequences of catshark *CD4* and *LAG-3* are deposited in GenBank under the accession numbers LC770928 and LC770929, respectively.

For expression analysis, the concentrations of total RNA samples from the aforementioned tissues from four catshark individuals were measured with The NanoDrop 1000 Spectrophotometer (Thermo Fischer). Two μg of total RNA from each sample (with one exception) was reverse-transcribed into cDNA in a 20 μl total reaction volume using The High Capacity cDNA Reverse Transcription Kit (Applied Biosystems) following the manufacturer’s instructions. Because no more RNA could be obtained, for the thymus from one individual only 1.2 μg of total RNA was used for reverse transcription. The synthesized cDNAs were diluted with 80 μl of nuclease-free water. For real-time PCR, reaction mixture containing 2 μl of cDNA, primer set specific to target genes, and KOD SYBR qPCR Mix (Toyobo) was prepared in 8 μl total reaction volume and amplified in 3-step cycling (two minutes at 98°C for pre-denaturation, and 40 cycles of 98°C for 10 s, 60°C for 10 s, and 68°C for 30 s) followed by analysis by melt curve step in CFX96 Touch Real-Time PCR System and CFX Maestro software (Bio-rad). All samples were run in duplicate wells, and samples containing nuclease-free water instead of cDNA were used as no-template control. Specificities of primer sets for their target genes were confirmed by size estimation using agarose gel electrophoresis and by the DNA melting curve of the PCR products. Target gene quantities were determined using a relative standard curve method with total RNA from spleen. A normalized amount of target gene was calculated by dividing the amount of target gene by the amount of *EF-1A*, *RPL13*, or *ACTB* gene as the endogenous housekeeping control, or by the amount of total RNA. Statistical significance was analyzed by one-way ANOVA (Prism 9; GraphPad).

### 2.4 Expression analysis of *CD4*, *LAG-3*, and other immune genes in nurse shark thymus and spleen transcriptomes

RNA-seq datasets from spleen (GenBank SRR652972) and thymus (GenBank SRR652971) of nurse shark (*Ginglymostoma cirratum*) were retrieved from the Bioproject “*Ginglymostoma cirratum Transcriptome or Gene expression*” (GenBank PRJNA183979) (38). Raw RNA-Seq data underwent quality assessment using FastQC (version: 0.11.9) (45) to exclude adapter sequences and low-quality reads. The nurse shark transcriptome was *de novo* assembled from treated files using Trinity with trimmomatic (version: 2.15.1) (46) to generate a *de novo* contig file for RNA read mapping. The assembled contig files were analyzed for CDS by Transcoder (version: 5.7.1) (https://github.com/TransDecoder/TransDecoder) with all chondrichthyans protein databases obtained from Refseq and extracted the CDS region in fasta format. The CDS regions were annotated by BLAST2GO (version: 6.0.3) (47) with megablast using specific nurse shark sequences (**Supplementary File 2**) to utilize for the gene expression analysis. FastQC treated FASTQ files of thymus and spleen were processed as follows: First, adaptor sequences and low-quality reads were trimmed out with Trim Galore (version: 0.6.5) (https://www.bioinformatics.babraham.ac.uk/projects/trim_galore/). Next, processed RNA reads were Quasi-Mapping to the CDS regions of contig file using kallisto (version: 0.46.3) (48) to align the reads. The RNA expression levels of each gene were quantified using kallisto. RNA read counts were normalized to TPM (Transcript Per Million).

## 3 Results and discussion

### 3.1 An overview of CD4 and LAG-3 evolution

**Figure 1:**
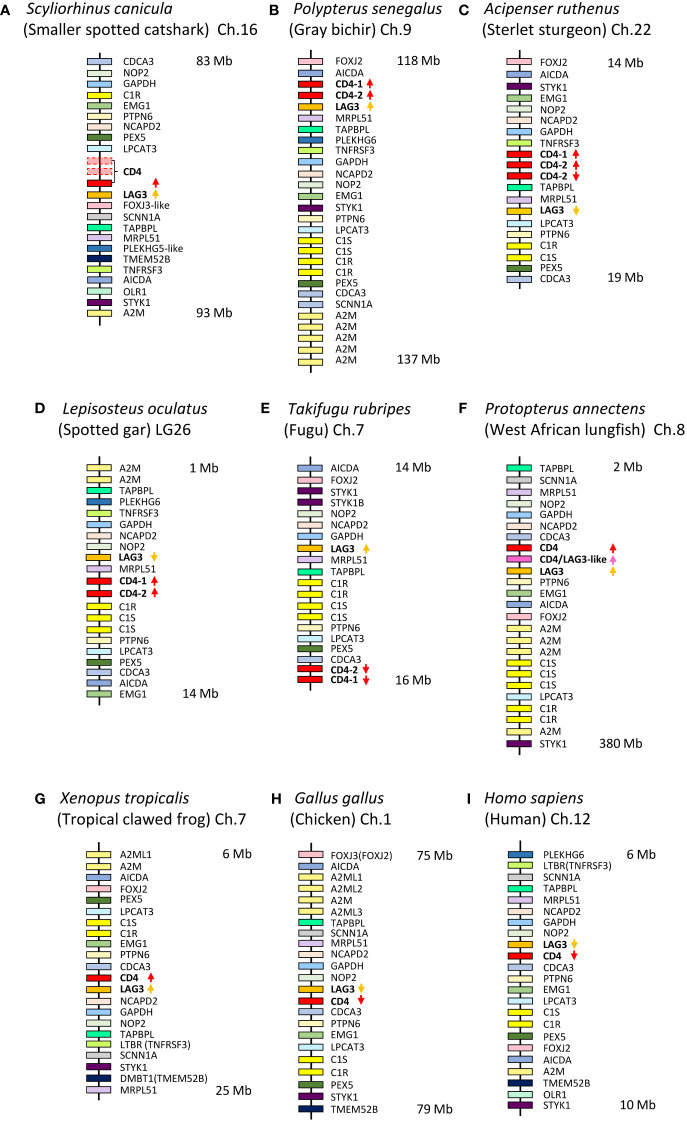
*CD4* and *LAG-3* are found in similar genomic regions from shark to human. Genomic regions of representative species are schematically depicted for **(A)** *Scyliorhinus canicula* (small-spotted catshark), based on information from GenBank assembly accession GCA_902713615.1; **(B)** *Polypterus senegalus* (gray bichir), GCA_016835505.1; **(C)** *Acipenser ruthenus* (Sterlet sturgeon), GCA_010645085.1; **(D)** *Lepisosteus oculatus* (spotted gar), GCA_000242695.1; **(E)** *Takifugu rubripes* (fugu), GCA_901000725.2; **(F)** *Protopterus annectens* (West African lungfish) (for a better understanding of the CD4/LAG-3 hybrid sequence, see paragraph 3.5 and **Supplementary File 10**), GCA_019279795.1; **(G)** *Xenopus tropicalis* (Tropical clawed frog), GCA_000004195.4; **(H)** *Gallus gallus* (chicken), GCA_016699485.1; **(I)** *Homo sapiens* (human), GCA_000001405.29. Only selected genes are shown for highlighting the similarities between the regions. In *S. canicula*, in the direct vicinity of the intact *CD4* gene, there are also fragments of two incomplete *CD4* genes, the largest parts of which are downstream of the intact *CD4* gene and in reverse orientation. The nomenclature of the genes is essentially based on the gene names and the description of the genes in each assembly. Variability between genes with the same names is not shown. The names for *CD4* and *LAG-3* genes are consistent with the analyses of the present study. Depicted distances between genes are not proportional to the biological situation, and the approximate starting and ending points in each chromosome are indicated at the right.

**Figure 2:**
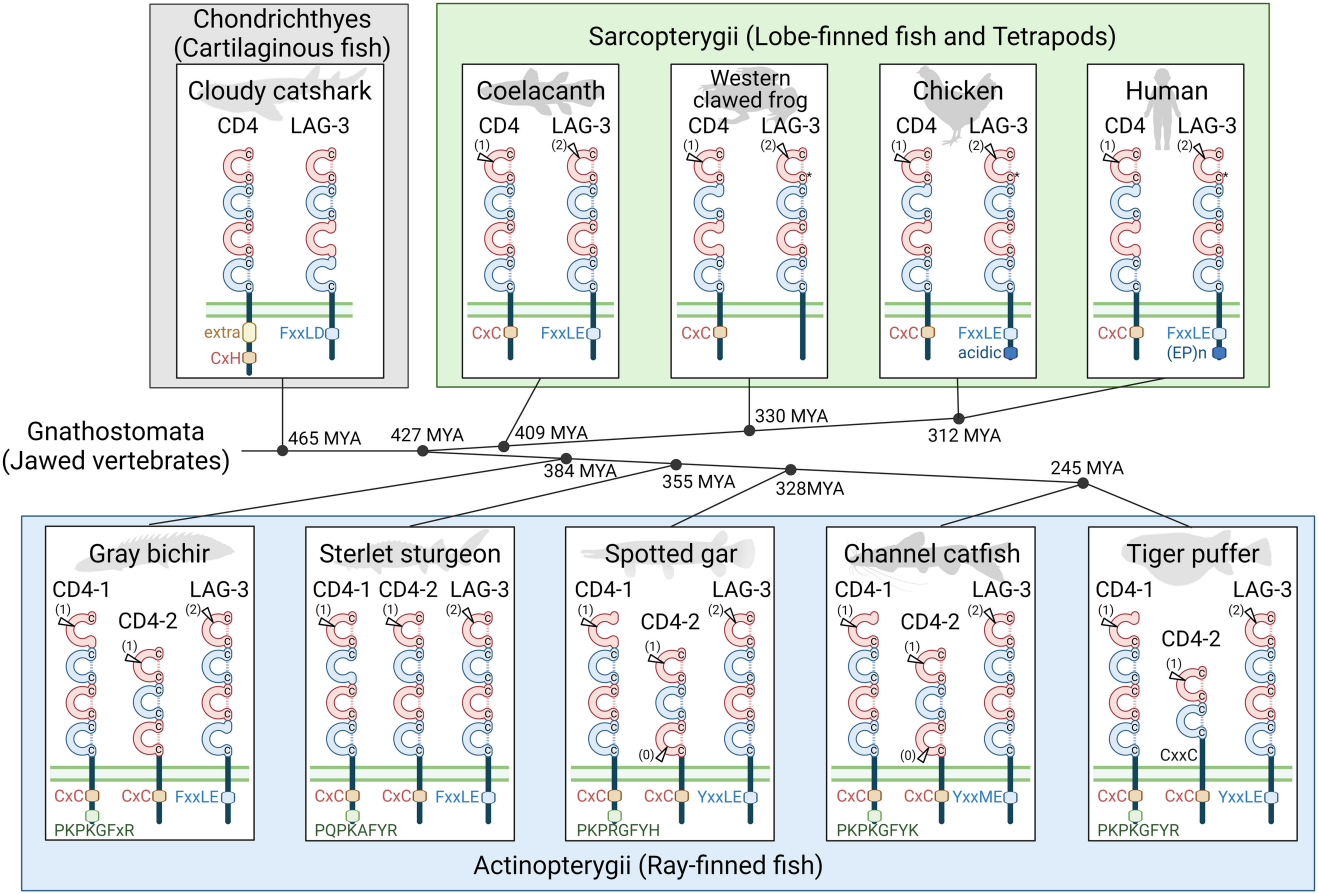
An overview of CD4 and LAG-3 molecular evolution. Genes for CD4 and LAG-3 molecules are found in all major clades of jawed vertebrate species, and the deduced encoded molecules in representative species are schematically shown. These are transmembrane molecules with, in most cases, characteristic functional motifs in their cytoplasmic tails, and between two and four extracellular Ig-like domains; “extra” in the cloudy catshark CD4 cytoplasmic tail refers to an extra exon and an extra conserved motif. The Ig-like domains include V-type category (red) and C2-type category (blue) domains, and in most but not all cases have two cysteines that are typical for Ig-like domains in their β-strands B (upper C) and F (lower C); *C, in LAG-3 in tetrapod species, the cysteine in β-strand F was lost, but the β-strand B cysteine makes a disulfide bridge with a newly acquired cysteine in β-strand G (25). Positions corresponding to introns within Ig-like domain coding sequences are indicated by arrowhead and the intron phase between brackets as (0), (1), or (2). Tiger puffer (fugu; *Takifugu rubripes*) CD4-2 has only two Ig-like domains but, like CD4-2 in many other neoteleost fishes, a hinge region encoded by a separate exon that includes a CxxC motif that we speculate may be involved in homodimerization. For detailed sequence information see **Supplementary File 2** and **Figure 3**. In the middle of the figure, a phylogenetic cladogram is shown with estimations of when, in millions of years ago (MYA), the selected species shared their last common ancestor (based on (49, 50)). Animal figures were created with help of BioRender.

**Figure 3:**
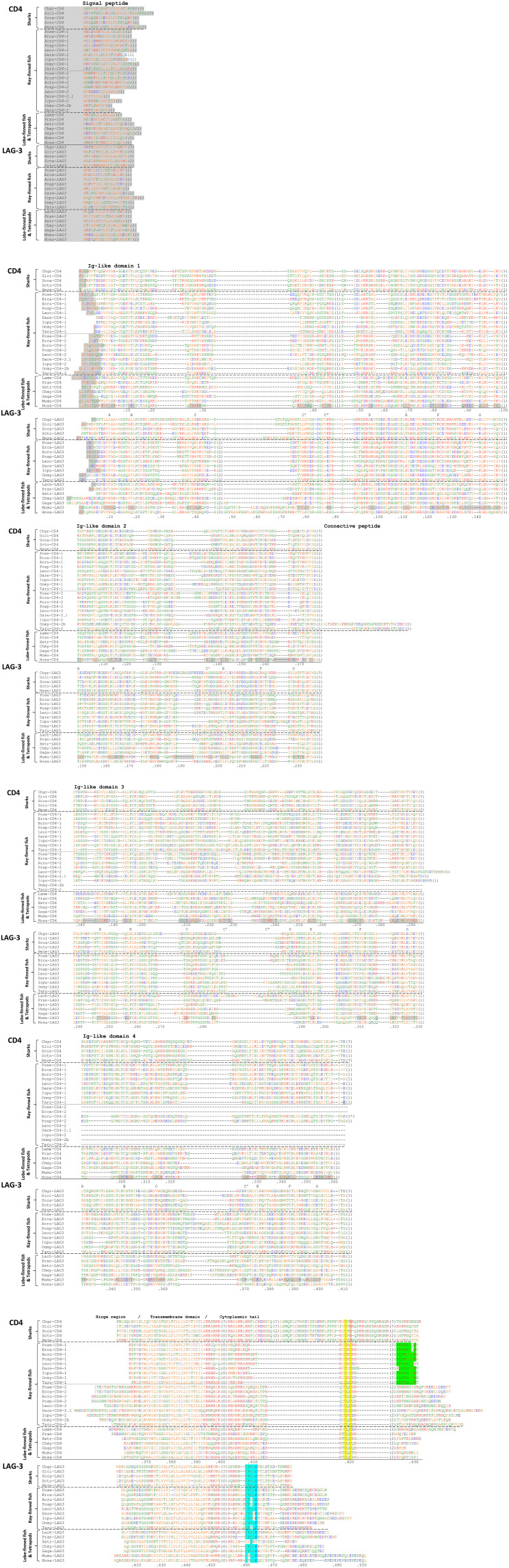
Alignment of the deduced amino acid sequences of CD4 and LAG-3 molecules in representative species. Predicted signal peptides are in Italic font and shaded in gray. Residue numberings under the alignment follow human CD4 or human LAG-3 mature proteins. Gray shading within the ectodomain (and not in Italic font) highlights β-strands as indicated for human CD4 and mouse LAG-3 in PDB accessions 1WIP (27) and 8DGG, respectively, and names for β-strand regions are indicated with the letters A-to-G. The numbers between brackets refer to introns and to their phases at the indicated position (0) or in the preceding codon (1,2); intron positions that can only be speculated because genomic DNA sequence information is not available for that species are indicated with “(?)”. Cysteines are in purple and, based on Hopp and Woods, 1981 (51), red font is used for basic residues, blue for acidic residues, and of the other residues (green and orange) the more hydrophilic ones are in green. Highlighted in the cytoplasmic tail are the motifs Cx(C/H) in CD4 (yellow/orange), P(K/Q)P(K/R)(A/G)FY(H/K/R) in CD4-1, and FPAL(D/E) in LAG-3 (cyan). The species for which sequences are shown are Chgr: *Chiloscyllium griseum* (gray bambooshark); Gici: *Ginglymostoma cirratum* (nurse shark); Scca: *Scyliorhinus canicula* (small-spotted catshark); Scto: *Scyliorhinus torazame* (cloudy catshark); Heze: *Heterodontus zebra* (zebra bullhead shark); Pose: *Polypterus senegalus* (gray bichir); Erca: *Erpetoichthys calabaricus* (Reedfish); Acru: *Acipenser ruthenus* (sterlet sturgeon); Posp: *Polyodon spathula* (Mississippi paddlefish); Leoc: *Lepisosteus oculatus* (spotted gar); Dare: *Danio rerio* (zebrafish); Icpu: *Ictalurus punctatus* (channel catfish); Onmy: *Oncorhynchus mykiss* (rainbow trout); Taru: *Takifugu rubripes* (fugu); Lame: *Latimeria menadoensis* (Menado coelacanth); Lach: *Latimeria chalumnae* (West Indian Ocean Coelacanth); Pran: *Protopterus annectens* (West African lungfish); Xetr: *Xenopus tropicalis* (tropical clawed frog); Chmy: *Chelonia mydas* (green sea turtle); Gaga: *Gallus gallus* (chicken); Mumu: *Mus musculus* (mouse); Hosa: *Homo sapiens* (human). The *Scyliorhinus torazame* (cloudy catshark) CD4 intron positions were determined by comparison of the experimentally determined cDNA sequence with the rather short (in most cases containing only one exon) genomic sequences available as GenBank accessions BFAA01030515, BFAA01044711, BFAA01027384, BFAA01122682, BFAA01153775, BFAA01050327, BFAA01052865, and BFAA01028059; for LAG-3 in this species this was done similarly using GenBank accessions BFAA01031787, BFAA01335495, BFAA01051587, BFAA01228759, and BFAA01053433.

From representative species, using previous publications but also database similarity searches and gene amplification/sequencing experiments, we collected a set of CD4 and LAG-3 sequences listed in **Supplementary File 3**. The genomic locations of their genes in representative species are shown in **Figure 1**, their molecular features in representative species are schematically shown in **Figure 2**, and their full-length amino acid sequences are aligned in **Figure 3**. Both CD4 and LAG-3 sequences could be found in all three major clades of jawed vertebrate species, namely Chondrichthyes (sharks, rays, and chimaeras), Actinopterygii (ray-finned fish), and Sarcopterygii (lobe-finned fish and tetrapods). A CD4/LAG-3 family identity of all these molecules is readily concluded based on overall sequence similarities, as for example shown by the top matches found upon database similarity searches (for shark sequences see **Supplementary File 4**) as is reflected in the names given for the shark sequences by automatic annotation programs (e.g., see GenBank accessions XP_038629972 or XP_038678308). However, apart from preserving some shared CD4/LAG-3 family characteristic motifs and organization (see below), the ectodomain sequences have so far diverged that in widely diverged species they, arguably, do not allow firm conclusions on CD4 or LAG-3 identity beyond the CD4/LAG-3 family level, and in some computerized whole-sequence-based phylogenetic tree analyses there is no clear separation into CD4 and LAG-3 branches (for examples see (37, 38)). However, by using Maximum Likelihood phylogenetic tree analysis for all or a subset of the full-length sequences compared in **Figure 3**, we can find clear LAG-3 or CD4 clusters, respectively (**Supplementary File 5**). Moreover, if the full-length sequences of the various CD4 and LAG-3 molecules analyzed in the present study are blasted against the here-identified shark CD4 and LAG-3 sequences, they generally but not always find an orthologous (CD4 for CD4, LAG-3 for LAG-3) shark sequence as their top-match (**Supplementary File 6**). Overall, we deem the phylogenetic tree analyses and other whole-length sequence similarity comparisons to be only somewhat/subtly supportive of our CD4 versus LAG-3 assignments, which is quite common when analyzing different but related molecules across such large evolutionary distances. However, as explained below, the combination of gene location, cytoplasmic tail exon organization, amino acid sequence motif distribution, and expression patterns, provide clear distinctions between CD4 and LAG-3.

Shown here for the first time for Chondrichthyes and primitive ray-finned fish, the *CD4*/*LAG-3* family genes have remained within the same genomic region (**Figure 1**). The location of *CD4* in a tandem orientation downstream of *LAG-3* appears to be the ancestral gene organization that is conserved in sharks (**Figure 1A**), bichirs (the most primitive ray-finned fish) (**Figure 1B**), and tetrapod species (**Figures 1G–I**). In contrast, in more modern clades of ray-finned fish, this head-to-tail organization of *LAG-3* and *CD4* has not been conserved (**Figures 1C–E**) (37).

There are two sequence features that can make sharp distinctions between CD4 and LAG-3, one throughout jawed vertebrates and the other one only in Osteichthyes (“bony animals”; Actinopterygii plus Sarcopterygii): (1) Throughout jawed vertebrates, behind the region encoded by the transmembrane region coding exon, the remaining of the cytoplasmic tail of CD4 is encoded by multiple exons and has a Cx(C/H) motif (presumably) involved in cell activation, whereas the remaining of the cytoplasmic tail of LAG-3 is encoded by a single exon and has, with the exception of anurans (frogs), an (F/Y)xxL(D/E) inactivation motif (**Figures 2**, **3**); (2) Throughout Osteichthyes, the D1 domain coding sequence of *CD4* genes includes an internal phase 1 intron while that of *LAG-3* includes an internal phase 2 intron at a different position (**Figures 2**, **3**).

The default situation among jawed vertebrates seems to be the presence of one *CD4* gene and one *LAG-3* gene per haploid genome. However, there are exceptions, most notably the possession of both *CD4-1* and *CD4-2* genes in ray-finned fish.

The CD4 and LAG-3 ectodomains usually consist of four Ig-like domains in V-C2-V-C2 order (“V” and “C2” denote Variable and Constant type 2 categories of the immunoglobulin superfamily, respectively), but in many (though not all) ray-finned fishes the CD4-2 ectodomains show derived organizations and only contain two or three Ig-like domains. It has been postulated, and we agree with the likelihood of this model, that in evolution the V-C2-V-C2 ectodomain organization of the CD4/LAG-3 family molecules originated from the tandem duplication of a V-C2 coding set of exons (1, 3, 52, 53). The sequence motif most supportive for this duplication model is the very unusual WxC motif in β-strand F of both the D2 and D4 domains (1) (**Figure 3**).

A “CD4-like” molecule in lamprey with a V-C2 ectodomain and a WxC motif in the C2 domain has been proposed as representing an ancestral CD4/LAG-3 form (54), but more evidence is probably necessary before accepting a close relationship between this molecule and the CD4/LAG-3 family. Rainbow trout CD4-2, which only has one set of V-C2 domains, has also been proposed to represent the ancestral CD4/LAG-3 molecule (37), but that model fails to account for the CD4-typical intron in the fish CD4-2 D1 coding sequence and the fact that CD4-2 molecules in some other fishes have V-C2-V or even V-C2-V-C2 ectodomains (**Figures 2**, **3**), although the latter was not known at the time the model was postulated (37).

Some of the above is discussed in more detail in the below paragraphs.

### 3.2 CD4 and LAG-3 sequences in sharks

In nurse shark, Venkatesh et al., 2014 (38), found a transcript (GenBank KC707916) that they recognized as belonging to the *CD4*/*LAG-3* family based on sequence similarity and phylogenetic tree analysis, and which we here more specifically identify as *CD4*. The Venkatesh et al. (38) study suggested that KC707916 did not represent a bona fide *CD4* sequence because of the following reasons, here listed together with our counterarguments: (i) The KC707916 sequence *CD4*/*LAG-3* open reading frame (ORF) has two potential start codons. The first one (cagATGt) does not agree with Kozak rules for efficient translation (55) but seems to have been assumed as the protein starting point by Venkatesh et al. (38), despite adding an unusual N-terminal sequence. However, the second potential start codon (cccATGg) has a favorable context for translation (55) and encodes a typical CD4/LAG-3 family N-terminus with a leader sequence (**Figure 3**); (ii) Most CD4 molecules use a CxC motif in the cytoplasmic tail for binding LCK through a tetrahedral clasping structure with four cysteines (the other cysteines are from a CxxC motif in LCK, conserved throughout jawed vertebrates) that together bind a zinc ion (16), whereas KC707916 encodes a CxH motif instead. However, histidine is known to be able to replace cysteine in tetrahedral zinc-binding structures (56) and we showed that the trout CD8α cytoplasmic tail sequence, carrying a CxH motif rather than a CxC motif, binds LCK in a zinc-dependent manner (57). Not reported to this extent before (but see references 38 and 57), CxH instead of CxC appears to be the ancient motif in CD8 as it is found in both CD8α and CD8β of primitive gnathostomes, and only in a shared ancestor of humans with lungfishes the CD8α tail CxH was replaced by CxC while the CD8β tail simply lost the CxH motif; the functional similarity between these two motifs is also suggested by CD8α in salamanders having changed the CxC motif back again into a CxH motif (**Supplementary File 7**); (iii) Venkatesh et al. (38) found for the KC707916 sequence that “it appears to be expressed at higher levels in peripheral lymphoid tissues (RT-PCR data not shown).” However, when we perform blast similarity searches with the CD4 coding sequence of KC707916 against the transcriptomes established by Venkatesh et al. (38) for nurse shark spleen and thymus, the number of transcripts per million is much higher in thymus than in spleen (see below), consistent with *CD4* expression in other species (33, 58–60). As described in the paragraph below, a higher expression of *CD4* in thymus than in spleen was also found by northern blot analysis for nurse shark and RT-PCR experiments for cloudy catshark.

For a few shark species, we could identify both full-length *CD4* and *LAG-3* ORF sequences (**Supplementary File 3**, **Figure 3**). The deduced CD4 and LAG-3 aa sequences of gray bambooshark (*Chiloscyllium griseum*), nurse shark (*Ginglymostoma cirratum*), and zebra bullhead shark (*Heterodontus zebra*) mostly derived from TSA reports are aligned in **Figure 3**, together with sequences deduced from genomic sequence reports for small-spotted catshark (*Scyliorhinus canicula*) and our experimentally determined cDNA sequences for cloudy catshark (*Scyliorhinus torazame*). In sharks, only for *S. canicula* the reported genomic sequences with *CD4* and/or *LAG-3* were long enough to allow comparison of the local gene environments with other species. The reported assembly of the *CD4*/*LAG-3* genomic region in *S. canicula* includes only one intact *CD4* gene which is downstream of, and head-to-tail with, *LAG-3*, as in bichirs and tetrapods (**Figure 1**). Around that intact *CD4* gene are also what seem to be fragments of two *CD4* pseudogenes with high sequence similarity to the intact gene (not shown), and of which the largest (but not all) parts are located downstream and in reverse orientation of the intact *CD4* gene (**Figure 1A**, details not shown). “Whole genome” sequences have been published for several other shark species as well, but many of the reported sequence scaffolds are short or have quality problems so that they do not contain entire genes and/or neighboring genes.

In sharks, neither the *CD4* nor the *LAG-3* genes have an intron within the D1 coding sequence. This can be concluded from *S. canicula* genomic sequences but also from shorter sequence reports for several shark species for *CD4* (whitespotted bambooshark [*Chiloscyllium plagiosum*], GenBank QPFF01463545; whale shark [*Rhincodon typus*], QPMN01042033; cloudy catshark, BFAA01044711; great white shark [*Carcharodon carcharias*], JAGDEE010000362), and *LAG-3* (brownbanded bambooshark [*Chiloscyllium punctatum*], BEZZ01252331; whale shark, QPMN01083740; zebra shark [*Stegostoma fasciatum*], JAFIRC010001213; great white shark, JAGDEE010000433) (summarized in **Figure 3** and data not shown).

Compared to CD4 in other species, the cytoplasmic tail in shark CD4 is considerably longer and encoded by an extra exon, although the other exon borders and their phases in the cytoplasmic tail coding region are as expected (**Figure 3**). This shark-specific intron-exon organization can be concluded from the *S. canicula* genomic sequence report but also agrees with shorter sequence reports for several other shark species (whitespotted bambooshark, GenBank QPFF01523626; whale shark, QPMN01068357; Puget Sound dogfish [*Squalus suckleyi*], JAOAMX010045787; great white shark, QUOW01006312; cloudy catshark, BFAA01153775, BFAA01050327, BFAA01052865, and BFAA01028059) (**Figure 3** and data not shown). The cytoplasmic tails of shark CD4 molecules carry a CxH motif instead of CxC (**Figure 3**).

In contrast to CD4, the shark LAG-3 cytoplasmic tails are encoded from a single exon as found for LAG-3 in other species (**Figure 3**). Furthermore, like LAG-3 in other species, the shark LAG-3 sequences carry an (F/Y)xxL(D/E) motif and also the proline and alanine at positions 2 and 3 within this motif in shark LAG-3 are common in LAG-3 of other species (**Figure 3** cyan shading).

The identification of the shark CD4/LAG-3 family molecules as either CD4 or LAG-3 relies on their genomic location and—most importantly—their cytoplasmic tails. Apart from this, shark CD4 and other CD4 sequences seem to also have a few more subtle similarities that may set them apart from LAG-3 at the following positions (residue numbering as in human CD4 in **Figure 3**): (i) a better conservation of (the molecule length in the region that may form) β-strands C’’ and D in the D3 domain (see below); (ii) a preference for a proline at position 133; (iii) no preference for an alanine or glycine at position 188; (iv) no preference for a glycine at position 311; (v) a slightly longer hydrophobic transmembrane region (the region around position 380 in **Figure 3** [in this figure, the hydrophobic residues are colored orange]).

We did not intensively investigate chimaeras, the sister group of sharks/rays, but point out that Venkatesh et al. (38) already reported a sequence fragment in the chimaera elephant shark that appears to represent a CD4 fragment with a CxH motif containing cytoplasmic tail that is homologous to the shark CD4 sequences presented in the present study, additionally underlining the expected functionality of this CxH motif, and such fragment can also be found for the chimaera small-eyed rabbitfish (*Hydrolagus affinis*; GenBank JAAILG010093779). **Supplementary File 8** shows an alignment of these chimaeran CD4 cytoplasmic tail sequences and those in widely divergent elasmobranchs, showing that among CD4 molecules of Chondrichthyes besides the CxH motif a relatively large set of other residues are highly conserved as well, including a tyrosine, two cysteines, prolines, and others. This is somewhat reminiscent of how the cytoplasmic tails between chondrichthyan CD8α also show a better overall conservation than those in higher level vertebrates that carry a CxC motif (**Supplementary File 7**); possibly, but that is only speculation at this stage, the binding of LCK through a CxC motif is stronger than through a CxH motif, reducing the necessity for other binding factors to be optimal. We assume that the cytoplasmic tail of chondrichthyan CD4, besides binding LCK through a CxH/CxxC Zinc clasp, also participates in novel interactions with LCK and/or other cytosolic signaling molecules.

### 3.3 *CD4* and *LAG-3* expression in cloudy catshark and nurse shark

CD4 molecules are T cell lineage markers and are expressed by a majority of T cells in the thymus, namely by both CD4/CD8 double-positive immature T cells and CD4 single-positive mature T cells. This, combined with the high density of T cells in the thymus, causes this organ to have the highest *CD4* expression as reported for mammals, birds, and teleost fish (both *CD4-1* and *CD4-2* in the case of teleost fish) (33, 58–60). On the other hand, as an activation marker, the expression of *LAG-3* is not consistently the highest in the thymus, and, compared to *CD4*, a more variable/diffuse distribution over immune tissues has been described for *LAG-3* in mammals (61–63) as well as teleost fish (37, 64, 65).

**Figure 4:**
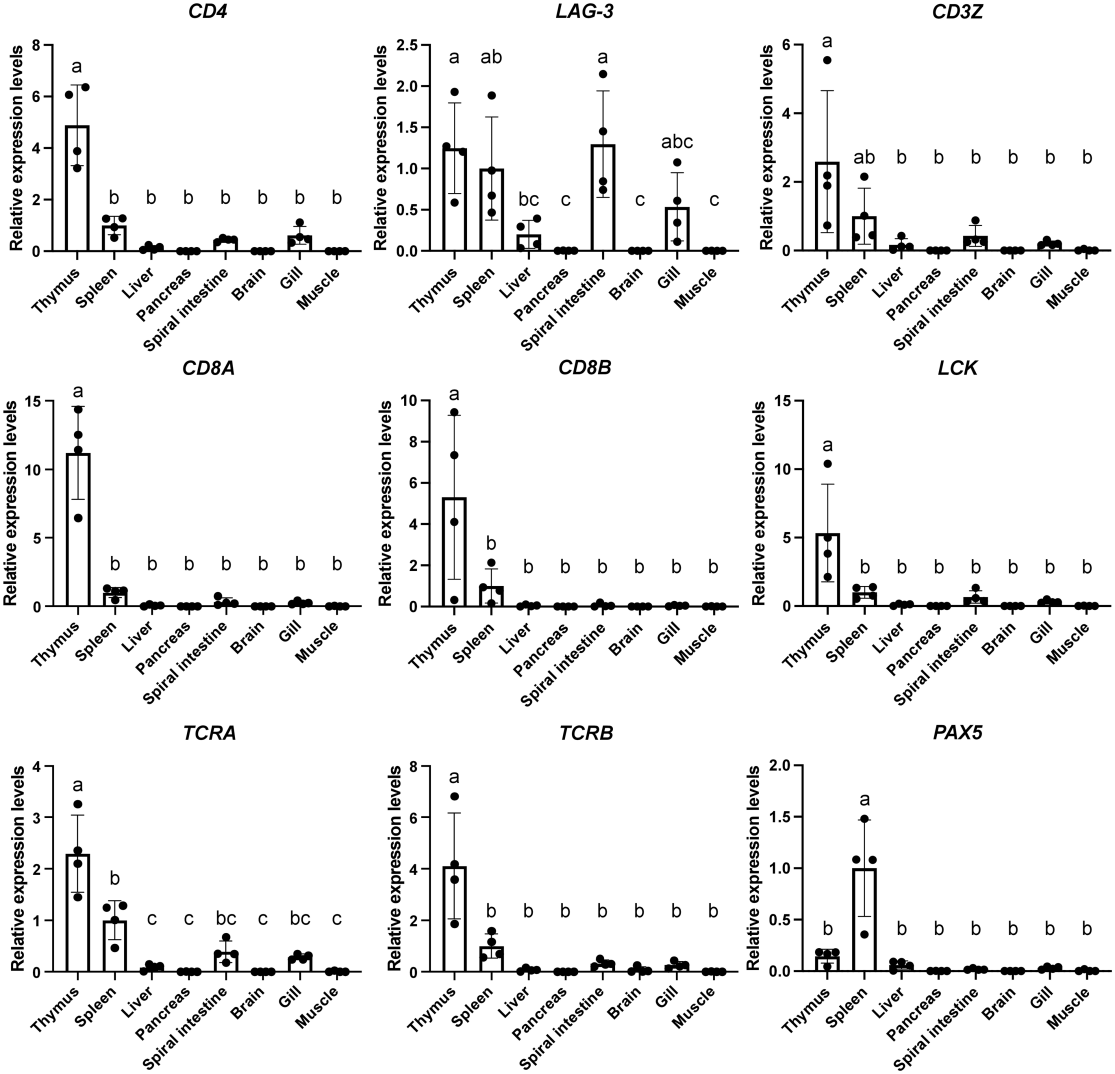
Tissue distribution analyses of *CD4* and *LAG-3* transcripts in cloudy catshark reveal that only *CD4* expression is especially high in the thymus. Transcript levels of catshark *CD4*, *LAG-3*, *CD3 zeta* (*CD3Z)*, *LCK*, *CD8A*, *CD8B*, *T cell receptor alpha* (*TCRA*), *T cell receptor beta* (*TCRB*), and *paired box 5* (PAX5) in indicated tissues were analyzed by real-time PCR. A normalized amount of target gene was calculated by dividing the amount of target gene by the amount of *elongation factor 1-alpha* (*EF-1A*), and transcript expression levels of indicated genes were further normalized to those in spleen (set as 1), which are indicated as mean value ± S.D. with individual values (n = 4 fish). One-way ANOVA with Tukey’s *post-hoc* test was used to assess statistically significant differences between the means of respective tissues, which were shown in different letters (P<0.05).

To assess the expression patterns of *CD4* and *LAG-3* transcripts in sharks, we examined the gene expression analysis of *CD4* and *LAG-3* across multiple tissues in cloudy catshark together with other immune genes using real-time PCR (**Figure 4**; see **Supplementary File 9A** for calibration with other housekeeping genes or total RNA). As known in other species, cloudy catshark *CD4* transcripts were significantly more abundant in the thymus than in other tissues (**Figure 4**, upper left). The expression levels of the T-cell related genes *CD3Z*, *LCK*, *CD8A*, *CD8B*, *TCRA*, and *TCRB* were also highest in thymus, while the B-cell marker *PAX5* was highly expressed in spleen (**Figure 4**). These finding are consistent with previous studies in cartilaginous fish that found genes for molecules of the TCR-CD3 complex to be most abundantly expressed in the thymus (66–68) and suggests that shark CD4 is a T cell lineage marker as in other jawed vertebrates. Meanwhile, cloudy catshark *LAG-3* transcripts were not consistently more abundant in the thymus than in the spleen (**Figure 4**).

We found, by investigation of nurse shark spleen and thymus transcriptomes, generated by Venkatesh et al. (38), for *CD4* and *LAG-3* in nurse shark a similar expression pattern (**Table 1**) as found by RT-PCR in cloudy catshark (**Figure 4**). In the investigated nurse shark individual, the frequency of *CD4* reads was more than five times higher in the thymus than in the spleen, whereas for *LAG-3* such a pattern was not observed, and the expression of other T cell marker genes was also higher in the thymus (**Table 1**). The higher expression of *CD4* in the thymus than in the spleen was also confirmed for another nurse shark individual by northern blot analysis (**Supplementary File 9B**).

**Table 1:** Transcripts per million (TPM) of *CD4*, *LAG-3*, and other immune genes in transcriptomes of Nurse shark tissues (38).

	Spleen (SRR652972)	Thymus (SRR652971)
**CD4**	1.10	6.01
**LAG-3**	0.43	0.39
**CD8A**	18.49	470.56
**CD8B**	6.73	190.14
**LCK**	18.75	171.45
**CD3Z**	104.18	812.26
**TCRA**	114.51	480.93
**TCRB**	44.46	533.84
**PAX5**	3.61	0.04

Gray shading highlights the tissue with the highest expression. CD8A and CD8B are markers for cytotoxic T cells; LCK, CD3Z, TCRA, and TCRB are expressed by T cells; and PAX5 is expressed by B cells.

Furthermore, in nurse shark, we analyzed *CD4* and *LAG-3* expression in single cells by investigating snRNA-seq data generated from spleen tissue by Matz et al., 2023 (69) (**Supplementary File 9C**). Both *CD4* and *LAG-3* were predominantly found in T cell populations and co-expressed with T-cell marker *CD3Z* (**Supplementary File 9C**). Consistent with the findings at the tissue level (**Table 1**), *CD4* expression was found higher than that of *LAG-3* (**Supplementary File 9C**). Importantly, *CD4* appears not to be co-expressed with *CD8A* at a significant level, which agrees with the situation commonly found in mammalian splenocytes; the proportion of *LAG-3* positive cells that are also *CD8A* positive may be more substantial, but the number of *LAG-3* positive cells is probably too low for drawing solid conclusions on this (**Supplementary File 9C**).

In summary, the *CD4* versus *LAG-3* expression pattern in sharks is consistent with that in other species, suggesting a similar division of functions.

### 3.4 CD4-1, CD4-2, and LAG-3 in ray-finned fish: besides CxC, the CD4-1 cytoplasmic tails tend to carry the motif P(K/Q)P(K/R)(A/G)FY(H/K/R)

Previous reports described CD4-1, CD4-2, and LAG-3 in teleost (modern ray-finned) fish (33, 34, 37). Teleost fish CD4-1 and LAG-3 molecules have four Ig-like domains as in CD4/LAG-3 family consensus, but their CD4-2 molecules usually have only two or three Ig-like domains. Teleost CD4-1 and CD4-2 molecules are often but not in all cases expressed by the same cells, and both have been found associated with Th and Treg cells (36, 70–75), and both can bind LCK (76). In the teleost clades Protacanthopterygii (exemplified in **Figure 3** by rainbow trout) and Neoteleostei (exemplified in **Figure 3** by fugu “Taru-CD4-2”), the CD4-2 molecules only have two Ig-like domains, which are in a V-C2 arrangement, followed by a unique linker region with, in many cases, two conserved cysteines in a CxxC motif (**Figures 2**, **3**) that we speculate may participate in homodimerization by forming intermolecular disulfide bridges. In more basal teleosts such as zebrafish and channel catfish, CD4-2 molecules with three Ig-like domains in a V-C2-V arrangement are found (**Figure 3**). Besides canonical CD4-1 and CD4-2 (CD4-2.1) molecules, zebrafish also has an additional, non-canonical molecule of the CD4-2 lineage (CD4-2.2; not shown in this paper), the sequence of which is very similar to CD4-2.1 and which is predicted to be a secreted molecule with five Ig-like domains (71) of which D4 + D5 appear to be a recent duplication of D1 + D2.

In the present study, we also identified *CD4-1*, *CD4-2*, and *LAG-3* in fish species representative for the primitive non-teleost ray-finned fish clades Polypteriformes (bichirs and reedfish), Acipenseriformes (sturgeons and paddlefishes), and Holostei (gars and bowfin) (**Figures 1**–**3**; **Supplementary File 3**). For sterlet sturgeon (*Acipenser ruthenus*), we confirmed these sequences experimentally, except for a second but very similar *CD4-2* gene that we named *CD4-2f* because the second cysteine in the cytoplasmic tail CxC motif is replaced by a phenylalanine (**Figure 1**; **Supplementary File 3**).

An important overall conclusion is that CD4-1 and CD4-2 are ancient lineages, which appear to have originated at the evolutionary start of ray-finned fishes. The deduced CD4-1 and LAG-3 molecules in these species all contain four Ig-like domains (V-C2-V-C2), and this is also found for CD4-2 in the Acipenseriformes sterlet sturgeon and Mississippi paddlefish (*Polyodon spathula*). However, in both Polypteriformes and Holostei the deduced CD4-2 molecules have only three Ig-like domains, organized as V-C2-V (**Figures 2**, **3**). This concludes that, given the relative phylogenetic positions of these two fish clades and Acipenseriformes (**Figure 2**), Polypteriformes and Holostei lost the CD4-2 D4 domain independently.

In ray-finned fishes, the CD4-1 and CD4-2 cytoplasmic tails both carry the CD4-typical CxC activation motif and the LAG-3 cytoplasmic tails carry the LAG-3 typical (F/Y)xxL(D/E) inhibitory motif (**Figures 2**, **3**). However, the CD4-1 cytoplasmic tails also have another highly conserved motif near their C-terminus, which, with the exception of in Polypteriformes, includes a tyrosine, namely P(K/Q)P(K/R)(A/G)FY(H/K/R). To the best of our knowledge, this latter motif has not been recognized before and the function is not known. When the sequences encoded by the last coding exon are compared, length similarity and the sharing of some individual residues of this motif are observed between ray-finned fish CD4-1 and shark CD4 (**Figure 3**). On the other hand, these regions in CD4-2 in ray-finned fish and in CD4 in Sarcopterygii show a lot of variation in length and in residues (**Figure 3**). Regardless of the function of this C-terminal motif in CD4-1, its presence concludes that CD4-1 and CD4-2 in ray-finned fish should have different functions.

Channel catfish and zebrafish LAG-3 (Dare-LAG3 and Icpu-LAG3 in **Figure 3**) cytoplasmic tails have an YxxME motif without the usual leucine at position 4, but this may function as an inhibitory motif nonetheless (see below).

### 3.5 Unusual LAG-3 in anurans (frogs) and a *CD4*/*LAG-3* hybrid in lungfishes

**Figures 2**, **3** show that the cytoplasmic tail of LAG-3 in the frog *Xenopus tropicalis* lacks the LAG-3-characteristic motif (F/Y)xxL(D/E). This seems to be common among frogs (Anura) as it is also observed in LAG-3 of, for example, American bullfrog (*Rana catesbeiana*; GenBank GFBS01039613) and strawberry poison frog (*Oophaga pumilio*; GIKS01213066) (not shown). In contrast, in LAG-3 of amphibians belonging to Caudata (salamanders) or Gymnophiona (caecilians), such as for example Hokkaido salamander (*Hynobius retardatus*; LE143073) or Cayenne caecilian (*Typhlonectes compressicauda*; GFOH01013653), the (F/Y)xxL(D/E) motif can be found (not shown). Why LAG-3 in frogs lost this motif can only be speculated, but it may have to do with the unique immune challenges that are encountered during body metamorphosis from tadpole to adult (77).

Another unusual observation in primitive Sarcopterygii was made in lungfishes. Namely, in the genome of West African lungfish (*Protopterus annectens*) a third *CD4*/*LAG-3* family gene is situated between *CD4* and *LAG-3* (**Figure 1F**). The sequence is a bit unusual, and more information is needed to be conclusive on the expressed form, but it seems to be an evolutionary relatively young hybrid form with a CD4-like ectodomain and a LAG-3 cytoplasmic tail (**Supplementary File 10**).

The presence of CD4 in coelacanths, lungfishes, and frogs, and of LAG-3 in frogs, have been described in article form previously (71, 78, 79), but those studies did not mention the above observations.

### 3.6 The CD4 and LAG-3 Ig-like domain sequences

Triebel et al., 1990 (1), already observed patches of specific similarity between mammalian CD4 and LAG-3, including at the start of domain D1 and the very unusual WxC motif in domains D2 and D4. These similarities are also shared with non-mammalian CD4 and LAG-3 (**Figure 3**). Similarities found at the start of D1 can also be found at the start of D3, and include, besides the frequent observations of G9, L14, and C16 (numbering as in human CD4), which have been recognized as consensus among Ig-like V category sequences (80–82), the frequent observations of (I/V)4, (F/Y)5, (A/V)12, and P15 (**Figure 3**), which also are not uncommon among other Ig-like V category sequences (80–82).

Overall, however, the sequences of the CD4 and LAG-3 Ig-like domains are not very well conserved. Barclay et al., in 1993 (53), already concluded: “The extracellular domains of CD4 have diverged rapidly in evolution and there is only 53% identity between rat and human sequences for domains D1-to-D4.” Between CD4 or LAG-3 orthologous sequences in widely divergent species, this percentage can even be lower than 20% identity ((37) and data not shown). Furthermore, the sequences show length variation between species, and, in each of the four domains D1-to-D4, for CD4 and/or LAG-3, species-specific cases can be found showing a loss of the Ig-like typical cysteine pair in β-strands B and F and/or the tryptophan in β-strand C (**Figures 2**, **3**) (1, 25, 82–85). Nevertheless, despite the variation in the Ig-like folds, the original folding of the CD4/LAG-3 family D1 and D3 domains should probably be understood as an Ig-like V category folding, and that of the D2 and D4 domains as an Ig-like C2 category folding; while for CD4 this is rather clear for all domains (75, 78, 79), the absence of V-typical β-strands C’’ and D in (mammalian) LAG-3 D3 domain (25) make this domain more difficult to assign to a category. However, in CD4 D1, CD4 D3, LAG-3 D1, and LAG-3 D3, the β-strands A only form β-sheet structures together with the β-strands G of those domains (25, 27) (in C2 category domains that would be with the β-strands B instead), which agrees with a V category identity; therefore, and also because of the sequence similarities between LAG-3 D3 and the other CD4/LAG-3 family D1 and D3 domains, we interpret LAG-3 D3 domain as a degenerated V category domain.

In structures determined by X-ray crystallography for CD4 and LAG-3, the connections between the D1 and D2 domains and between the D3 and D4 domains tend to (depending on the preparation) include a continuous β-strand structure overlapping the V domain β-strand G and the C2 domain β-strand A (25, 83, 84). This may contribute to the assumed rigidity of the CD4 V+C2 domain sets which have been described as rod-like structures (53, 84). The rarity of such domain-overlapping β-strands among Ig-like structures has been used as one of the arguments for D1+D2 and D3+D4 being derived from an ancient duplication (53). Important functional flexibility of CD4 is believed to derive from the hinge region between D2 and D3, and the region between D4 and the transmembrane region (27, 32). The hinge point in CD4 between D1+D2 and D3+D4 centers near L177-A178 at the end of D2 (27, 32). **Figure 3** shows that the residue at position 178 is a small residue (mostly glycine) in most CD4/LAG-3 family members, agreeing well with a potential shared flexible hinge function in all these molecules. Experimental data also suggest that in mammalian LAG-3 the D2–D3 linker is highly flexible (25).

From structures determined by X-ray crystallography, the involvement in binding MHC class II of the human CD4 D1 domain residues K35, Q40, F43, L44, T45, K46, G47, P48, R59, S60, and D63 has been reported (31, 86), and mutational studies were in agreement with such binding function (86–88). Mutations of, for example, F43 and K46 had a big impact on MHC class II binding (87, 88). However, of the human CD4 residues known to bind MHC class II, only (K/R)46 is conserved to some degree among CD4 molecules in widely divergent species (**Figure 3**) (33); notably, the exchange of K46 for an arginine may not impair function as in human CD4 the exchange of K46 for R46 only had a small impact on the binding to MHC class II (86). In human CD4, the K46 main chain makes hydrogen bonds with MHC class IIβ S144 main chain and the K46 sidechain can also contribute to the CD4 internal structure by making hydrogen bonds with the main chains of residues in CD4 β-strand D and the loop between β-strands C’’ and D (31, 86). MHC class IIβ S144 is very well conserved among MHC class II in widely different species (89), and it appears probable that the ancestral CD4 – MHC class II system already included a CD4 K46 or R46 residue that bound MHC class IIβ S144.

The CD4 residues F43 and K46 reside in β-strand C″ of domain D1 (30). The herewith matching region plus adjacent sequences in mammalian LAG-3, in which a β-strand feature is less pronounced or absent, has been named “loop 2” and in human LAG-3 consists of the stretch G85-to-P93 (25) (**Figure 3**; residue counting as in this figure). An antibody against this loop blocked the engagement of MHC class II (25) and an alanine replacement in murine LAG-3 of the final proline in this loop had a similar effect (90). On the other hand, a deletion of most of the loop in human LAG-3 (G85-to-R91) did not substantially affect MHC class II binding (25). Hence, it is not clear whether this region (loop 2 in LAG-3 and the C″ β-strand in CD4) represents a shared ancestral mode for binding MHC class II by the CD4/LAG-3 family. Mutation studies suggest that the unusually lengthy mammalian LAG-3 loop 1 (residues A52-to-R76 in human LAG-3) participates in MHC class II binding (30), but future elucidation of a structure of LAG-3 bound to MHC class II is necessary for a better understanding of the binding mode of these two molecules.

Human and murine LAG-3 molecules form homodimers through hydrophobic interfaces at the D2 domains (25). This involves the human LAG-3 residues W162, I164, F203, and F205 and corresponding mouse residues (25). LAG-3 molecules in birds and reptiles also have hydrophobic residues in these stretches, but in LAG-3 of more primitive vertebrates this is not commonly found (**Figure 3**).

### 3.7 Conservation of cytoplasmic tail activation and inhibitory motifs, respectively, in CD4 and LAG-3; the LAG-3 motif is similar to an ITIM

As shown in **Figure 3**, the cytoplasmic tails of CD4 and LAG-3 start with a stretch including positively charged residues (lysine or arginine). This is followed in human CD4 by an amphipathic α-helix in which the residues M407, I410, L413, and L414 form a hydrophobic side that participates in binding LCK (16). Judging from the sequences and software predictions for α-helical structures, this amphipathic α-helix organization is conserved to some extent throughout CD4 in Sarcopterygii and CD4-2 in ray-finned fish, but probably not in CD4-1 of ray-finned fish (**Figure 3**) (33). In shark CD4 cytoplasmic tails, the positioning of hydrophobic residues at 3-4 residues apart (an α-helix has ~3.6 residues per turn) between charged residues in a stretch predicted by software to form an α-helix suggests a similar propensity for forming an amphipathic α-helix. For example, within cloudy catshark CD4 (Scto-CD4 in **Figure 3**) the hydrophobic residues A404, L405, and L408 are predicted to be situated about one helical turn apart in a predicted α-helical structure that extends from the transmembrane domain to R409 (**Figure 3**). However, whereas the distance between these motifs and the Cx(C/H) motif is rather well conserved between CD4 in Sarcopterygii and CD4-2 in ray-finned fish, this distance is much larger in shark CD4 (**Figure 3**), so it is questionable whether the amphipathic helix can have the same function in LCK binding.

How the CD4 cytoplasmic tail motif Cx(C/H) is used for binding LCK has been discussed above. Notably, in CD4 in primitive species, this motif tends to be preceded by a tyrosine, the reason for which is unclear (**Figure 3**). It is also not certain why in CD4 of Sarcopterygii and CD4-2 in ray-finned the residue between the two cysteines tends to be glutamine (Q421), but it seems that Q421 together with other residues such as K417 and H424 may affect the folding of this region of the CD4 cytoplasmic tail (16) and the similarity in residue types at those three positions between Sarcopterygian CD4 and ray-finned fish CD4-2 suggest a similarity in their LCK binding mode that is somewhat different from ray-finned fish CD4-1 and shark CD4. Sarcopterygian CD4 and ray-finned fish CD4-2 are also similar in the apparent lack of stringent conservation of the stretch encoded by the last coding exon (in human CD4 the region R425-to-I433), whereas this region in ray-finned fish CD4-1 has a conserved length and a conserved motif that probably confers a function (see above). Among condrichthyan CD4 cytoplasmic tails, in a >30 aa long stretch N-terminal of the CxH motif, many residues are highly conserved, also suggesting an unknown function (see above; **Supplementary File 8**).

The cytoplasmic tail of LAG-3 was shown to be indispensable for the negative regulatory function of LAG-3 (22). Workman et al., 2002 (22), distinguished three motifs of interest in the mammalian LAG-3 cytoplasmic tail, in human LAG-3 represented by (1) RFSALE, (2) KIEELE, and (3) EPEPEPEPEPEPEPEQL (see **Figure 3**). They found that deletion of the second motif abrogated the regulatory function of LAG-3, whereas deletion of the third domain had little impact (22). Workman et al. (22) did not test a deletion of the first motif, but only exchanged its serine for an alanine, which had little impact. The important inhibitory function of KIEELE has not been confirmed in later studies (23). Maeda et al., 2019 (23), found that the FxxL sequence in the first motif defined by Workman et al. (22) was the most critical for LAG-3 inhibitory function, whereas for the EP-repeat they could only find an inhibitory effect if also the first motif was inactivated.

If comparing between LAG-3 in widely divergent gnathostome species, only the “first motif” distinguished by Workman et al., 2002 (22), is well conserved and can, according to our present study, be defined as (F/Y)xxL(D/E) and in many instances is (F/Y)PAL(D/E) (**Figure 3**, cyan shading). We already noted in 2010 (91) the similarity of this motif with an immunoreceptor tyrosine-based inhibition motif (ITIM) because in teleost fish LAG-3 the first residue tends to be a tyrosine and some sequences have a perfect ITIM consensus sequence (I/V/L)xYxx(L/V) (**Figure 3**) (92). ITIMs, like ITAMs, are docking sites for Src homology 2 (SH2) domains (92), and LAG-3 may negatively interfere with the phosphorylation of CD3 ITAMs by LCK—which contains an SH2 domain—recruited by CD4. In another molecular system, in an experiment in which the tyrosine of an ITIM was replaced by a phenylalanine, only a two-fold reduction in inhibitory function was observed (93), thus the FxxL motif found in many LAG-3 molecules (**Figure 3**) may very well exercise an inhibitory function as an “ITIM-like” motif (23). In support of such model, Maeda et al., 2019 (23), found that alanine substitutions of the F or L in this motif in mouse LAG-3 strongly reduced its inhibitory function, and Guy et al., 2022 (24), found that the presence of LAG-3 reduced CD4-LCK as well as CD8-LCK associations.

In LAG-3 of zebrafish and channel catfish (“Dare-LAG3” and “Icpu-LAG3” in **Figure 3**), an YxxM motif is found instead, but also such motif was found to interact with SH2 domains (94).

Interestingly, despite the high conservation of the glutamic or aspartic acid residue directly following the (F/Y)xxL motif ([D/E]365 in **Figure 3**), an alanine substitution of E365 in mouse LAG-3 did not lead to a loss of inhibitory function (23), and the function of (D/E)365 is unclear.

The motif 3 sequence in human LAG-3 (22), the EP-repeat, also appears to function through inhibition of CD4-LCK and CD8-LCK interactions. Namely, Guy et al., 2022 (24), found that the many glutamic acids at the end of the human LAG-3 cytoplasmic tail enhanced the dissociation of LCK from CD4 or CD8, and showed that this may be caused by competition of these negatively charged residues for binding Zn^2+^ ions that both CD4 and CD8 need for binding LCK with their cysteines through a zinc clasp structure (16). However, whereas in mammals this acidic stretch signature is well conserved, it is not so impressive or even absent in more primitive species. We therefore assume that, in evolution, the ITIM-like motif was the original inhibitory motif and that later in the development toward mammals the acidic stretch was added to increase the LAG-3 inhibitory effect on CD4-LCK and CD8-LCK interactions.

## 4 Conclusions

With the finding of genes for both CD4 and LAG-3 in sharks and basal ray-finned fish lineages, we now have established an overview of the sequence evolution throughout jawed vertebrates of these two related molecules with opposing functions. Their genes originated from a tandem gene duplication and in multiple animal clades have retained their apparent original gene orientation, although not in modern ray-finned fish. The most important conclusions of this study probably are:

*Sharks also have CD4 and LAG-3 genes*. Shark *LAG-3* was identified for the first time, and its gene location, sequence and expression pattern are in agreement with findings for *LAG-3* in higher vertebrates. A candidate sequence for shark *CD4* had been identified before as a *CD4*/*LAG-3* family member (38), but only with the here presented novel information on genomic location, expression pattern, and sequences from multiple shark species, could convincingly be identified as *CD4*. Although the present study is suitable for gene identification, only future functional studies can determine in how far the functions of shark CD4 and LAG-3 are identical to those in higher vertebrates. Unusual of the CD4 cytoplasmic tails of Chondrichthyes, including sharks, is their long length, the acquisition of an extra exon, and a highly conserved stretch N-terminal of the CxH motif for which the function is unknown. That shark CD4 cytoplasmic tails have a CxH motif instead of a CxC motif for (presumably) binding LCK does not suggest absence of LCK binding because CxH is also commonly found in CD8 of fishes (**Supplementary File 7**) and, previously, Zn^2+^-dependent binding was shown between relevant fish CD8α CxH-containing and LCK CxxC-containing fragments (57).

*The tandem gene duplication generating CD4-1 and CD4-2 occurred early in ray-finned fish evolution.* Previously, *CD4-1* and *CD4-2* genes were only reported for teleost fish. In the present study, we showed that also basal ray-finned fish lineages possess both. Moreover, for the first time, we showed that a difference between the two types of molecules is that CD4-1 has a highly conserved motif with unknown function, P(K/Q)P(K/R)(A/G)FY(H/K/R), at the end of its cytoplasmic tail.

*The ancestral, well-conserved inhibitory motif in LAG-3 cytoplasmic tails is an ITIM-like motif.* Conserved among LAG-3, from sharks to humans, but with the exception of frogs, is an ITIM-like motif in the cytoplasmic tail. Even, in some teleost fishes, the motif sequence is an actual canonical ITIM. The well-conserved sequence of the ITIM-like motif among LAG-3 sequences is (F/Y)xxL(D/E), and the function of the acidic residue at the end is unclear. Only in tetrapod species evolution, the LAG-3 cytoplasmic tail additionally acquired an acidic stretch (an EP repeat in mammals) that is believed to confer an extra inhibitory function by also targeting LCK.

CD4 and LAG-3 form one of the sets of similar molecules that are used to keep the immune system in balance by promoting opposing functions. Other examples are CD28 versus CTLA4 (95) and, probably, at least under some conditions, CD8αα versus CD8αβ (96). The special edition of collected articles in which the present article is published is dedicated to “Evolutionary Trade-Offs in Adaptive Immunity.” Within CD4/LAG-3 evolution itself, trade-off principles are not easily recognized, but the immune balancing principle that they represent is an important underlying factor for driving evolutionary trade-off developments in other molecules.

## Data availability statement

The datasets presented in this study can be found in online repositories. The names of the repository/repositories and accession number(s) can be found in the article/**Supplementary Material**.

## Ethics statement

The study on cloudy catshark was approved by the Institutional Animal Care and Use Committees of the Fukui Prefectural University (2023-F4-1). The study was conducted in accordance with the local legislation and institutional requirements.

## Author contributions

FT: Conceptualization, Investigation, Writing – original draft, Data curation, Formal analysis, Methodology, Project administration, Software, Supervision, Writing – review & editing. KH: Data curation, Formal analysis, Investigation, Methodology, Software, Writing – review & editing, Validation, Visualization. RM: Writing – review & editing, Data curation, Formal analysis, Methodology, Software, Validation, Visualization. YO: Resources, Writing – review & editing, Data curation, Formal analysis, Investigation, Validation, Visualization. AV: Resources, Writing – review & editing, Investigation, Data curation, Formal analysis, Validation, Visualization. MF: Data curation, Formal analysis, Investigation, Validation, Visualization, Resources, Writing – review & editing. DP: Resources, Writing – review & editing. KT: Resources, Writing – review & editing. HS: Funding acquisition, Investigation, Methodology, Resources, Writing – review & editing. JS: Resources, Writing – review & editing. JD: Conceptualization, Data curation, Formal analysis, Funding acquisition, Investigation, Methodology, Project administration, Resources, Software, Supervision, Validation, Visualization, Writing – original draft, Writing – review & editing.
