# Supplementary material for: CD4 and LAG-3 from sharks to humans: related molecules with motifs for opposing functions
Source: Front Immunol. 2023 Dec 21;14:1267743. doi: 10.3389/fimmu.2023.1267743 (PMC10768021; doi:10.3389/fimmu.2023.1267743)
Supplement: Supplementary file 1 [file DataSheet_1.pdf]

Supplementary file 1

Primers and their use for the amplification of *CD4*, *LAG-3*, and other genes in sterlet sturgeon and cloudy catshark

As described in the main text, we experimentally amplified and sequenced *CD4* and *LAG-3* in sterlet sturgeon (*Acipenser ruthenus*) and cloudy catshark (*Scyliorhinus torazame*). This file describes the sequences of the primers used for these amplifications in Table 1 (page 3). Figures 1 (page 4) and 2 (page 5) in this file explain visually where those primers bind in the sterlet sturgeon and cloudy catshark genes, respectively. Table 2 (page 6) shows the sequences of the primers that were used for the real-time PCR analysis of gene expression in cloudy catshark tissues.

Amplification of sterlet sturgeon CD4-1, CD4-2, and LAG-3

For cDNA cloning of the entire open reading frames (ORFs) of sterlet sturgeon *CD4-1* and *CD4-2*, primer sets *Sterlet-CD4-1-F* + *Sterlet-CD4-1-R*, and *Sterlet-CD4-2-F* + *Sterlet-CD4-2-R*, were used, respectively, each of the primers binding to a 5' UTR or 3' UTR. Notably, the primer *Sterlet-CD4-2-R* shares only 56% identity with the corresponding sequence in the sterlet sturgeon *CD4-2f* gene, and we did not detectably amplify *CD4-2f* sequences.

For sterlet sturgeon *LAG-3* cDNA amplification, we used forward primer *Sterlet-LAG-3-F*, binding to where the 5' UTR and signal peptide coding region connect, in conjunction with the reverse primer *Sterlet-LAG-3-R*, specific to the 3' UTR.

As for the genomic sequences, we were interested in the sequences encoding the domains 1 (D1) and 3 (D3) Ig-like sequences and the cytoplasmic tails, because gene-specific and/or species-specific differences in the intron-exon organizations of these regions are observed. Therefore, for each of the sterlet sturgeon genes *CD4-1*, *CD4-2*, and *LAG-3*, we developed specific primer pairs for amplifying the Ig-like D1 domain coding sequence (e.g., *Sterlet-CD4-1-Ig1-F* + *Sterlet-CD4-1-Ig1-R*), the Ig-like D3 domain coding sequence (e.g., *Sterlet-CD4-1-Ig3-F* + *Sterlet-CD4-1-Ig3-R*), and the cytoplasmic tail region (e.g., *Sterlet-CD4-1-CP-F* + *Sterlet-CD4-1-CP-R*) (Table 1 and Fig. 2 in this file).

Amplification of cloudy catshark CD4 and LAG-3

For cloudy catshark *CD4* and *LAG-3*, the full-length cDNA sequences were deduced from amplifications of partial ORF, 5' RACE, and 3' RACE fragments that overlapped with each other. Partial ORF fragments of catshark *CD4* and *LAG-3* were amplified with primer sets *Catshark-CD4-F* + *Catshark-CD4-R* and *Catshark-LAG-3-F* + *Catshark-LAG-3-R*, respectively. For 5' RACE of catshark *CD4* and *LAG-3*, 5'-RACE-Ready cDNA was amplified first with the respective *5'RACE-R1* primer and 10X universal primer A mix (*UPM*, supplied in the SMARTer RACE 5'/3' Kit of Takara Bio), followed by the

second round of (nested) PCR with the respective *5'RACE-R2* primer and the *Universal Primer Short* (supplied in the SMARTer RACE 5'/3' Kit of Takara Bio). Likewise, for 3' RACE of catshark *CD4* and *LAG-3*, 3'-RACE-Ready cDNA was amplified first with the respective *3'RACE-F1* primer and *UPM*, followed by the second round of (nested) PCR with the respective 3'RACE-F2 primer and the *Universal Primer Short*.

To examine the expression levels of catshark *CD4* and *LAG-3*, and, for equilibration, their house-keeping gene *E1FA*, we used the respective primer sets *qF* + *qR* for real-time PCR.

Table 1. Primers used for cDNA and genomic DNA cloning

Primer name	Primer sequence (5' to 3')	Purpose	Accession number
Sterlet-CD4-1-F	CAGCGARGGACYCTGTTTCCAGC	cDNA cloning	LC745920
Sterlet-CD4-1-R	GGTAAAAGYTGYCAGTAGAGGACCAG		
Sterlet-CD4-1-Ig1-F	AGGAATGTGTTTGGGGTCGTTG	DNA cloning	LC746166
Sterlet-CD4-1-Ig1-R	CTGTACTTGTTCTCTTTGCTACC		
Sterlet-CD4-1-Ig3-F	GACCCGCAGCCTGTAATCAGA	DNA cloning	LC746167
Sterlet-CD4-1-Ig3-R	CTCCAGATGCACCACGGATGTC		
Sterlet-CD4-1-CP-F	AACCCCATGCATAGAAGACCA	DNA cloning	LC746168
Sterlet-CD4-1-CP-R	CTGGCTGCAGTGTCGGAAGTC		
Sterlet-CD4-2-F	CTGTGTTTGGAAACGTGTTAGAAATC	cDNA cloning	LC745921
Sterlet-CD4-2-R	ATATACTCCATTCACTTCACTATAG		
Sterlet-CD4-2-Ig1-F	GGAAACATATACGTATCTAAGAAG	DNA cloning	LC746169
Sterlet-CD4-2-Ig1-R	CGACCAATAGGTTGTACTCTTTC		
Sterlet-CD4-2-Ig3-F	ATTCAAGCCGACCCTTCGTCTG	DNA cloning	LC746170
Sterlet-CD4-2-Ig3-R	CTCAGCACGTTTAACTGGAAGGA		
Sterlet-CD4-2-CP-F	CTGACTCGAAGAAGAAGGTTGC	DNA cloning	LC746171
Sterlet-CD4-2-CP-R	AGTCTGTTGAAGATTAATAGTC		
Sterlet-LAG-3-F	AATCATGCTTTGTTTATTCACTTTGCTTATTGG	cDNA cloning	LC745922
Sterlet-LAG-3-R	GACTVGCTCTCCACGGTTTCATTCC		
Sterlet-LAG-3-Ig1-F	GAACATATTTTCGCGGGCGTTG	DNA cloning	LC746172
Sterlet-LAG-3-Ig1-R	CATGACGTGCAGGTGAATTAAC		
Sterlet-LAG-3-Ig3-F	GGCAGCGTGGTGTACGGCAGC	DNA cloning	
Sterlet-LAG-3-Ig3-R	ACGCTCCTCTCAATCCTCTTC		
Sterlet-LAG-3-CP-F	GCAAGGGGGAATTTGCACTTC	DNA cloning	
Sterlet-LAG-3-CP-R	AGTCTGATTCATCACGGCTC		
Catshark-CD4-F	GATGCCGGCTATTTTGATTGTGA	cDNA cloning	
Catshark-CD4-R	TCCCTTTCCCCTGTGAGGAC		
Catshark-CD4-5'RACE-R1	CATCACCGTGTTCTCGTTGC	RACE	LC770928
Catshark-CD4-5'RACE-R2	CCAGCTCACCTTTCCTGCTG		
Catshark-CD4-3'RACE-F1	AAGGTCACCAAGCACGAAGC		
Catshark-CD4-3'RACE-F2	CTGGAGTGTGGATCTGCGAAA		
Catshark-CD4-qF	GCTCATTGTCTCCAGCGTTCA	Real-time PCR	
Catshark-CD4-qR	ATCACCGTGTTCTCGTTGCTC		
Catshark-LAG-3-F	CCGGCAACTACTCCCTCACC	cDNA cloning	
Catshark-LAG-3-R	GCACTGGACGTCACCCTGAC		
Catshark-LAG-3-5'RACE-R1	GCCATGTTCCCTCCTCTGCTC	RACE	LC770929
Catshark-LAG-3-5'RACE-R2	GTTGAGCTTGCTGCCCTCCAG		
Catshark-LAG-3-3'RACE-F1	CGGGGCACTGGATAGAGAGGA		
Catshark-LAG-3-3'RACE-F2	CTGGGACCTGTGCTTGTTGGA		
Catshark-LAG-3-qF	CTTCATGTGGTGCAAGTGACGA	Real-time PCR	
Catshark-LAG-3-qR	GCTCCACCATCTCACAGAGGA		

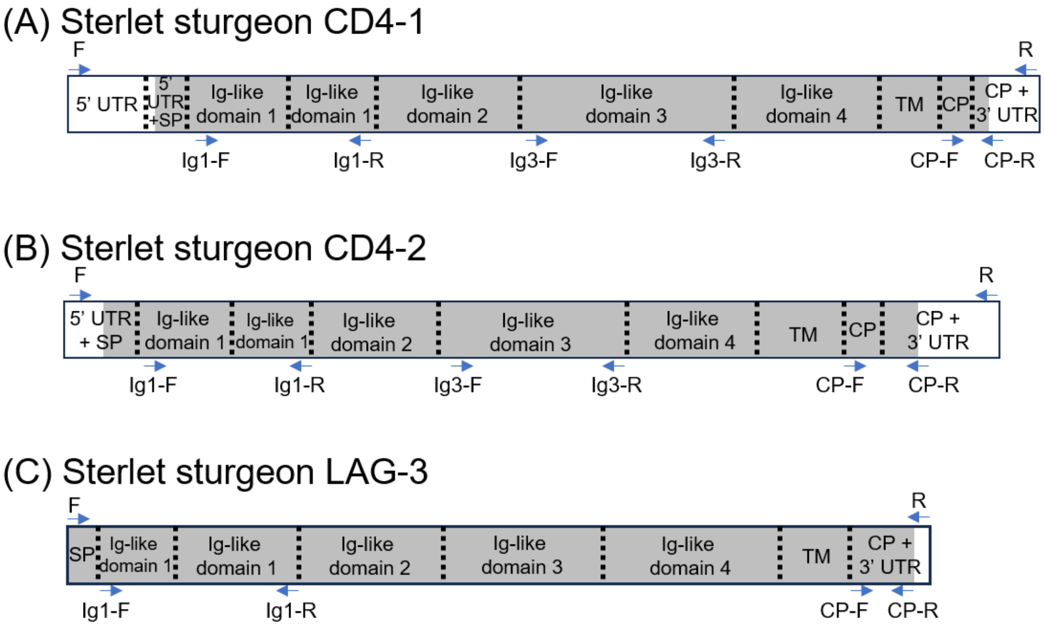

Figure 1. The applied strategy for cloning sterlet sturgeon *CD4-1* (A), *CD4-2* (B), and *LAG-3* (C). The large rectangle represents the cloned cDNA. The dotted lines correspond to exon borders, based on the information of GenBank accession GCA_010645085. White portions represent the untranslated regions (UTR) at the 5' and 3' ends, and the gray portions represent the open reading frames (ORFs). Functional domains encoded by respective exons are noted in each section. SP, signal peptide; TM, transmembrane; CP, cytoplasmic domain. Arrows above the rectangle denote primer positions for cDNA cloning of each gene while those below the rectangle represent primers for cloning DNA sequences from sturgeon genomic DNA. Primer names are shown above or below the arrows.

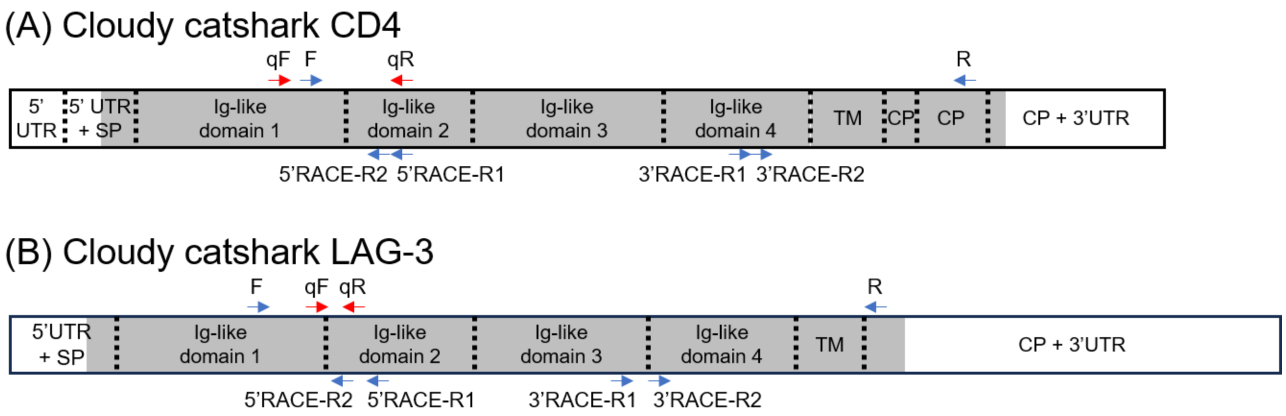

Figure 2. The applied strategy for cloning cDNA of cloudy catshark *CD4* (A) and *LAG-3* (B). The large rectangles represent the deduced full-length cDNAs. The dotted lines correspond to exon borders, based on the information of GenBank accessions GCA_902713615 and GCA_003427355 for *Scyliorhinus canicula* (smaller spotted catshark) and *Scyliorhinus torazame* (cloudy catshark), respectively. White portions represent the untranslated regions (UTR) at the 5' and 3' ends, and the gray portions represent the open reading frames (ORFs). Functional domains encoded by the respective exons are noted in each section. SP, signal peptide; TM, transmembrane; CP, cytoplasmic domain. Blue and red arrows above the rectangle denote primer positions for partial cDNA cloning and gene expression analysis of each gene, respectively. Blue arrows below the rectangle represent primers for RACE PCR. Primer names are shown above or below the arrows.

Table 2. Primer sequences for real-time PCR

Primer name	Primer sequence (5' to 3')	PCR amplicon size	Accession number
Catshark-CD4-qF	GCTCATTGTCTCCAGCGTTCA	231 bp	LC770928
Catshark-CD4-qR	ATCACCGTGTTCTCGTTGCTC		
Catshark-LAG-3-qF	CTTCATGTGGTGCAAGTGACGA	85 bp	LC770929
Catshark-LAG-3-qR	GCTCCACCATCTCACAGAGGA		
Catshark-LCK-qF	CAATACCAGCAACAAAACTATCCTG	162 bp	XM_038794700
Catshark-LCK-qR	CTGGTCACCTTCCTTGAAATCC		
Catshark-CD8A-qF	CCAAGAGTATTATGTGGAAGAGGTTG	224 bp	GCB70530
Catshark-CD8A-qR	TGCAGTAAACGATGGAGACGA		
Catshark-CD8B-qF	CTAATGAGCGAGCCCCAGAA	169 bp	GCB70529
Catshark-CD8B-qR	ACGTGTATCCATTGCCACTCC		
Catshark-CD3Z-qF	TCAGGTTGAAGAACAGGAACCA	202 bp	GCB69269
Catshark-CD3Z-qR	GCTGCACTCAGTCCCTGGTAA		
Catshark-TCRA-qF	CCTTCAGACCAGCAGGACAAG	173 bp	GCB76418
Catshark-TCRA-qR	AGACCCAGATCCGAAAGGTCA		
Catshark-TCRB-qF	GATCCGATCCTACTACATTCAGCTC	151 bp	GCB66423
Catshark-TCRB-qR	GACACGAATATTGCGTACAAGATG		
Catshark-PAX5-qF	GTCACATCCCATACTATAGCACCA	168 bp	LC495442
Catshark-PAX5-qR	TGGGTTTTCCTGAGCACCTTC		
Catshark-EF1a-qF	GGAGGCGAGTGCTAACATGAC	172 bp	LC258080
Catshark-EF1a-qR	AATACCGCCAATTTTGTAGACG		
Catshark-RPL13-qF	AGAAAAGTCCGCAGGCGCAA	64 bp	GCB78717
Catshark-RPL13-qR	CAACTGGACGTGGAGCGATG		
Catshark-ACTB-qF	GCCACTGCTGCTTCCTCTTC	141 bp	LC258081
Catshark-ACTB-qR	TATACCACAGGATTCCATACCCAAA		
