# Supplementary material for: CD4 and LAG-3 from sharks to humans: related molecules with motifs for opposing functions
Source: Front Immunol. 2023 Dec 21;14:1267743. doi: 10.3389/fimmu.2023.1267743 (PMC10768021; doi:10.3389/fimmu.2023.1267743)
Supplement: Supplementary file 2 [file DataSheet_2.pdf]

Supplementary file 2

# Nurse shark sequences used for RNA-seq and scRNA expression analysis

The following sequences of nurse shark (*Ginglymostoma cirratum*) were used for mapping to find gene-specific matches by RNA-seq for the nurse shark tissues spleen and thymus (Table 1 in the main text) and, most of them, also by scRNA analysis for isolated nurse shark splenocytes (Supplementary file 9C).

**CD4** (from GenBank GIWU01184608; starting with the start codon and including 3'UTR)
```
ATGGACGTCCCCACGTCTCCCCTTGTTCGTCTTTGTGGGATCTTCCTGCTCCTGATCACAGCACTGCCTCCTGGTTCCCGG
GTCTCTCCCTATGTGACCGAAGGGGACACGGTGTACGCCTGTCAGGGGGAGACCATCACCCTCCTGTGCCAGGTGCCCGAC
GTAGTGTCCAGGCCCATCACCAGCCCCCCGGGTTTCTGGAAGTGGACCTCAGCAGATGGCACCGGTACCACCCTCACCATC
CTCCAGTACCTGAGCAGCGTTCGGAGCAACAGCTTTTCCAAGCTGTCCGCCCGCAGCCGGATCTCCGAACGTCGCCAGTTC
GGGAACTTCTCCCTCCTGATCTCGAGCCTCGATCGGTCGGACTCCGGAAGTTACTCCTGTGAGTTCAGCTTCGGGAGATCC
CAGGCCAGAGCGACGTGGCAGCTCCGAGTGATCGAAGTGAAAGCGACGATGGCGAACCCGCTGATAGAGACCCAGCGTGTG
GAGCTGACGTGTGAGGGATCTGTGCAGAACGTGAGCTGGTCCGGGCCCCTGGGGCCAGCTGGGAAGGGGAGAACCCTGGCC
CTCAGCAACCTGGCAGTGCAGCACCAAGGAGACTGGGTTTGTACCTGCTGGTTTCCAGGAGGGACTGTCCAGAGCAGGTAC
CAGCTGGACGTCATAGGTCTCAATGAACCCCTGGATAAGCCTGTGTTCCTCCCCGTCTCCTCGGCCTTCCTCCTGCCGTGC
CGCCTGAACAAGGCGCTCCTCCCGCTGAAAGCTGCCTGGTACCAGGACGGCCAGGAGCTGATCACACTGAAAGCTGACAGT
GTCACCAAGACCTGGTCGAAACCGCAGGTCCCGTGGGTGCTGTTCAGCAGTTCCCAGCCCATCACCAACCTCTCGGTGATG
GTGCGGGCAGTGACGCTGGCCCAGGGCGGGACGTTCGAGTGCAGGGTCACCCTCAAGGGGGTGACGATACGAAGGATGGTG
AATGTGACGTTGATCGAAGTAAGAGGGAGTCACCCTACGCCAGTCCCTGTCGGTACAAACATGTCCCTCGTCTGTAACGTC
TCGAGCCACAGCGGCCAGACAGGGATACGGTGGAGGAGCCCGAGCACGATGGAGGGATTGGAAGACAGGAGGGTCCGAGGA
GAGGGATCTCTCCTGATCCGCCTCATCGAGGTGACCCAGCGCCACGTGGGTGACTGGATCTGCGAGATCAGCCAGGGCGAT
CAGCTGTGCGGGCAAGGGACCTACTCCCTCAACATCACAACCCTGACCTTGAGCGAGTTCGGGGACCCGCCCTTGGTCCTG
ATTATCGCAGCCTCTGTCGGGGCATTTGTTCTCCTCCTGTTGGCCACCGTGATCGCCGTCTGCCTTTCCAAGCGAGCTCGG
AGACGGAGACGGGCCCTGAAGAGACTGAGACATCCGCTGTGTCGGGAACACAGTTACCAGCTCTCGAACCAGCCCCTGTGT
CACAGCAACGACTACACTCCTGGTGATCGGCCCCTCCCTCCACCTCCCATCCCCTACTGCCCTCACAGGCAGCCTCGAAAG
GGTCGCCCCTCACATGCACGAGGGAGTCGGCACGCCAGGAGGTCACAGTTCGGCCCATAAGGTCGGGCGGGGGATCGCTCG
TCGGGGATTTGACCCCTTCCCCCCCCCTCCTCCGCCTCTCTCCCTCCACCCTCGCCGACCCTCCCGGCTTCCCGTCTCCTT
CTGCAGGGACACCGCACGCGCACCGGGCCTTCACTCGACGCCGGCCCCCATCGCTTCCAGCCGTTCAATAAACCTCTGCGG
GAGA
```

**LAG-3** (from GenBank GIWU01175563, SRR19353676.104273375, and SRR652971.40603362; starting with the start codon and including 3'UTR)
```
ATGGCTCCCCTGTGCCCCTGGATCCTCTCCCTCCTCGCTGTGGTCCGACTCGGGGCTGCCTCACACTCCGTCTCTCCTGCC
CCAGTGCTGGCTGCTGCGGGATCCACCGCCCGACTCCCCTGCCCGCAGCGCCGCCTTCTCCCCTTGGCCGCTCGCGGCTTC
CGCTCGCTGTCCCTCCGCTGGGAGGTGGCGCGAGGCTTGGGCATGGGCCCCTCACCAGGCTGGAGCCCCCTGCTGATGGTG
GGCAGCGACGGAGTGGTGAAGAGGGGCCTGGACCGCAGGGGCCGGAGGGCGGCCGTGTCCGGGGAGTGGATCGAGGCCGGA
GACTACTCCTTGCAGCTGCGGGAGGTGGCACTGCGCGACGGTGGAACCTTCCGGTGCCACGTTCGTCACCCCCGCCTCCGG
GAGGAGGTCAGGCTGGTCGTGGCGCAAGCGTCCCTGGAGAGCAGGCAGATGCCGGTGGAGGGCGAGCGTCTGTGCCTCCGG
TGCCAGGTGTCAGAGCCCCTGCAGGGGCTAGGAGTCTCGTGGTGGAAGGGGGGCACCGTTCCCGTGGGGAACGGGCACCAG
GTGTACTTCAACGACAAAGGATGGCGGCTGTGCATCGTCTCGCTGCGGAGAGAGGACGCCGGAGACTGGGCGTGCCACGTG
GAGTACCAGGGGCAGAGGGCCGTCGCCCCGGTGCCTCTCCGCATCTACGGATTCACCGTCCCTGAGGAGACGGTGCAGACG
TTCTATGCCCGCCCTGGTTCCAGCGCCCGCCTCTCCCTCGCCATTGCTGGGGGGCTTCCCGCCGAGGGCGGCTGGCAGCGG
GGCCGAGGGTCCGACCTCGGGGGGCTGGGCGAGGCTGGGGCCCGGGCCCGCACCGTGCTGGGGCCCGAAGGGTTACACCTG
TCCCTGGCGCCTGCCCTGGCGTCGGACCGAGGCTACTACACCGCGTATCTCAACGTGTCCGGGATCCTCGTGCAGAGGGCC
GTGCGGCTGGAGGTCATCGAGGTGACAGCCAGCAGGAGCGGTCCCATCCCCCTGGGATCTTCCTTGACGCTGAACGTGAGT
AGCACCTACCCCGAGGGCCTGGGCAGGGCCGAGTGGGAGCATGAGAATGCCAGCGCCTGGACGGAGGCGGAGGGCCGCGAC
CTGTTCCGGGTGGAGGGCCGTGCCCTCTACATCCCCCGCGTCACGCTGGCACACGCAGGCAACTGGACCTGCAACCTCTAC
CGGGGAAACACAACTGTGGGGCAACTGGGCTATCTCCTGGAGGTGACTGCGATGGATTACCTTGGGGCAGAGCTGCCCCCC
TCCACCTCGAGGCGCGCCACCCTCGCCGCGGTGTTGGCCCTCCTCCTCGCGCTCGCCGCCCTCGCCCTCGGCCTGTTGGCC
```

CGCAAGGTCAAGGCCCGGCGGCGGCGGAACTTTCCGGCACTCGACGTCACCCTGGAGACGGCGAACCCCACCCCCGGCAAG
AAAGTCTGAGAAAGGGGACCCCCCCCCCCCCTCCAAACCTCCCCACCACCTCAGCCCCAACCGAGGGGGCTCCTGCCCTCC
CATCCGGTAGGCCCTGAGGCCTAAAGCCGAGACCTCACCCTCACCCTCACCCTCACACCCTCACCCTCACCCTCACCCT

**CD3Z** (CD247; from GenBank GIWU01168378; starting with the start codon and including 3’UTR)
ATGAAATTGAAGTGGAGTTGTGCCGTATTGTACCTTGTCACCAAATTACAATTATCTGATGCTGACGTTATGAACCTGGAT
GACCCCAAGTTGTGTTATATTCTCGACGGAATTCTCTTTGTCTATGGGATTGTAGTCACTGGTCTCTACCTTAAGCTGAGG
CTGACCAAAGCAAAAGGAAAACATGAAGATATTCCTGCAAACCAACCAAATACAGAAGACCAATATCAACCTTTGCAGAAG
AGAGATCAAGATGCATATTCCCACCTTGGTTTTAACAAAAGGAACCAGGACACAGAGGCTGGATTTGGAACAAAACATAGA
GACGGTGCTGCTCACAATGACACCTATTCCAAACTGAATCCTAAGAACAGAAGTGAGCCTTATGGTGAACTCAAACCGACT
CAGCAGCCCAAGCGAAGAGGAAAGGGAGGGAATGACATTTACCAGGGACTGAGTACAGCTACGCAAGACACATATGACCAA
CTGCAAATGCAGCCATTACCAGCTGTCCCTTCAGCCCCTCGTCGCTGAGATTCTGTGATTGATATTGGAACACCATGATGA
CAGAAAGTGAAAAACCTTGAGGATTAATTCCATTTTACATGCACCAAAGTTATTTCAGAAATTGTTCCCAACAAAGTTATT
TACTTGAAAAACCCAGTTTTTTTTACAATTTGCATGATTTAAAAATATCACTATAATAGCAGCACTCTGAGTGACAGTTTT
AATGCCTGAAACCTTATTTTCCTTCTATGGATCATGTTGTGTTGGTTGATGTTTTTCAGGTAACATGGAATAGATTAAGTT
TACACGCATCTTTGAAAGTTTTTTTGTTGCATTTTTGCATATGTCATACTTAAATCTTTCCAATACTGAAAGTATTTAAAA
GTAAAGGAAGCGAACATAACAAATGGAAATAGGCATTTTGAAAGATAACCAGCCTCTAATGTAAAGAGATGGTTTCAAAAT
TGAAGTAGGGTTTTCAGTCCTTCTCTGAAAACGAATTAAAATTTAATCTGAACAGTCTTATTTGGATTACTTTTGTATGAA
AACAACTACAGATAAGAAGCCATGTAAATACTATTAGAAATGCAAAGTAACAAGATGTTTTTAATTGTAAACATTTTTTTA
GCTGTCCACTGAATTCTTGCACTTCAATGAGATTGTAGAAGATGTAGTGATATTGTCTGAAATTAGGTTTTCAGATGTTGC
CTGGCAGTTATCTAGTCTCACTTTTCTTGTTGCGATAAAAGCAAAGCTCTCCAATTGAGAAGCAAAACAATTTACCCAGCG
TCCTCTAGACTTTAACATAGTCAAATAAAAAAAATCGCGATTAGTTCTCAGCAAATGATTTTCAGCACATCTAGGCATCCG
TTTGTCGCTAAACTTCCTAATAAAGATTTCAGTTTGGCTAGAAATTTTAGAGTTAATTTTAATTCCCTCCGTTATTTCAAT
ATTTAGGCTTTGATCTTAGGTTACCAAGCAACACAAACATACATTTTAATGAAAATACATTTATTTAAAATTAGATGATCA
GGCATACTCTAAACATGAAGGAAAAGAAAATTAACAACCATTTGAGGTCACCCAAGGTTCTCAGGGTTGTGTCCCTTTATA
CAGTATTAGGTTTTTCTGTTACTTACACAGTCTTCGGCCAAATTTTGTATTTAATACAGAAAATGCAATGGCTACCTAGCT
CATGTGAGAGTCACATTTTTTTTACATGAGCTATCAGGATCCGAAATACATTGCCTGAAAAGGTTATGAAAGCAGAGCCAA
TAATGACTTCCAAAAATATTTACTTGAAAGGGAAATATAGCAGTGTTACTGGGCAAGAGTGGTGAATCAGGACTAATTGGA
TACCTCTTTCAAATAGACCGGGGTTATTTTCCTTGTAATTGACAGGGTTGATAGAGATTTATTTGAGGTGTATGAAATGAT
GAGGGTCCCAGATAGAGTGGAAAGAAAGACTTATTTTTCCCAGGAGAGAGGTCAATAATGAATAGCATTGACTTAAAGTAA
TTAGCAAAAGCATTAAAGGAGGGTAGAGGAGAATGCTTTTCACCCAAAGGGACATGGGAGTCTGGAACCCACTGTATGAAA
GAGTAAACAATGCAGAAATCCTTATTGAACTTAAATAATTCTTCAATATGCACTTGAAATGCCATAGATTACAGGTTAATG
GACCAAGAGCTGGAAAGTGAGATTAGGCCAACTGAGTCTTTTTTTCTGGCAGTATCGACATGATGGGCCTAATGTGCTGTA
AATTTCCAATATTTCTTACAAACATGCTTTGATGGAATAATTCAATGAATAATTCAACTATTATTGTGGTTAAATTAAAGA
TCAAAATGATGTTAGTAATCCAGCTTCAGTTTCTGCTTCTGGGTAACATTTCCATAAGAACTAACTAACTCAACGACACCT
ACTACTAATCACCTTAATGTGCCTTACCAAACATTGTTTTGTTTGCCATCCTATTTGCTTAGTAAAGTATCTTCCTTTGTT
TTCTAATAATTGCACCAAATTCACCATAATCTTAATTGTGTTTTGAATGCACAGTAAAAAAAAGTTTGTTATAAAGTAATA
TATCCATGAAAAAACTAAATATTATTCATAGGCTGTGCAATTAGTTTCTAGTTTTGAAATTAATGCATTACCTGAAGATCA
TAAAAAGGAATGAACAGATAATCTTCATTCTCAAGAAATGTAACACCCTGTTCGATTTTTAAAATTTGAACAAATAATCCA
GGAGGCAATTTGTTAACTTATTTTGAATGTGGGCCTAATTGATGAGATATGATTTTTTTTTTTGCTTTGTTTTATGGTTGTT
TAAAGGAAATTTTAATCATTGTTCATTTTACAATTTCATGTATTTAAGACGTAATGTTGTCCGTGACACCTGTCTATTTTT
CTGTATTAAAGGAGTAGCTATCTAGATTGGTTAATAATCCTTTGATTCTTTACCCTTAAGGAGAATCCTATTTTTTGTGAA
GTCCAGTGGCTTTTCCATCAGCTGTCATAACCACATCAAAATTGATATCATTTTGAGAACAGTTAGAGCAACAAAAGGATG
CTTTATGGTAAATTAATCATGAGCTAAG

**LCK** (from GenBank GIWU01148855; starting with the start codon and including 3’UTR)
ATGGGCTGTAACTGTAGCACAGACTATGATGATGAAAACTGGATTGAGGATATGTGCGAACAATGCAGACTGGAATATGAA
CGAGAGCAGAAGCAATATCAGCAACAAACCCAGAAAGAAATACGTGATCCCCTGGTGAATTATAAAATACAGCCTCCACCA
TCTTCGCCCTCGATAGGGGATGATATATTTGTCGCCTTGTACAACTACGAGCGAACCCATGAGGATGATCTTGATTTCAAG
GAAGGTGATCAGCTAAAAGTTCTAAAACGGGAGGGAGACTGGTGGAAAGCACAGTCTTTAGAGACAGGACAAGAAGGTTAC
ATCCCATACAATTTTGTGGCACGGGCGCACACACTGGAGATTGAAACATGGTTCTTTAAAGATCTAAGCCGGAAAGAAGCT
GAGCGACAACTTCTTACAGCTGGCAATTCTGTAGGCTCCTACCTGATTCGAGAGAGTGAAACCACCAAAGGTTCTTATTCA
CTGTCTGTAAGGGACTTTGATTCACAACAAGGAGACGTGGTAAAGCACTATAAGATTCGCTCTTTAGATAAAGGAGGCTAT
TACATATCACCACGGATAACCTTCAATACTCTTCACGAGCTGGTGAACCATTATTCATATAACACGGATGGTTTATGTCAG
TGCTTGAAGAAACCATGTCAGACTCAAAAGCCACAAAAACCATGGTGGCATGATGAATGGGAGGTCTCCAGAGACTCGCTG
ACACTGGTGGAGAAGTTGGGCTCTGGGCAGTTTGGAGAAGTTTGGATGGGTTACTATAACAACTACACCAAGGTTGCCATT
AAGTGTTTGAAGACTGGAAGCATGTCTCCTGAGGCTTTCTTAGCAGAAGCCAACCTGATGAAGACTCTGCAACATCACAAG
CTGGTGAAACTCCACGCTGTGGTCACTGAGGAACCAATTTATATCATCACAGAATACATGGCAAACGGTAGCCTTGTGGAT
TTTCTGAAGACATCTGTTGGATCAAAACTCAACATATTTAAGCTCATTGATATGGCAGCACAGATTGCTGAGGGAATGGCC

TTTATTGAAAGAAAAAATTATATCCATCGAGATCTTCGTGCGGCAAATATCCTGGTTTCTGAAGAGCTCTGCTGCAAGATT
GCTGATTTTGGTCTTGCCCGCCTTATTGAAGACAATGAGTACACGGCAAGAGAAGGAGCTAAGTTCCCCATCAAGTGGACG
GCACCAGAAGCCATCAACTATGGTACATTCACCATCAAGTCTGATGTCTGGAGTTTTGGAGTGTTGCTTACAGAAATTGTG
ACATATGGACGAATTCCATATCCAGGTATGACCAACCCTGAAGTCATTCAGAATTTGGAAAGGGGTTACCGAATGCCAGCC
CCTGAAAACTGCACCGAAGAACTCTACGAAGTAATGATGCGTTGCTGGGGAGAGAAGCCTGAAGACCGGCCAACTTTTGAA
TATTTGCAAAGTGTATTGGAAGACTTCTTTACAGCCACTGAATCTCAATACCAGCAACAACCTTAGCAGTGCCTCATGCCC
TGATGGTGTAAACATCATTGGGGATTTAAAAACTCTGAAAATTATTCTCCCTGGAATTGGGTAGCATGGGATGGGGAAAAA
TTCAGATCACAGTATGATTGCCCAAATGCAAGGCAGTCTTAGACTGGTCATTTTATCTGGAGGGATGATTGCCTGCAGCTC
TGATATTGTTCCTCATTTCACCATGGGCATAGAGAAAGTCATTTTTTCACTAAGGTAGGGCGTTTGAAATGTCCTCATATC
AGTTCCAAACTCAGGTAGATATCTTTGCCAGGTCTGCAGTGGAAATTCCATTTCTAATAGATAAATAAGATTTCTGACACT
TTTTTTTATTCTTTCATGGGTTGTGGGTTTTGCTTGTTGGGCCAACATTTATTGTCCATCCTTTAGTTTCCTTTGAGAAAG
TGGTGGTGAGCTGTCCTCTTCAATTGCTGCCATCCACTTAGTGGCCAAAGGTGTTCATTCAGTGGCCAAAAACAGTTCTGA
GGGGACTCCTGGCCATTATCTCATTGATTGATTAATTTTATCAGGAGCACCAACCTAGACACTTTTAAAAGATTTGTTTAG
AACACCATTGCATAGGTTTCATATAACAATATTCTATCAAACACAATCAGAATGTTGATTAATTGCACGAAACATGTCTTG
AGTTTAAAGCACATCCTGGTAAACATTTAGCTCCTTTTGCAGGTTATCCATTTGGTAAAGGGTGTGCTTCCAAGGATTTAG
CACCCAAATGGCTGACTTGGATTTTCCACATGAAGTAGTTATTGCAATTACTTTAAGGGTCCAATAATTGTTCTATCATCC
AATTGCTTTCAGTTATTGTTCCCACCTTCAAACAGAATAATTATACATACTGAAAGAAAACTCAAATTATTCAAAATATTC
AATTATGTTCTGGTGGCCCTAGGTCACCATTTTATTAATTTAATGTAGCAATCTTGATGAATGAAATCTTTTAACTGAAAT
ATTTATGCTACATCATCTATGTTTTTTTTAAGATTGTGCTTTTGAAAGAGTTTTTAAGATTTTCTTTTTGTTAACCTTTTA
GAATTAGCTCATGTCTAAGAACAGGCTACTCTGCATAGTATCTGCCCAGATTGTTCCCAAGCAGACCACTAGACAGAACAG
CCTTTAAGCTCTCCAATACAGGCCCACAGTCAATAGGTTGGATAGAAGGAACATAAATCTCTATTTGAAGTTGTGCAACCC
CTTCTTAGAGTCGTGTACATACTTCAATCACTGGTATGGGCATTGCTAGTAAAGCCAACATTTATTGCTCATTCCTTGAGA
AGTTGGTGGTGAATTAGCTTTTGGAACCATTATTGGGTAGGGACACCCACTGTGCTGCTCGGAAGGGAGTTCCAGAATTTT
GACCCAGCAAACATGAAAGAATGTTGTTATGGTTCAAAGTCAGGATGGTGTGTGACTTGGAAGGGAACATGCAGATGGTGG
TGTTCCCATGCAAGTGCTGCCCTTGTCCTTCTAGATGGTCGAGGTGACAGGCCCAGAAAGTATTACCAAAGAAACCACAGT
GAGTTGCTTCATTGTATCTTGTAATGGTAGACGCTGCTACCAATGTGTACTGATGGTGGAAGGAGTGGAGGTTGAAGGTGG
TGGAAGGAGTAGAAGTTGAAGTTAGTGGATGGAGTGCCAATCTAGTGGGCTGCTCCTTCCTGCATAGTATCAAGCTTCGTG
TATCTTTTAGTGCTGGACCCCTTCAGCTGAGTGAATTATTTCAGTTTTAGCTTCTTGAAATGAAGCTTTTCCTCGACATGA
CCTAAAAGATGAGTCCACCCTGATGACTAAAAATAATAGCAGCTTCCATTATATTGGGATTGAAAAGAATAATGTGGCAAT
ATTTTACAGGTAATGATGTTGAATTCCACGCAAGACACAAAACTATTTTATCTTTTCCATGAAATAACAGTTTTGGCATTG
CAGTGATATCTTTGGTTTTGATTCAGGTAAGGTGAAAGGGAATTAATTGGAGGAAAAATTCATGGAGAGCAGGAAAATCCA
TTTGCTTGATATCTGAAATCTGTGCGCATTTGTGTTATGTGAAGTGAAACTGATACAACCTTCTCTTCTTGGAAGAAGTAT
CTTCTATTTCCTCACAACAAAACGTCACTGTGATTGCTATCAATAGATGAGGGGTACCCTTGGTGGCTCTGCTTTTCCAAT
TAATGGATGTTTCCTGAGAACCAGAGGTTTCACAGCTGAAATTGAGAGGAAAGTTTTTTTTAAACTTCATTGGGCTGATTG
GATAACAGATTTGTGTTTTATTTTATATATCCACGGGATGAGGGCATTTCTGGCTAGTCCAGCATTTATTGCC

**CD8A** (from GenBank KC707917; starting with the start codon and including 3’UTR)

**ATG**AAAATTCTTGTATTCCTCCTCGCGATACAACTGACTGGCACCGTCGTATCGCCTAAATTGAAAAAAGCGTCGGTGAAA
AAAGGGGAAAAGTTTGAAAATACTTGCTCACTGGATGCAGACGAGGGAGTTTATTGGTTCCAACAGCCCAGGAATTCCGGC
CCGAAGTTCTTGTTGTATGTAACAGGCACAGGTAAACCGAAAAGTGCGAGCAACCCCGAAAGACACACTGCAGGGAAATCA
GCCAAAAAAGTGACTCTGACGATCAAGGAGTCTGTGGAAGAAGACGAGGGGAAGTATTACTGTTTCATGGTCAAGAACATG
GTCATGATGTTTGGGGACATCACTGACCTGGACATTGAAGGGGTCGCAACCACGCCAGAGCCAACGACCATTCCCACCACG
ACACAAAAAAATACCAATTACTACGGACAGCAAAGGATCTACTCAATGTCATTCAACTAAAAGAGAGAAAACAGAAGATCCA
TTGAGCTGCCACTTTATCTTCTGGGCTCCTCTGACTGGCGCTGCCGCTCTGTTGCTCATCGCACTGACCAGTGTCTCCATC
GCTTATTGCAGAAGACCCCGCCGGAGACGCTGTCAGCACCAATTTCGAAAGAGACCAATAGCTGAAGAAGATAGACTATCA
AACAGATATCTT**TAA**CAAGTTCATTCTGGAAAACGTGTGCCATTCATCCATGGATCATTGAACCTTCTGAGAGATAAATGA
TGTGGCGTGGGAGTGACTGAGGCCCTCAGCGTGTCTTTCTATTTTGCACAATGGCCATTCCAATTGCCCCTTTCATCGTTT
GGAACATTGCCGTAGCTGACTGAAGTCAATACGTGACACACACTGGGTTTTGGGAAGAAAGTGGCAACTGTTCTAAATGTA
AACTTGTCCTTTTCTTATATCTGTGTTTGACAACAATTCCGCCCTAGAGGAAGACGCTTTAGTTTACATGGTACCTTTTT
GATATCTTTCCCAAATGCCTGGTCAAAATGCGGAGTGCCACTTGGAGGGTGAAAGCTGCACTCTCTCTCTGTATCCTGGTG
ATCTAGTACTGCCAAGGCAGTCTGTTTCATACCCAGGAACAGAATGGCTGGGCATGGGGAGTACAGCTCTACTGTAAAGGA
GGAACCTTAATGTTCAATTTAAACTCCTTCCAATCTGACTGTTCCTGCTGTGGTCACAGCCTCCAGAATGAAACAGATACC
AATCCCAAGGAGGGTTTTTTTCAATATTACTTCTTCACAGGATGTGGGCATCTCTTGTGCAGAGGGCAGTTAAAAATCAAC
CACATTGTGTGCATCTGGAATCACATGTAGGTAATATAGTGTGGAGCTGCGGGAGCGCGGCGGGCCGGGCAGCGTCGGAGG
GGCGGGAGGGCTGACGTTTCGGGCCTAGACCCTTCATCAGAAATGGGGGAGG

**CD8B** (from GenBank KC814635; starting with the start codon and including 3’UTR)

ATGTACCTTCTTGGCTACATTTGGATGCTGGGCTGTTGGATTGAAAGTGCCCAGGCTGAATCAATCCTACAGCAGACTCCG
AGAGAAATAGTGACGACCAAAGGCGGACAGATCAAACTCTCCTGTAATCTGAAGAAGGGCTCACTCGAAGAGATTTTCTGG
TACAAACTAGACGGAAACTCCGAGGCCCATTTTCTGAAGTCTGCCAGTATCCTGAACAAACAGACCTCAGGAGAGGGAATA
ACCGAACGGTTCGTTTTAACCAAAGATACCTTTCGACTGTCGTTCAGTTTAAGCATCCAAAACACGCTGCTGTCAGACAAC
GGCACCTACTACTGCCTGATGATCAAATCCTACTCCATGTACATGGGAAGTGGAACTGCGGTCATAGTAGTTCCAGAACAA
GAGAAAGTAACTTTGCCGCCTCCAACGACTAAAAGCGGCGGAATCAATCGCGTTACCCAGCCCAAGGTACGACCGAAGAAG
AAAGCGGGGAGTCACGGGTACGCGTGTAACTGGAGTATCTGGGTCTCTCTTGCCGTTTGCAACCTCATGCTCCTGACCTCC
GTCATCTTTGTTGTTATCAAACACAAGATCCAGAGTAAGGGCTGGAGGCGATGTCCCCACCAGCTTCGAAAAAGGTAATTA
ACACATCAAATCATGACTAACGAGTGGAGGAAAGATTGGAATGTTATTTCAACCAATTGTGAACTTTTGAAAGTCATAGTC
ACAACTTTATATTGATGAACACAGTTTTGTCTACATTTCACCCTTTTGTATCATTGCTGTAAAATCTCTTTTGGTTATGAA
AACTGGGATGAAACTTGCACTCAAAGCATACACATTCTGTGAACCCTACTCCCAACCTCCCACTCACTTCCATGGCCATGT
AATCTCTGAGGCACATCTCTGCACATCTCTGTGCATACACATACACATACACAGGGTAGCATGATGGCACAGTGGTTAGCA
CTGCTGCCTCAC

**TCRA** (from GenBank GIWU01138746; constant region and 3’UTR)
GACTCATCTGAACCATCCGTCTACATTCTCCCTCCGTACGACAGCGATACCAAAAATGCTGCATGCCTGGCGACTGACTAT
TTCCCCCAAAATGTCAGCATGGTCGTGGCAGCTGGGAATAAGAAGCAGAAGCAGGACAAATCGAAGGGCTTGCTATCCACC
AATGATCGCAGTTACAGCCTGACAGGATTCCTGGACAAATTGGAGGACCCCAATGACTTTACCTGCCATGCTGGGAATACT
GTCAAACACTTCCCAACAGCTGATCAATTGAAGTACAGCTGCATTAATGTGGAGGAAGCGAAAGGAGATCCACAATACAAC
CTTCTGTCACTGACCGTCTTCGGATTGAGGATCCTGTTTGTAAAGAGCATTGTCTTCAACATGCTAATGACTATCCGCATC
TGGGTCTCGTGAGCTGGCCCTGTGACTGTGAACACTGGATGAAAAGGTGCGATTGCGTTGTTGAAGAGTGAAAAAAGAAAT
TCTTCACCTGAAGTCTCAGCTAACTTCGCTTGGATTGTGACCTCTGAAACTGCAATGCCACAAAAGCAATACTTTGCTTGT
AAAAAAAAACCAGATATACGTGTTTGTGTGTGGTGTCTTTGTATATGCCTGTTTACTCGTCCCTCCTGGGTCCTCTGTGAA
TGTAAAATCGAGTATTGTCCATCAGAGCCTTGTTGAGTTATCGGTCTCCTGCATCTTCAGCTTCTGGAGAATTTCAGCACA
TTAGCTCACCTCTCAGACACGCTGACTGCAGATGAACGGGGAATTGGAGGGAAATCGTAGAATCCTTCAGTGCAAAAGATC
ATCCCACCCTATGTTGTACACCACCCCCAGCCCCTCTCCACCCAGTGTTGGTCTAGCCAGTGATTTCAGCTGTCCCCAGCA
CCTCCCCTTTTGAACACCACCATCCCAATAGAGACCAGGGAGAAAATCCACTTTACCCTGCAACCCCACAATTCTCCCTGC
CTTTTGCCCATGTTCCTCCTGCACCTGAGATGCTAAATCTCACCACCCTCTTCTTCCTGCACCTCAAATCTAGAAGGGCAC
CCCCAACTTCTGCCCCTGGAGAGAGATTCCCCGGCACATCCCCAGAACCCCCTGTCAATTCACCCCACACAACATCCATCG
CACCCCTCCCCACCAAAGGATTTATCTCTCACTCTGAGATTTGATGCCACTGGTTAAATTCCAAAATATCAGAGATGGATG
TGCTCTCAGTCTTTGTTCCCTGAGACAATTTGCCAGAACTTTTTCCAAGTGCGTGTTTGTATGTTTGTGTAAATACTTGAG
CTGTAAAGGAAAGCTGATGTGTCAGAAAAGACCATTGTGTACCAAATGCCGATGTAAATTTAGCTGCCGTGGGGAGATTGT
TTGCTGTTTTAGATGTTACGGGCGTCAGTATCATCGTGTTTCACAAAGAATTTCTGTCAGCTTTACAAAGCTTGCCATATC
TTTCTCATTGCACTTGCCAATAAAATGTACCTTTTACGATCAAAC

**TCRB** (from GenBank FJ513763; constant region and 3’UTR)
CACCCGGTTAAATTTCCCAAAGTCACGATCCTCAAACCATCACCAGCAGAGCTGAGAGAGAAACGGAAAGCGACCGTGGTG
TGCCTTGTCACCGACTTCTATCCCGACAACATCCAGATCCACTGGTATGTCAATGGTGAGAAGCAGCCGGAGAACGACGCG
AAAATTCAGTCAGACCCCGAATCCATCATACAGGAAGACAGTAAATCCTACAGCGCCAGCAGTCGGATAAGATTCAACGAA
GAACAATGGCTCGAGTTGACGAAAGTTGAGTGCAGAGTTAATCATTATACAAATGGATCAGAGCCTACTTCGTACACGTCG
CAGTTTTACGTCAATGCTGAAACCTGCGGCATGAGTAAAGAAGCTAAAGCCCAGAGCATGACAACAGCCAGAATGACGTAC
CTTATGGTACTTTGCAAGAGCATCCTGTATGCATTGTTCGTGTCAATGATTGTTTGGAAGTCCAAGATCTCTCACAGCAAA
AGGTTTGACTGATCGCATTGAAGATGGTGGAACACATGAAACAGTATAGAGGAAAGGGATATGCACTTTTTGAATACTTAG
AGTGATTTGATATCAACTACTTCTGCAGTTCAAGCATGATACTGACAAGTGATCTTCAACATCGTGGCCATGCGTAATAAT
GGAATAGAGTTAGAGTGTTACCTAATTTTTCAGGAGTCGATATCTTGATGTGTCGTTATGTTCTCACGAGATAAGCAAGCT
TCAGTTTTTTGGGTTGCAAAACAATTAGGCAATTAATTCTCTGGTTGCCAATTTGAACGTGGGATTATTTGAACCTTTACA
CATAATCATAAACTATTACAAGGCAACGAGGCCCATTTGATCCCTCTTGCAAAAACATTCTCTGAGTGTCAGCCATTGAGT
TCAAAAATTTCAAAGGCAAAAACAGCTTTATGCTCAATCTTATTTTTTACCTTCTCTGCCCTCCTCTTTGATTCTGTTATT
ATAATGGCGCATATATTATAAAGTTGAAAGTTGAAAGTGATTCAAATGCTTTTTGAAACTATCTGTTTTTGTTCTCTCTAA
TTTCTGATCTTGCAGCAATGTATTATGAGTTTTAGAATGATTTTATTTGATTATGGAAAAAAGAAAATGTATCATAATTCG
TTACGGCATCGTCAATGGAGCTGGCATTGACAACATGCTTGTGAATTTTTGCAGTCTTTCATCCTTTTTTGTGAAGAAGAA
ATAAATAGATTAACTTCCAAAT

**PAX5** (from GenBank JAHRHZ010000270, deduced based on GenBank XM_048594913)
AACACGCACgCACACCGAAACGGGGCAACTTTTCTTCCAATCTTTTCACAAAGGACTCGAAATCCCAGAACCAGCATGGAA
AAAAATAGCAGCGCGCTCAGGAGTGTCCGATCAGGGCACGGCGGAGTGAATCAGCTCGGAGGGGTCTTTGTCAATGGTCGC
CCTCTCCCCGATGTTGTCCGGCAGCGGATTGTCGAGCTGGCCCATCAAGGGGTGAGACCCTGTGACATCTCCAGGCAGCTg

CGGGTGAGCCACGGCTGCGTCAGCAAGATTCTGGGCAGGTATTATGAGACTGGAAGCATTAAGCCAGGAGTAATTGGAGGA
TCCAAACCAAAGGTCGCTACACCCAAAGTGGTAGATAAAATCGCCGACTATAAGCGCCAAAACCCCACCATGTTTGCCTGG
GAGATTCGAGATAGACTGTTGGCAGAGAGAGTCTGTGACAATGATACAGTGCCTAGTGTCAGTTCTATTAACAGGATTATT
CGAACAAAGGTGCAGCAGCCTCCAAACCAGCAATCGCCAGTGACaTCCCATACTATAGCACCAAGCACAGTGGCAGTGACT
CCTGTTCCATCTGCTGCCACTGACCCTGCGGGATCTTCCTACTCTATCAGTGGCATTCTGGGAATCACACCTTCCAGTGCT
GAGAATAACAAGAGAAAACGtGATGAAGGTGCTCAGGAAAACCCCATGCAGAACGGACACTCCCATCCGAGCCGGGATTTT
CTGCGGAAACAGATGAGGGGTGAACTCTTCACACAGCAGCAGTTAGAAGTGTTAGACAGAGTCTTTGAAAGACAGCACTAT
TCAGACATCTTCACAACCACAGAACAGATCAAATCAGAGCAGAGTGCAGATTATTCCACCATGGCGTCACTGGCTGGGGGA
CTGGAAGATATGAAgTCGAGTCTAACAAATTCAGCTGACATAGGGACCAGTGTtCCTGGACCTCAGTCTTACCCCATTATA
ACAGGTCGCGATTTGGCCAGCACGACTCTTCCTGGCTACCCTCCGCATGTGCCCCCTGCTGCTCAGGGCAGTTATCCAGCC
CCAACACTGACAGGAATGGTGCCCGGAGGTGATTTTTCTGGGAGTCCATATTCCCATCCACAGTATACGACTTATAACGAC
TCCTGGAGATTTCCCAATGCTGGATTATTAAGTTCTCCCTATTATTACAGCACAGCAACCCGAGGGTCAGCACCAGCAACT
GCTGCAGCAACCTACGATCGTCACTAG
