# Supplementary material for: CD4 and LAG-3 from sharks to humans: related molecules with motifs for opposing functions
Source: Front Immunol. 2023 Dec 21;14:1267743. doi: 10.3389/fimmu.2023.1267743 (PMC10768021; doi:10.3389/fimmu.2023.1267743)
Supplement: Supplementary file 3 [file DataSheet_3.pdf]

Supplementary file 3

Deduced CD4 and LAG-3 amino acid sequences in representative species

Page 1	Sharks
Page 5	Bichirs and Reedfish
Page 7	Sturgeons and Paddlefishes
Page 10	Gars
Page 11	Teleost fish
Page 15	Coelacanths
Page 16	Lungfishes
Page 16	Tetrapods

Sharks (Class: Chondrichthyes [cartilaginous fish]; Subclass: Elasmobranchii)

***Chiloscyllium griseum* (gray bambooshark)**

CD4

Source: GenBank GJPK01006420 (a TSA sequence)

Sequence:

MSPVVRISATLVLVIATIPPDSRVSPAVTVQGRVYARSGESVTLPCQTPYPESRPMTSPVGFWKTARDRDMTGA
AVVVQYLSNVRTSSSSELSQRSRVSERRQFGNFSLIVASVRPEDSGFFQCDFTFRDYRAKASVELRVIRVTATVRN
PVPEGSDVELRCEGSEGDVSWEGPPNSRQGLATGTTLTLRKVAVPDRGRWVCVCTFGPGKVQSEYTLDIVGFT
DPPHRSIFLLSRSVALLPCHLNQLPPTSLPLRVTWHRDGHPLMSLNASGYPWAWQSPGHPWVLAREPVPPTNL
SVGLRAVSLAQGGEYQCRASSSEITASRTVMVTVMEVSASDPGPVARGRNTTLRCQVSQNSTETLLRWRHSNQ
SQSPQVETARGSGSLYIHLLEVTQREGGQWICEMYMRDRLIGMGIYSLNITEPNLGQLGVLSLIQVIGIATGFLTFL
LILCVTIVCLIKRSRRRRRALKRLRHPLCREHSYQLSNQPLCPWSDYSPAERPLPPPPVRYCPHRQPRKGIPPQGRG
GRTSSRKGNGGR

LAG-3

Source: GenBank GJPK01187227 (a TSA sequence)

Sequence:

MDPHYLWILCILYFGKVVTPSPTLLLAAVGSPALLPCPHYLPKPSSRSPRPGFRALSLLWERARGPGTATQQAWS
PLLAVGSDGVVKRGLGRRSRRAEVTGERIEAGDYSLRLREVVVSDAGTYRCRLRRAHTRLLEEVDLIVINVSLEGER
ETPVEGERVCLRCQATRPIPGLRLSWTRRGVHPEDGGRGGDTEEEEAAGGGGSRVCLEAVRREDAGEWGCRA
QYKRRSVLATFTLRVYGFALPVHPVPTVYALPGSQAQLWLPAEPGLSLSPGEAEGGWLRPGHAEGDAGRAQAR
LGGGGLSLTLSPALASDRGLYTGYLNVSGLLVQRDVRLEIMEVLTSQKGPVTLGSWLTLNVSTSYPEGLDRVVWG
HQNASEGRRPDPYSLYLPHVAWGDAGNWSCTLYRNQTVVGNAVVFLEVTDMAPLGSDQPSSSPGRATLVALF
TLLVLLVVLSALAIARKVKGPGRNFPALDVNLDTSDTPNKKI

***Ginglymostoma cirratum* (nurse shark)**

CD4

Source: modified from GenBank AGN91182; confirmed by TSA GIWU01184608

Sequence:

MDVPTSPLVRLCGIFLLLITALPPGSRVSPYVTEGDTVYACQGETITLLCQVPDVVSRPITSPPGFWKWTSADGTGT
TLTILQYLSSVRSNSFSKLSARSRISERRQFGNFSLLISSLDRSDSGSYSCEFSFGRSQARATWQLRVIEVKATMANPLI
ETQRVELTCEGSVQNVSWSGPLGPAGKGRTLALSNLAVQHQGDWVCTCWFPGGTVQSRYQLDVVGLNEPLDK

PVFLPVSSAFLLPCRLNKALLPLKAAWYQDGQELITLKADSVTKTWSKPQVPWVLFSSSQPITNLSVMVRAVTLA
QGGTFECRVTLKGVTIRRMVNVTLIEVRGSHPTPVPVGTNMSLVCNVSSHSGQTGIRWRSPSTMEGLEDRRVRG
EGSLLIRLIEVTQRHVGDWICEISQGDQLCGQGTYSLNITTLTLSEFGDPPLVLIIAASVGAFVLLLLATVIAVCLSKRA
RRRRRALKRLRHPLCREHSYQLSNQPLCHSNDYTPGDRPLPPPPIPYCPHRQPRKGRPSHARGSRHARRSQFGP

LAG-3

Source: modified from GenBank GIWU01175562 (a TSA sequence); this TSA sequence was repaired for the leader sequence coding part by the overlapping single read sequences SRA:SRR19353676.104273375.1, SRA:SRR652971.40603362.1, and SRA:SRR652972.8875575.2

Sequence:

MAPLCPWILSLLAVVRLGAASHSVSPAPVLAAAGSTARLPCPQRRLLPLAARGFRSLSLRWEVARGLGMGPSPG
WSPLLMVGSDGVVKRGLDRRGRRAAVSGEWIEAGDYSLQLREVALRDGGTFRCHVRHPRLREEVRLVVAQASL
ESRQMPVEGERLCLRCQVSEPLQGLGVSWWKGGTVPVGNGHQVYFNDKGWRLCIVSLRREDAGDWACHVEY
QGQRAVAPVPLRIYGFTVPEETVQTFYARPGSSARLSLAIAGGLPAEGGWQRGRGSDLGGLGEAGARARTVLGP
EGLHLSLAPALASDRGYYTAYLNVSGILVQRAVRLEVIEVTASRSGPIPLGSSLTLNVSSTYPEGLGRAEWEHENASA
WTEAEGRDLFRVEGRALYIPRVTLAHAGNWTCNLYRGNTTVGQLGYLLEVTAMDYLGAELPPSTSRRATLAAVLA
LLLALAALALGLLARKVKARRRRNFPALDVTLETANPTPGKKV

Scyliorhinus canicula **(small-spotted catshark)**

CD4

Source: Whole genome assembly sScyCan1.1, GenBank NC_052161 (Chr. 16), reverse orientation, positions 86751677-86800007, prediction by hand based on comparison with *S. torazame CD4* cDNA sequence.

MCAQGRIYELVLFLTSLQHVSYSRPVTERDKIYATVGDTVTFLCQVNNPSQTPITKMSGGWKWHPSESDQSAKLIL
RYDYSNIPSRGNIDLVKRSRISSRRQSGNFSLTVSNVQPGDVGYFVCEFRFKSSEARAKIHLDVITVTSSSSKPSLEGD
NVELTCHAPAGKVSWNGPGDTQRDKNTVMLNAISLSHAGKWTCNVEFAKETLHTSYVLDVIGFTNPVERVIGVP
EGSSTQLPCTLNQFPSTRPVRGAWLRGDRELVSMNVSTNGWMWRSSWESRVRTPRFLNKINMSVVLTVNTLG
DGGDYICQLGLRNRNISRKLSVTVIQVSVSEPGMIIIGSNISLSCLISHHSPSTQIQWRHSNMSSKGGIAQGKDSLTI
NLIKVTKHEAGVWTCEIIQNNARLGAATLTLNVTEPVVSIFGKDNLILMIGTSASLLLLIVIFTLIGICLAKRARRRRRA
LRRLRHPLCREHSHQLSNQPLCNSNDYTSTERPLPPLPRYCPHQPRRGRSSQGKGNRLGPRGQYTA

LAG-3

Source: Modified from GenBank prediction XP_038678308

Sequence:

MASLRPWMALFLLSAAHAFNTRAFATERVIAAAGGMASLPCLDGDRVGRKAGYRKLALSWSFERTGMGKVPLL
MMGSDGVKRGLCETSGRTRVSGERTDAGDYSLTARDLRSTDAGVYSCEVRHGSVYLERAVELHVQVTTDAVGLP

LEGSKLNLLCEMAEPLTGARYSWHRGGNMVTPTGQRVWTGDSGRKLFSTLKPEDQGDWECHVTFEMLSVNA
HYQLKVLGFLSSPQDVPTLYARPDSSATLFLAMPEVPCQPQTCGWLRGQGQDAQPVGNGSDHARTERHPGSLS
LTLTPALASDRGFFTGYVNVSGHWIERIVRLELIEVMASQVGPVLVGSSLSINVSMGYAAGVDSVEWQHENGSVQ
AGRSFREEGGGLYIPQVTAAHAGNWTCTLYHGEIVGEISYLLEITALDYQAAEAASSRRITLVVALSLLLVFIVGASLII
FRKFMSRRRNFPALDVTLTTVEASVKKV

***Scyliorhinus torazame* (cloudy catshark)**

CD4

Source: Our cDNA analysis, GenBank LC770928

Sequence:

MCAQGRIYELVLFLTSLQHVSYSLPVTEKDKIYATVGDTVTFLCQMNSPSQTPMSKIMGGWKWRQSESDQPAKT
IFRYNYDNFRSKGNIDLVKRSRMSAKLFSGNFSLIVSSVQTGDAGYFDCDFSYMAVEARVKIHLDVVTVTSSSSKPS
LEGDSVELTCHAPAGKVSWNGPGHTQSNENTVMLSAISLLHAGKWTCNVEFTKKTLQTSYVLDVIGFTNPVERVI
GLPVGSSAHLPCTLNRFPSTRPVQGAWLRGVSELVSMNVSTKGWMWRSPQNTRIRTPRHLSESDMSVVLTVRT
LGDGGDYICQLHLQNRNISRKLSVTVIQVSVSEPGTISIGSNISLSCLISHQSPSTQIQWRHSNMSSKGGLAQGKNT
LTINLIKVTKHEAGVWICEISQNNARLGEATLILNVTEPVISIFGKDNLILMIGTSASLLFLIVIFTLIGNCLAKRARRRR
QALRRLRHPLCREHSHQLSNQPLCNSNDYTKTERPLPPLPRYCPHQPRRGRSSQGKGNRLGPRGQYIA

LAG-3

Source: Our cDNA analysis, GenBank LC770929

Sequence:

MAFLRHWMALFLLTAAHASNTRASATERVIAAAGDMASLPCLDRDRVGRKEGYRKLALIWSFERTGMGKVPLL
MMGSDGVVKRGLCETSRRTRVSGEKTDAGNYSLTARGLRSTDAGVYSCEVRHGSVHLERAVELHVVQVTTEAV
GLPLEGSKLNLLCEMVEPLKGARYSWSRGGNMAMPTGQRVWTGDSGRRLHFATLEPEDQGDWECHVTFEML
SISAHYELRVLGFLRSPRDVPTLYARPDSSATLFLALMPEVPCQPQSCGWLRGQGQDAQPVGNGSDRAQTERRP
GSLSLTLTPALASDRGFFTGYVNVSGHWIERIVRLELVEVVASQLGPVLVGSSLSINVSMGYAAGVDSVEWQHENG
SEGQVGRSFREEGRGLYIPQVTAAHAGNWTCTLYWHGELVGETSYLLEITALDYQAAEAAPSSRRITLVVVLSLLLV
FIVGASLIIFRKFTSQRRNFPALDVTLTSVEASVKKV

***Heterodontus zebra* (zebra bullhead shark)**

CD4

Source: GenBank GGGL01058619 (a TSA sequence)

Sequence:

MLAPGPLCGTLLIFLALLEHGVGTASSSVVEGDDIYSTEGATVTFQCTMNYREKKPITNYYGSWSWRASQGNGPP
QKIFQFLHGSVIQKLNTDFQSRIRISSLKHSGNFSLSLSNVRARDAGSFICNFQHQGYKAEATKHLHVIRVISNSSNP
ALEGDNITLNCLAALGQVKWRGPRVGDWTEKNLPLLGIKVEDRGSWVCSVELKKGFVEASYALDVVGFSSPSDQ
VIYIESQKTAILPCILNQFPSTQPVQGGWHLHDKELLSMNVSGSSWGWNKPQDHRLSLLGGKLAPTNLSVLFKAS
DLSDGGKYSCQLTVRSRSISRTVTVSVIQVKVNEPGTVMENSNVSLSCLVSDNSHLTEIRWHHGNGNRSPDTGGR
WKLTSDMIQLTAVSKHEAGTWVCEIYHDDVAKGRATYKLTVTDAPHRLFTTGTMIITIGTSAAAVAAILIATLIGIFLA
KRARRRRRAVRRLRHPLCREHSHQLSSQPLYNGNDYILTDRPLPPPPIRYCPHNQPRRGRPSQATSSRRAPKGSYV
A

LAG-3

Source: GenBank GGGL01678083 (a TSA sequence)

Sequence:

MPVLRGWIVALLALASVARATDENESLRETVLAPAGSTAILTCSEPTAPQFKGTYRKLALRWALETPGPGRASGLG
AWGSRYTLLMVGSDGVVKRGLLEISRRMRVPRERIERGDYSVEVRRVRPGDAGTYHCLVRHGTIRLKKVIRLHVV
QVSTDSPGVPLEGGTVSLLCNISEALPQARGSWSRAGAPVLTGERIWTDRDGMGLHIGKVQSVDRGDWECRVV
VDGFTVSATYNLKIFGFAGTEGEVPVTYASPGAVALLALRLAPARPPGPLGVGWLKDQEAGPLLDGGRWLTELRP
GALTLTLTPVLPSDRGLYTGYVNVTGHHIERTTRLELVEVTASQEGPVPRGSSIRLNFSTSYPAGLDVVEWHHENGS
AGQAGESFWEEGGSLYIPRVTPAHSGNWTCTFFRRGQPIGTVTYLLDVSALDYQAAEPQAASSRITLVVVLTLLLLL
IICITLIILRKWRSRGQNFPALDVTLVTATLPTKKL

Bichirs and Reedfish (Class: Actinopterygii [ray-finned fish]; Order: Polypteriformes)

***Polypterus senegalus* (gray bichir)**

CD4-1

Source: GenBank prediction XP_039619593

Sequence:

MHLDAMKVLVLISLSFSFRSCLSQSITTVYGPLGGSVVLICTKNGGHKTTLQWLFRKTEFGEEQQIVRQIPSGTITS
GQSELAKTSALYQSGCSLEIKKLEREYEGYFTYTLTDSSKTSPKLTEKTYRLRLFQVNSVPNMPVLESEAVTLHCNPLE
NDDLVPEISWKPPQGIQLETRDGLHVSNQGKSLMIASVLKKHRGDWICNLKYKEVVKQFTFKMTVIGLNSSPSKV
IYSLLHTSVLLPCFIDEGQQSFAMDQFVKSELTGASWKFYTNDKTIPYYGNVSSNTGNPEQSDSLNKSLSITTSLEN
RGTYICEVQFKRKTLTASVDLDLVQVVPDTTVPVMEGNELNLTCKVDKVVENLKLLWTPTKKNLNFQTNSFPGTA
TLKIDKVTNQHIGPWRCSLYVGETLMTSVEHKLKIVKPPVDVWLILLVCGSIILFAVLITVICCIIKRCQMMSQRRLK
QRKYCRCKHPKPKGFQRV

CD4-2

Source: GenBank prediction XP_039619594

Sequence:

MWNRKYLLTIFGLLTLSCDILGTATVVYESIGGKATFACKKTQNDDKWTYKSNDFATEDIVIRSSKRMLELRGHLPM
VKRARIVQDFSLEIKDVESSDEGIYKCSSEGKEYKLCIVSVHADPQGPGVQFTPLKIKCAICENTPRIWQHPHKINIM
NSNNYIINKEEGTIEIKSLSMEDAGTWTCKITNFHIEYKLQIIGFTGNSSLTTFCKKGDTAYLPCQLHSLPSVPDFGNLI
ITGGGWKKKAEQDSMDQLLFLQFMNRELSWNLSVVNKRLTFHSENIGRNLSVTIKNVQLKDAGEYICEVQFNTR
ILKKTVKILVEDVITEVTHSTGVNKSGHKKMSLWMFILIGVVAFVLIVLLIIVTILIIRHQRLRAQAKRRAYQRQALTPK
QYCQCHRSRNRNGYSRQPVKEKLLDEEFY

LAG-3

Source: GenBank prediction XP_039619595

Sequence:

MFHILCCLFGTLHLLHVCEASTDYDVFYEKGSTAILPCYRLPSKSTVNRMAVVQWLKVNEDSSDKTIWRVDKSGLE
YWSNNILKRAQAHQAHFHKGDYSLSIDHLGEDDAGQYKCRVKYGHQEFKVTVNLHILQAVPTEKSKPLEGRSWN
MKCIISDKPETVTVRWFHNGKPVQESDRISIKNSDATLTIHNLEPTESGNWTCQVVSNNQTGSASALLQVHGFAE
PTSKTTTVYAAIGAPVYLPCILSSGVQPMKTGWQWKPSGSIQIQELKSRQHSISIAESKDQSNVSLFLPAVEFKKAG
EYICFAEMDTLRFHRIVKLVTAQVIVKVSHSAKDGSLATFSITLSEESGVERYEWVKIKKDSNYTNSSDHTLAPEKLG
PYFGKTVSISLLSEEFAGEWVCNLYSKEALLGQIPVHLLLTGMLQGSKSESSKNVPMILIFICIFIGFLFITLIVYRYRRR
RARNALFPALESVEKSIPSNKKLSEKNCSQKCKGGLN

***Erpetoichthys calabaricus* (Reedfish)**

CD4-1

Source: GenBank prediction XP_028665852

Sequence:
MHLDAMKGLVLISLSFSFQSCLCQTVYGPVGGSVVLSCTKNGGHKTSLQWIFKKTTDGKEEQMVRQMSSGTITS
GQSELAKRTALYQSGCSLEIKKLQREDEGYFTYTLTDSSIKPSKSIEKTFRLRLFQVNSVPNMPVLESETVTLHCNTLG
NDDLLPEISWKPPQGIQLKTQDGPHVSNGGKSLMISSVLKKHRGDWICNLKYEDVVKQFTFKMIVIGLNSSPSKV
IYSLLHTPILLPCFIDAGQQTLAMDLLDKSGPTGASWKFSTNDKTIPYYLNVSNNIGNHGQSDSLNKSLAITTSLESR
GTYICEVQFQRRTLTASVDLDLVQVVPDTTVPVMEGNELNLTCKVDKVVENLKLVWTHTKKYLNFQTHSFSGTAIL
KIDKVTNQHIGPWRCSLYVGEKLMTSVEHKLKIVKPPVDVWLILLVCGSIILFAVLITVICCIIKRCQMMSQRRLKQR
KYCRCKHPKPKGFQRV

CD4-2

Source: GenBank prediction XP_028665305

Sequence:

MWNSKYLLTIFGLLTLSCDILETATVVYESTGGKATFDCKKNSNDPKWTYKSNESAKEDVVIRSSKNMRELKGTLP
MCKRASLVKDFSLEIKGIESSDEGIYKCHSEEKEYKLLIVSVHADPQGPGIQFTPLTIKCIVSENTPRTWQHPNKINIM
NSNNYMINEEKGTIEIKSLSMEDAGTWTCNINNFHREYKLQIIGFTENSSLTTVYKKGDTAYLPCQLHSLPSVLDFG
NLIITGGGWKKKTEQDGMDQLLFLQFNNRELSWNSPEVNKRLTFRSENMGRNLSVIIKNVQLKDAGEYICEVQF
NTRILKKRVNILVEDVITEVTDSTGASKSGHKKMSLWMFILIGVVAFVLIVLLIIVTILIIRHQRLRAQAKRRAHQKHA
LTPKQYCQCHRSRNRNGYSRQPIKENLLDQEFY

LAG-3

Source: GenBank prediction XP_028665848

Sequence:

MFHILCCLFGTLHLLHVCEASTDYDVFYEKGSTAILPCYRLPSKSTVNRMAVVQWLKVNEDSSDKTIWRVDKSGLE
YWSNNILKRANAHQAHFHKGNYSLTIDHLGEDDAGQYRCRIKYGHQEFKVTVNLHVLQAVPTEKSKPLEGRSWN
MKCKISDKPETVTVRWFHNGKPVQESDKISIKNSDATLTIHNLEPTDSGNWTCQVVSNNQRGSASALLQVHGFA
EPTSKTTTVYAVIGAPVYLPCILSSGVQPMKTGWQWKPSGSIQIQELKSRQHSVSITESKGQSNVSLFLPAVELKQA
GEYICFAEVDTLRFHRIVKLVTAQVIVKVSHSAKEGRLATFSITLSEESGVERYEWVKIKKDSNYTNSSDHTLAPEKLG
PYFGKTVSISLLSEEFAGEWVCNLYSKEALLGQIPVHLLLTGMLQGSKSESSKNVPIILIFICLFIGFLFMTLIVYRNRRR
RARNALFPALESVETSTPSNKKLSEKNCSQKCKGGLN

Sturgeons and Paddlefishes (Class: Actinopterygii [ray-finned fishes]; Order: Acipenseriformes)

Acipenser ruthenus **(sterlet sturgeon)**

CD4-1

Source: Our cDNA analysis, GenBank LC745920

Sequence:

MCLYDRKAVFVLLWVWCAVALDAMAASRNVFGVVGEKVELPCGTLAHSKFSVVWSFGKQEGSQPQKFIKQSFP
GSQRKDSSDIAKRSTLSNQENSLIINPAKAEDEGYFHCLLSGDTGSKENKYRLRLLKVHADPDGPVLSSQSVTLTCD
VKDISDLAIKTSWHDPRGTQMMASRKQENKLVLNNLTAKVSGDWKFRVEYNNKTVEATYRMVVIGFVDPQPVI
RYTRPRASVLLPCLIDKVMGTVKWDHVIRAGLRGVEWSFIPDTGKERKILSMNTTGADIPWVIPGQNHKFRYSTS
ERNSMNFSLLISDVRPVDEGEYRCHTQFQGNNLTSVVHLEILKVISEPEGPIVEGTGVNLSCVVRKEAAEFKAEWS
AARGPKQEIQTHAVAGGLALRVDSLTKEQAGPWKCSVFIKGHLQTSLEYTITTARPPVDVWLVLLVGGAVLLCTLLV
LLIYCFVRRRPANPMHRRPRRGRTKYCKCKHPQPKAFYRA

CD4-2

Source: Our cDNA analysis, GenBank LC745921

Sequence:

MGNEMKSVMRVWLKMFILGFLQAASYGNIYVSKKSVVTLPCDVKPQTQVAWSYKKDVSSAYTNIVKNVGRNTL
FGTTPMKSKSRILSSSALQIQDVETGFAGFYQCEISGIKKEYNLLVVSVVPEHPGPYLQSSSLKLDCLVSGSSGESPV
WLQPGSEEVWSSSSTLEIKELTMRHSGTWRCRVGSFKLEQKVEVLGIQADPSSGMLSVKNGGSVFFPCELSSSLT
ELTELTESERAIWSRVTQESPAEQEILEFNKPKKRPLLSSSKGNNSISLSKVSPVDAGRYTCLVQINGANMSKSFQLN
VLSVESSGGNVVCCLHGNASGFQMEWSDAEGQTRDLQMQQGTSKTTLTLPMAQMNSTAWKCNLYLGSKLQT
SIDYTPGTADDRSSSNVGFRGWVAAVLKMELWVLISICVGGVILIALVGVVIGLLVRRRHLKRLTRRRRLQKQPLTP
RQYCQCQRKRTINGSRRMNSYQEEDRDY

CD4-2f

(this sequence is very similar to CD4-2 but the cytoplasmic tail CxC motif appears to be inactivated by the replacement of the second cysteine for a phenylalanine, and also the cytoplasmic tail region where the other CD4-2 sequences appear to have an amphipathic α-helix that may help bind LCK is considerably shorter than in other CD4-2 sequences; the TSA transcript report GKEF01826349 supports that this gene is actively transcribed. Furthermore, the TSA transcript reports GGQL01014569 and GGQL01014566, respectively, indicate that also in the related species *Acipenser sturio* both *CD4-2* and *CD4-2f* are present)

Source: GenBank prediction XP_034760430

Sequence:

MGNEMKSVMRVWLQMFILGFLQAASCENIYVSKKSVVTLPCDVKPQTHVAWSYKKDASSAYTNIVKTIGRNTLF
GTTPMKSKSRILSSAALQIQDVETDFAGFYQCEISGIKKEYNLLVVSVVPEHPGPYLQSSSLKLDCLVSGSSGERPV
WLQPGSEEVWSSSSTLEIEELTMRHSGTWRCRVGSFKLEQKVEVLGIQADPSSGMLSVKNGGSVFFPCELSSSLT
EPHYQSERAIWSRVTQESPAEQKILQLNKPKKRPLLLSSKGNNSISLSKVSPVDAGRYTCLVQINGAKMSKSFQLNV
LSVESSGGNVVCCLHGNASGFQMEWSDAEGQARDLQMQQGESKTTLTLPMAQMNSTAWKCNLYLGSKLQTS
IDYTPGTAGGNSSSSVVSGGWVVAPLKMELWVLLSVCVGGVILIALVGVVIGLLVRRRHLKRLAQTPRQYCQFQS
KGTNNGSRRMNSYQEEDSDY

LAG-3

Source: Our cDNA analysis, GenBank LC745922, in combination with GenBank prediction XP_033897857

Sequence:

MLCLFTLLIGIALFCDVSRSSAEEHIFAGVGSRVLLPCLKTPLPHGVNRGTTVHWFKIFSKSAERSVWRSEKSGLEF
WSSLTNKRAKAHQPRFHSGDFSLAVEDTRMSDAGRYRCAVSYDRENFQRLIHLHVMQVSPVTAGPFIEGSSLTLR
FSASDWPQGARVSWLQNGIVLQSSRKHQIRERSLDIKDLDREDSGNWTCQVSYQGRTARVSYTLEVLGISSPPSS
GSVVYGSLGLPATLPCVFSAGLTPSHTGWQWRADSSVVPVSRAAPSGRDASLLIPSVEFRHGGVYTCSGSVHGKR
IERSVRLVVASVSVKPSGLVSEGKSVSLTCTLSDYSQVDRFEWTKVTANSNQTDPTDPPKQASLLTSKGAKLGGLNS
DRTFLIPRVSEQHAGEWVCSLYSKEILVGQIPFQLHVTGQLQGSALAQSSNKVAAVTLLSFLFVVLLLIALLMYRYQR
KRARGNLHFPGLEAIRTEQSKKSREEKKSAEALKEDEP

***Polyodon spathula* (Mississippi paddlefish)**

CD4-1

Source: GenBank prediction XP_041125128

Sequence:

MCLYERKTVFVSLWVWCTVALDAVAASENMFGVVDEKVEIPCETLADRKFNVVWSFGKQENIQSQNIIKQFHIG
GVRKDLSDIAKRSTLSNQENSLIISPAKADDEGYFKCELSWDTGRKQHKYRLRLLKVRADPSGPVFSSQNVTLTCDV
GDTSGLTINTSWLDPKGNQVTDSRKQENKLVLNKLTAKNSGDWKFRVEYNNKIVEATYRIVVIGFVDPQPVIRYTR
PRASVLLPCLIDQVMGTVKWDQVISAGLRGVEWSFIPDIAEERKILSLNMTETGVLKTILVQNYRFSSSISERNPM
NFSLLISEVRPADEGEYRCQVQFQGQNLMAVVHLEILKVISEPKGPIVERTGVNLSCVVRKEATGFRVEWSAARGP
TQEVHTHAVAGGLALRVDSLTKEKAGPWKCSVFIKEDLQTSLEYTIITARPPVDVWLVLLVGGTVLLCALLVLLIYCF
VRRRPANPMHRKPRRGRTKYCKCKHPQPKAFYRA

CD4-2

Source: Modified from GenBank prediction XP_041125461

Sequence:

MGNKVKSVMRVWLKMFIVGFLQAASRENIYSSEKSVVTLPCDGKSQAQVTWSYKKDASSAYSIIVKTLGSKTLFG
STPVKLKARLQSATLLIQDVETDFAGFYQCEISGIKKKEYNLQVVSVVPERPGPYLQSSSLKLNCVVTGSSGDVPVW
LQPGSEEVWSSNTTLEIKELTTEHSGIWRCRVESFKLEQRVEVLGIKADPSSGTLSVKKGDSVFFPCELSSSLTEPQY
QSEGARWSRVTQEYPIEQEILLLDGSGKILELNKLQNRMLFSPSKGNHSISLSKVRPADAGKYTCQVQISGTVMKK
FFQLNVLSVESSGVNGVCSLHGNASGFLMEWRDEDGLKRDSPMQEVDNKTTLILSMAQRNSTKLKCNLYLGSKL
QTSIDYTPGTAGDSSSNNVRSRGWVASVLKMELWVLISICVGGVILICLVGIVIGLLVRRRRLKRLARRRRSEKIPLTP
QQYCQCQRKRTNNGSRRLNSYQEEDN

LAG-3

Source: GenBank prediction XP_041123852

Sequence:

MLRFFILLIGIALFCDVSRSSAEEHVFAGVGSRALLPCFKTPLPHGVNRGTTVHWFKIFSKSVERTVWRSEKSGLEF
WSSLTSKRMKAHQPRFHSGDFSLAVEDMRMSDAGRYRCAVNYERKNFQRFLHLHVMQVSPATAGPFIEGSSLT
LQCSASDWPQGAKVSWLQNGIVLQSSRKQQIRERSLDIKDLDREDSGNWTCQVSYLGRTARVSYTLEVLGISSPS
SSGSMVYGSLGLPATLPCVFSAGLSPSHTGWQRKADSSTVPGSRAAPSGRNASLLIPSVEFSHGGVYTCSGSVNG
KRIERSVRLVVAAVSVKPSGLVSEGKSVSMTCTLSDYSQVDRFEWTKVTTDSNQTDPTDAPEQAPLLTSNQAKLG
GLNFDRTFLIPRVSEQHAGEWICSLYSKGSLVGQIPFQLHITGQLQGSAPAQSSNKVAAVTLLSFLFVVLLLIALLMY
RYQRKRARRNLHFPGLEAIQTEQSKKSRREKKSEEALKEDGP

Gars (Class: Actinopterygii [ray-finned fishes]; Order: Lepisosteiformes)

Lepisosteus oculatus **(spotted gar)**

CD4-1

Source: Modification of GenBank prediction XP_015193339 with the help of the TSA sequence in GenBank GFIM01016821

Sequence:

MNFYKKLFFFVVMMSFQTACVGQEVVVYGVAKGSVTLPHNKWGGQRVSVLWTFGTSASSIATEVYYQSFSGTP
RIGPKLAKRLSLSPGTPSLTIRELQDTDFGYYKLQLTEYSQNTHTIYRLYKIEVSSPQPSTLLATEDFSLKCVVDSSSART
VTTWFSPTDKELTPGGRISYSETESQLTVRNASRSDTGNWGCVVQYNQKSTTATHGITVVDLNAHSQVIYSSSISK
SVLLPCSLSPGLTFKDVKNVGLQSGEWIFTPKTAKHPFTLTSLNLQEEKPTWSIPQNPKLNVSVIRDDNRNFSLIINQ
PPLENAGLYRCTLKFKNKLELQSTVELRLLRVVKDVNTTLYEGQDVNLTCTVDSILDPSFNVTWAPPRLSKAQLQEV
RLTEGSRLTIRSTSEGDSGKWKCSLEKDGNTVISVALKLKIEKVPVDVWLVVLGCAAIFIFTCLIVIIVRLIRRYQQNRG
FRRAKRRRTRYCRCEHPKPRGFYHT

CD4-2

Source: GenBank prediction XP_015193374

Sequence:

MVRREDCLLLLWIAAFAHPAQGEVFYVRENSRSLDLPCSSSHLKSTSWKYSSNGNDYRLIFTIDKSGSQRKGHQFP
AQGRAKPGGRGLQISPVKKNDSGWYMCEQDQKQLVTHRLLIIEVFTKPEGPFLQNTNVTMHCQVGGRTSEKLK
WLSPNGAEEGEGTVEISKISPAHTGNWKCEITDVKKYVETLNIDVLGMNPQPLSKQTAKQGDNVFLPCLLSSPLSY
TFRSLNFIRGGWMKDDKPLLDLYVQIGTHMSWNATMFKEIEYPKVNDLKTNLSISLIKVKPQQSGIYNCYLRFKEG
TITSTIVLKVEGAPGGTGDTSHSSSAESVSVFGLNMNLWVLVSIAIGSVVLIILIIVAIILLLRKKRLKKRMRKRRSQRQ
PLTARDYCQCNRNNVSRNVGRQRENGNSLKQQLH

LAG-3

Source: GenBank prediction XP_015193364

Sequence:

MRLIALVVWTILWASVTQGEMIEVFAERGSTAVLPCLSHAPLKNPLTVQWTKRNNMMESTVWRMERSGMEF
WGSPGPQRSQCPHSGFQDGDFRLIIQEVRESDAGEYKCTARGSRGIVQKTVILRIIIVLVSQSLPLEGSTVSLNCSI
QPQPQKATVSWLNNGTVVGNSPKTEVTKKGWTLTVKRLTLADATDWSCVVHTARQTGRGTQRLTVRGIASPA
SDGTMYGAVGSSLTLPCLFSEGLIPQQKGWERETRGAVPQSITPSPPSPSSSPSLDASLRLSAVEERDGGVYTCFG
LVEDRRIERRLRLVTAKVGVTAPPKAGQPLTLTCDLSDATGVDRYEWFRVTSDANGTEVTTPLGTSSEKSFRLAG
GSGELSGELVCQFHGKEGALGNVSYHFHTLGRLEATTKGGSSSQVAIITTFSFLVLVLILILLQMYKNHRRRKMILP
YPALETVIHSSSRAQERTERKKTTDPEPTTN

Teleost fish (Class: Actinopterygii [ray-finned fishes]; Infraclass: Teleostei)

Danio rerio **(zebrafish)**

Zebrafish also has a CD4-2.2 gene (GenBank HE983357), which is not shown here but is a noncanonical recent derivate of a canonical CD4-2 gene such as is zebrafish CD4-2.1.

CD4-1

Source: GenBank NP_001128568

Sequence:

MLGLILIPLFITVLKAQESHEVIYAQVGGTVTLPREKIERKYSNIKTQDIYVNWFLESTLTINRNPQSSSSKGTNARVS
LSADFSLQISPVEESDFVIWRCEQHVLARNYKKTYKLHKVSIPKVPALLVGGRLFLKYVKDVSSVNPSVTWISPKNE
GCQEHKNAKDTVLVPSVSTCHNGVWTCQLKYENKKTEATTTVSVIDLAPSPADPIYTSISQSSTVSIPCALSSAIPW
SVLNETLQGGSWSFTPLSEPRSPLSLLTLNVGSVVRWDLANGANFTDGKRVITNHNLSIQNLPVKETIRGVYKCSL
KFNTKTISREVKVEVLKVSPSGSLKVFEGTRLNVTCSLGHMNTAGLEVKWTCASNCPPFNHKSPPHLSVLSFPKIR
MQDKGLVKCELWKNSQKLTSAQLYLRVEKAPVDIWLCVAIGSGVVVFILLVAFAIIYIRRHKQMMMYRRRKTRFCC
CNNNKQPKGFYKT

CD4-2.1

Source: GenBank NP_001352990

Sequence:

MIFFLLLALSVSSGVCDVLYKEAGKEVILQCGAPPNSDIEWRLNNIRLISITGKSGLRRKGSGHSVDKVNLYGDTLKI
PRLEPRDSGVYSCAQSGKQYTLHVVSVFVKPGPVLIQSSDVELHCNIEGDPNTEVEWLRPPNDQVHDAKHQKIN
LKSVTSSDEGKWTCKVEDLKLSVTLTVVANHQINNVEVSERDDIELPCFLPRPVSQSVLGGKWKADHLPTVPFPT
LKNTADEGLHWDGVNSSVVKYNIERISTIFNVTLKKVQSIFAGKFVCEVEFEHGGKLTAVTNLTVKSWTDRNDGK
TGKNSKPGLAGEIFRKSMFGVELWIWIAVGAFSVVLVPLIIGIVCMQQKNKQKKRRVRKLRSMRQPLTAKDYCQ
CNRSDREVVLCQRERPLPAPRQQRNLRTAGLNHAYEHA

LAG-3

Source: GenBank GFIL01014211 (a TSA sequence)

Sequence:

MTLTGLVMVLGLALVFEGGECRHHEVFVAEGSVAVLPCMDESSSVSHPNAVYWSKIVGNSQKTVWRREKSGLEF
RPLRDLPRAKCPAPNFGKGDYSLHITETRLADGGQYICEVEGKTKMQKVIMLRVIRASISPSIVFEGLRIEAMCHVK
PETSDISIKWKQNGEFINSAIISTVSQRDAGKWTCQVSYNNMKVEATTTLQVRGISSPQNDSSVFYGSVGSTIFLPC
VFTDGLIPNTVKWLRASAATNSPVTLPHSAFNSSSGFIKRVEVGDEGTYMCSGVMEGLNGRSIKLQRTMKLVVAR
VLSSSSDPMTLMCNLSDSSQITSYEWLRVNYSPNDTQTLTTVQRTKNPSIRVTEKDAGEWMCRYYGNQGLLGN

VTSHFYTMGALKSGNSSSGNKTAMVLGLGFLFMVIFLILFQMYRNYRRKQKIHLYPAMENIVHQTITEREWKERS
REKRAEACIGEPKSVCV

***<u>Ictalurus punctatus</u>* <u>(channel catfish)</u>**

CD4-1

Source: GenBank NP_001187155

Sequence:

MSFLLGLLLLLAPCHSAADEPKGIFAQFGNSVTLPRRIWGIEGKIHVNWYFQDNLLISRNPTLSASKTVHNRFSLSS
DSSLIISNVEKSDFGIFKCEQHHLVETITDTYKLYEVMMSTPPPLLVGASLDLSCEIESEGFKLVHEIKWFGPDNTLYV
GSSSSNQRTLRVTKVSSIHSGKWTCAVRYGASITLKARTDVIIVDLASSSPDPIYTSDSSINFLIPCSLSSKIPWSTVNA
TGVTGGSWHFTPFKSSESSLPLLKLQLNPSPAWKFPSGTHTLLMETDLKNHELGVKISKVSINERGNYTCSLEFGSR
TLSRSVQVEVLQVISSEGKVIYEGNTVNLTCTLGHHMTPDLEVNWIPPYGSSLSKLSPPYTTMLSIPGVSVKDSGR
WTCQLKKNATLLTSATISLKIEKAPVNIWLVVAIIGGLLVFILIAVITVFIIRRHRQMMRYRCRKGRVCCCKNPKPKGF
YKT

CD4-2

Source: GenBank NP_001187156

Sequence:

MFRSKNILWITFAFCLTSGSCTDIFQQSGSDAKMDCSGGDPKKAIEWKRGNVLLIGKAPSGTVRRGIMETTSRARI
DGTTLKITQLKTSDNGVYTCNSYTYKLYVVSASANPSSVLYSSETTLSCDVAGDFKGTFQWLESGSKPYSQSKEVTV
KNVTLDTARIWTCLIKNEKSKEIIRLDVNIGVVGPLNTPREVKTHEGGSAVLPCFLPTKSQLPITGGSWKRESHSDIR
FPVLMRKQNAVQWNSTDVSIDKVTFTEQEVMTNFSVTLKKVKVADAGVYVCSLKFENGKALTSSLNLTVSKRDG
DDPDMDSRGSTVTKNNMWNKRVWGMQLWVWIAVSASSFVLIGLVVIILLIHCRNKRMKKKMMKLKSMRQP
HTSRNYCKCDRPVSQAGTGKRGRPPPLPRHQYSSLNE

LAG-3

Source: GenBank prediction XP_017328657

Sequence:

MQVVEHCRQLSKKKSYNLSTEMAQVYMKVDVLFLLATAMAISRSDYVRASEVFVVSGSVAVLPCVTSSPTKQSSA
VTWKRIFEREERTVWRRDKSGLEFRPVGQAPQAHCPYPNFGNTNYSLHIEGTREEDGGTYRCEVEGQKFQDVKL
HVIKVSFTPAEVFEGDRLTVNCNVTPKIKSVIWMWELNGSPITSNSWKQTYTVDKVSQKDAGTWSCLMRNKET
QVWSQEVKASTLLQVKGILVPRDNSEVLYAELGSSVTLPCIFSHEFLSNSSSWKRLSKTSNLSAMLPSFFIASSKPGV
SGCSSVLRDRSAYIERVQDGDEGTYKCSGQVTGDNNKRVTVERNIQLVTTQVVSSDRNGKTTLTCRISNPTQVTSY
EWIHVEYGVNDTQTFTSVQKSTSKVLSIPKEKQLGEWVCRFYNQQQLLGNATYHLQMMSGLEGLEKSTSGNKV
VTIIGLCFLCLLLVLIVLQLYKNHRRRKMILQYPAMETIVHLAANEREFRERVKVREKTQNGACGEDLKSVFV

***Oncorhynchus mykiss* (rainbow trout)**

Because of a whole-genome duplication in early salmonds, rainbow tout has two similar CD4-2 genes, CD4-2a and CD4-2b.

CD4-1

Source: GenBank AAY42068

Sequence:

MKCVSGFLSIIIALFISSTGAEDVVVYGQVGETVTLPRSKWGSERVLVQWFFGIDTQPLISRNSHGRETIDPEWKDR
LSLSKTDFSLIINNIRLEDFKSFKCELKDFMPQTSTSVTFRLFRVSVQPVSPLLAGKNLNLKCDIEEIFKGTQRRWLSP
QKQDLNEDKRAQIRNDGSLTVMSVTDQDHGEWTCVVTYQGREAYANTHVTVIDLSPAHPQPIYTSVSSLSLLHLP
CFfSIPPPLSWSDSQEKSIQGGRWTFTPSPAAGSLTGVVQTLANLSLGPPLAWVVNQKRELDVSALQRTNLNLSLS
KKGVTEGDRGEYTCAVEFQRGDTLKRSMRVEVLQVFSSPAPVAFVGQEVNLTCTLGHPLTSDLKVKWIPPRQSSLL
ALGSAPDSAHLTIPEARDINGGRWRCELWRNKTKLTSVEITLKIERVPMDVWLLVTICgAAVIFVLLLILTVILNRRHR
QRVTMPRRGKRRICRCKDPQPKGFYRN

CD4-2a

Source: GenBank NP_001118012

Sequence:

MKTLSWFVFALCILHVVGEVIYKRIGLPVNIDCGVKTSNKDMEWSHKAVGGSKSVLIVDYFGKNGKERKGNAPM
VERAKVRRDRLEISALNDGDAGLYICKVDGKDMDHRLDIVTVKVHPSNELNEGNNAILECQVTGVDPLPSVEWV
SPGGKVEGAPGRPGSRNVSFSSVALSDTGEWTCQITQDEKTHKETQTINVRSLLPNEGQDDGQGHSGPNSDVN
TVTTCHHCTKGSQQPVEWVPMLGLSLWVWVAVGAGCLVGVLLLVTIVLLHRRNKIMKRRDRKMKNIRVPLKSN
DYCQCNRTLEGPPRRTQREKPSAGPRQQR

CD4-2b

Source: GenBank prediction XP_021417861

Sequence:

MKTLSWFVFALCILHVVGEVIYKRIGLPVNIDCGVKTSNKDVEWRHKAVGESESVSIMGIKGKTGSQRKGNAPM
VERAKIRGDKLEISALQGGDAGLYICMVDGKKMDHRLDIVTVKVHPSNELNEGNNAILECQVTGVDPLPSVEWL
SPGGMAAGAPVRPGFGNVSFSSVALSDTGEWTCQITQNEKIHKETQTINVKSLLTAKPLLPRKGPVDEQGHSGPN
SDVKTVTACDHCTKGVEQSVEGVSMLGLSLWVWVAVGAGCLVVVLLLVTIVLLYHRNKRIKRRARKVKNNREPLK
SNDYCQCNRTLVGPPRRTQREKPLAVPLKQR

LAG-3

Source: GenBank NP_001182204

Sequence:

MWQFLLLLGTSLLVTGGRCQCLSEYTEMFAEAGSQAVLPCVCRPPSTSAAVVLWSKDLEGTVWRKGKSGLEHW
GIGAAQRVRCPHSEVGSGDYSLYIKEVREEDSGNYTCMVQDGEKILSKRILLRVIKVSISPPAPVEGNKMTITCSVTP
WPQEATVSWMLNKKRVYPESADYVLSKNQASVLESKASAGMMGNWSCVVHKGRKQGKATTALTVRGIVNPSS
ASAKVYAEVGSAVTLPCVFSTGLTPSDTAWERLDTSGSVLPLPPSFNLSSLLSLPPWDRSVGVGQVGQGDGGRYR
CSGTVEGQRVAREMQLVTAQVLSNSPSTQKAPVSLTCHLSDASEVTEYEWVRVTYDLNGTQSESSVQKGRVLGIN
KVSDRNSGEWACRFHGKEGALGSVTYHLHLMSGLMGDNETGSSSNVAMVAGLGFFLLVLLLVLVQMYRNYRRR
KLILQYPALETIVHTIANEREDRERNRVKEDEISK

***Takifugu rubripes* (fugu)**

CD4-1

Source: GenBank NP_001072091

Sequence:

MEPLPSGLLLLTALLSASRAEELIYAQVGQTVTLKPPENYKTPTYYLSWHFGELELAWTNHMSGNKVIKHENWDT
ALSDNSLVVKEIRQNQFGIYKCNVNEKIWTYKVLRLKVSAEPPSLVLSGRTVTLVCDAEPPNSLQKPGIHWLNPQG
EKITQATHSVQVSSRHSGRWTCVVTLDRKEATAQISVTVVDLYSPPMAYTSTSSPLAVPCSVPKVSWEQIKSLGLRE
GHWQFFPRSKSNLVSADAQRLFTLSLEEPVSWKANQTRGLTPVSDFKTPNLSLGRTLGRANDRGDYVCTLKFESG
PPLSTTVRVNVLEIAASPGTVLISGQQLNLTCGLGVPLTSDLHLKWISPERATIRSGQLTIPAVGAGNSGKWRCELW
RNDTRLTSAVITLKIEPKLSVWMLVIICSVAVIVLLLLLLGFILCRRRRARVRHVRHQLCQCKNPKPKGFYRT

CD4-2

Source: GenBank prediction XP_011604216

Sequence:

MKRTLWLGFAVFFSCALCADGYVVVKSPGEKVNLPCGADQSGGTVIWKHEHSVVIQVDKNGFPRKGQGDLGR
RATVRRMALEITGVKEADAGMFTCTVDRNSEQHFLFVVTVSATPSAVLQLGSSAALHCQVKGLPPPSAPQWRKP
DGSPHPGSEVAELNPVARSDEGAWNCTFSHGGLTYGKSLDIRVTGAATTPAKPVPSDKGKEAPTCNDCVTNRPSL
PLRLSWWMWAVIAVGCLILVVLMAFIVYLCKRIQRKKRKLRRMENSRQLLMPKQYCQCNRPTAVPKMQQRRQ
RQKPSAPPLQPVLVQ

LAG-3

Source: Modification of GenBank prediction XP_029694565

Sequence:

MLSEFFIFGLVMTSAITGGECEVTEVFAQEGSEAFLSCKCDPSSPNPASVIWTKHDKGTVWRKTQSGLQFWGTS
WLHKKTPRVQCPHYRFERRDYSLQINSVKLEDAGLFSCRVGTADGVIKHQVMLRIIQVSISPSAPIWDSTFSISCDV
TPPAEGATVQWTLNNISSIEAIKTLQVAPTKRSLVSGRASARLEGNWTCVVGYKGEVGRASVTLAMKGIIQPPKDD

TKVYAALGSAATLPCVFSPGLIPSSSGWEKLESGFPFKAATSPLPASFSQPTPSSQPSVDKSAILTEVRSEDEGTYRCS
GTVEGRQLTRNLHLVVAKVVNLANKAGSVTMSCHLSDTSEVTRYEWVHQDFDQSGNLTVVSIHKGKDLTVAKKG
DDKGEWTCRFYGKQGILGNVTHHMAVMSSLGGQNSGVSHKAAAAGVLSILLIVLLLVLVQMCRNHRRRKRILQY
PTLETIVHRISNEREEREKNREKK

Coelacanths (Class: Sarcopterygii [lobe-finned fishes plus tetrapods]; Subclass: Coelacanthimorpha)

Because of scarcity of data , we could not find CD4 and LAG-3 in the same coelacanth species

***Latimeria menadoensis* (Menado coelacanth)**

CD4

Source: GenBank KC677707

Sequence:
MKAGSLLLWLLLALLLVCEPSSAEPTEMLSAVGQSVKLLCTTTPKEKTQLSWKCNKVTQNKMDSILSFRPPSGGL
FKGPCYVDRYKLQDNYEKGTYNLEISPVKFDDSGYYECSWGGVIKETYRLVTFQMSSQPTGTLVQGDNVTLKCE
VSGQQSSVQREWKKGGETIQNDPRYKLNKDKSHLEISDLKPSDSTSWKCIVTMGEKKVEETYKLNVLGFEEEDK
DGDVRIATRDNVTVTLPCRLSFPLRKFWSDFPVESSSWHKVPEEEASLLGTYDNNGFTTKFKRYFLAQKNAEKDL
NICIGSVRFEDGGRYQCSVKFNGGKDLKKLIHLVVLNASVTPGILVQEDSEVTLSCRVSHHGTSTKLQWISPNETIY
SANSDSEKLEVHITKMTSNDFGIWICKWYEESKLETRINITLKEDRKNSICIGEFCTSKWILVGGSTAGCVVIIFLLFLI
PCLLKKRQRRQRRVKRPVKGRYCQCERGRRARQEAASRKASQNYNSYEPFQ

***Latimeria chalumnae* (West Indian Ocean coelacanth)**

LAG-3

Source: GenBank prediction XP_014349213

Sequence:

MLQDLILLFLVCTISKVSCSDMEVFATSESGGILPCHWLSSKAWQHKYTKTYVRWEKNQNGVSSTVLWTKNNGI
IMMGNALSRRALINEPHFKDGDFSMRIKPMLPEDAGVYTCTVQLGVFQKQCRVKLHVMQVTSSQDSPVLEND
SVRLTCDLTDEIDLGTVAWYHKGSRIYSTTPRCTLQRDGRELTVRNLTVHDSGTWKCELKHKNKTAMATHDLHIL
GFSNSTVQTHSIYAGVGSVAMLPCTLNMKPEGIQGRFYTGWSHKGESDTVAEEILLTNSRAVLEQAGKGERSHL
VFQADGSENLTLTISPMREADRGIYTCFVEIGGKRIEKSIRLAVLTIVSSGMGMMREGATIQLTCGSSELSGGERFE
WHQLDPTNTSQSRKTYWGQTLVLPAVSGTEAGKWVCTVYQDGIEVGHVEYDLKVAESLYGTRANDSPWKTTF
GVIAALLILLLLALLAAFLRQRKRREQRFPALESSRNAAISAMKKVFGEKSVEERKDEECL

Lungfishes (Class: Sarcopterygii [lobe-finned fishes plus tetrapods]; Subclass: Dipnoi)

Protopterus annectens **(West African Lungfish)**

CD4

Source: GenBank prediction XP_043935549

Sequence:

MGIEWFLMCILLWSTFGMVHIGAAQKQVFATEGKDALLPCTGPGRTKVSFQWLYETRSVVQIHYTGMPRYGSG
YSNNRITTFDYNKGNYSIKLSKLTMPDGGTYKCTSYSSELDVIKLHVIKVTSPDGEHYLHLTDVKLKCDINPPISENLIT
WLNPKGESVSSSSRYHVSDGDKTLDVRNIQIQESGQWRCRVKDAEAYYTVQVLGISDRSQSVMVYTSVNQSAIL
PLELTQDIKNSKFGNFNLKKGELLRLKEEGGRSTALALLNNSQQKLFWEETDKIQNEGSGHSLSIRLVSASFQDAGR
YRFHLTFDKGTLQNDVHLVVLQVTASPEGKIAEKDTVNLTCQVSNNTPPLKLVWKNMNDTSKKVEGQEGVRYLTI
SLTEKTQDQFDVWTCNLMENSEVKASAEYKLEPKEDNSFLGGIPQWVFFTAGSLVLLILFIIGCILFCTSTNNRRQR
RERRISMMRHVLDPQRTCQCHKMQYELYRP

LAG-3

Source: Genbank prediction XP_043935555

Sequence:

MLIILGFVLTSWLAGKVHSAPKLDVYAMVGSKAILPCFWLKVDYNQVTTYRKLSVLWRRKTLQSRGDFSTVLSVE
NSGLVKKGLVVLDRTFILESHFHTGNYSLRIEPVKMEDAGTYICRVDYGSESIQQEVQLHLMRVLPSLQRSPREGDS
LMLTCEITGELPQGIMLQWYHEKMQIVDNDQYWSTGTNKEKLSIRDLRSSYSGQWSCQLKQENRVTEAVYMLD
VIGFLNPVSQEVTICAGVGSVVELPCHVNDREPSIISGWTRQGLQVEPLQSRMNWNTDSRITIKEEGRGDLTMTI
SQVLESDSGIYSCFLQLPEKRMQRNIVLVVAKVSASISGMVKEGSSLKLVCDPDEVTGGDGFEWTHTEVISAADVL
PTWQSANLQSKTSYWGKTLTLKPVSYTDAGIWMCSVSKGGKRIGHIDYNLEISGSLLGDKTSEVTGKVLTAVIFSLII
VLLVVLLLLIAQRRRQRPREFPALENAIKLPPVTKEVFRKGQSSKMNPEKPCEMC

Tetrapods (Class: Sarcopterygii [lobe-finned fishes plus tetrapods]; Infraclass: Tetrapoda)

Xenopus tropicalis **(tropical clawed frog)**

CD4

Source: GenBank prediction XP_031762584

Sequence:

MERKLVTFISWLLFLQMDPSLTAPQTLQMWRKVEEKVLMPCNKNGAFRWRRNGLDYARMLGSQIVYGIGVESS
RISFPHGPNNSMQLTNLNMGDAGTYFCDAEEKLTINLVIFQISALPSANLIVSENLFLSIESSPKSIPGLRVSWETPN
RGKSEEKSELTVANAQIRDSGTYSCHVWIDGGNKATFTQYISVSGFYPTSEKIYVSKDSPALIPWLFNFNVRETALIN
TVSAVNGSISYSAGKGTSPSLISSLTVTSGACWPQRCEKSQGKEQLGNLSFVLPKPKAGRYHLEIQLKLGDRKKML
AMDVCLVMLTVSVLPRLQLPLEAKATLSCHASCSDANSTLYWHHENSNSVMHGQRGEPTFSQDLTAVPESMGV
WSCSVRVGGKTMMSTNLTLELEATFMSSPGFVWVLIGGGAVLFLVGIVTIVIVTARCRQKRRARRGAWILQNLH
QQRTCQCKGFAPTRLREKD

LAG-3

Source: Predicted from GenBank GL173463 (a genomic sequence)

Sequence:

MCLALFCLFLLAAGSAASPVRVYGAPGGRVTLPCNVSMGQQDRAHQSKYPAGSVSWRSNHKPVRMNSNGML
FRSRAVSSRVSIHPSPFELGDFSLHLDNVGEGDAGRYQGLAQYGGTKHECTVTLHIIGVTQSPLGDLPESSSVTLTC
SGASSLYNPHPRLRWLHGGNPVLPSNRFLQSENRLIIRALTQADRGEWSCELGGARASLQLTVLGISGPAFLSLYTS
VGVQAALPCTLNETPQGLLTVQWHHNAGSLDQNSQVLTISPVSSEDAGMYRCDITYKGHVMTRRIQLKVIQVYS
SGSAFMREGSALQLLCNVSGSDGGEKYVWTGPNPASGRRNQQDGAVLDLPAVQTGDTGVWNCSVYGKQGLV
GQLQYMLYVHAAQVSAFAAFSSWPTYVTFLLIILLVLGLISAISWHNRRRRLLHLLARTTIDVSSLKKVEV

***Chelonia mydas* (green sea turtle)**

CD4

Source: GenBank prediction XP_007069709

Sequence:

MNPFRVMAYSILAVFSVMQLGLIPTMAEGETTVFGAVGEQVILPCIGKSPNEGVTWKYNDLVVIQYQKQLLRGRT
PFFNRSELNKQEMSKGNFSLILSQLRHSDAGKYVCGVGSRTFMVQLQVFEVTGSPSGYLLQRKKLMLTIQGPSSA
NVTWYDNRKDKVTATQSRELKNGGHSLQIHNLRAEDSGTWTCHIASLSAKLDIPYKVLVIGFHHLNQETLYKAVN
STVSFSYSLSTDLQKIRKLEYITGGLEWKSMANNKYQEKFNFSATSKEPPLFKQIANMQFTWKLINHLEVKLPKVQF
KDAGWYQCQLTVSRGRVEKAIHLVVMTVSADPVEPLSKWANVTLLCKLSVPVPHNAQLFWEHVNGTEKELNM
LGYNEVMVKTKTVGLWRCSLNVENNVMTSIDYTVVKAAERNSCVWSWAGVGAGIVLLLLASLCTYIYIAQQQRR
QRAEKMARARRDLFERRTCQCQRQLKNDYSDA

LAG-3

Source: GenBank prediction XP_037745659

Sequence:

MTLAFMPLSLALMLLVNNASSTSPGAAKEQRGEQRVWAKVGDLVVLPCHLSPQELQSSWKQLYEKTAVRWERH
GESSHKEPHMVLEVEYSGLLKTARSMMPRASVRETSFRQGDFSLWIEPLRNDDAGHYEALVRYGKETRRCQLEL
GMVTVTVNPPGLLVETEPLLLLCNSSHPAKLVGMRWFHNGSLVPVSGRFRSRDGALSISRPTVSDSGPWSCELTY
SNDEKVSATFNLQILGFAGPASPVVYAAVGSATNLPCLLNRDPSASGILAVSAHWSRLAGGDLEIRGISRNGSNGSF
TLHLPEVGPGDAGQYCCAVSIHGTTITRDVTLAVMTVTPSIKGPVAEGSRLLLICSLTHPRGHEHFQWRQLSSGLS
NGSSFEATPRSPGVQRYYLGSTLELTRVSQEDIGTWECSVHSSEGRLGAVEYELYITGAQLSGPHTILDGQVSFGLIL
VLFLLLVASILALVLRNQRIFSPAFPALERVVTAPTPGNAVKDEGQEGKSLQTEC

***<u>Gallus gallus</u>* <u>(chicken)</u>**

CD4

Source: GenBank prediction XP_046762509

Sequence:

MERCGAVVSCVFAVILVLQLGLTPIMAQQEQQIGIAGKEVILSCKAINNQKDGTCTWKYKYKEVSSTIISFSKAQVF
KGKAPMTHRSELNSNSKKLKVSDLSLDDAGIYTCACYSPVVSISLHVFKLTISSNGHFLTNEDLELTLMQNSSHSQP
HLSIKLFNINNDIVTTEILQEEAPQKYILKLKQLKAIDSGTWMCHVYSNSPSINQNISFDVKVLGFEKERLEIIYTTVG
NTAILSWRLNFRKIKWKEGFTGKLNWEPQGNTAIHELLNFSVTTHQELHKTKKSNHIWFEISEGKTDGTMDVKIP
KVQLNHSGQYKCQLEINGRRTESVRALVVMQVTAIPAGPLSRGGKMTLLCQVSGPLPSNAHLLWERVNGTQME
MKKSKQHEAKVEVNVSAPGLWNCHLVEDNNKKISLNYTVEEAHVWNSYAVIGIIIGASVLVIGLACMCIITGMRW
QRRRKRARRMAQAKQYLLEKKTCQCQRRMYK

LAG-3

Source: GenBank prediction XP_416510

Sequence:

MRPVSPVLFLTLLAVNAGNILPGAAEVENRGQKVWAREGSSALLPCYLSPRKHGKSRKHLSGRTSVLWKRHGAS
APQELHVVLEVEYTGLRKTALPMRPRVSVLDSALRNGNFSLCINPVRSDDVGLYEAQVAYNTEILSCQVELGVVTV
TLSPPLPVVENEPLLLSCNSSHQASLVETCWFHNGRPVLISGAFCSSHGALYILRPTVSDSGSWHCQLRYADNEIIS
ATYNLQILGFDGPTNPVVYAAAGSAADLPCTLNYLPSASGIRVVKAHWSHFAGGHLQNWSILQNQSSRNFPLHL
PVVGPGDAGRYCCAVTVGNKMLSRDMTLAVITVTPRIQGPVSEGSHLLLTCSLTNPQGHEYFQWKHLSSASAEK
KLTVATSHNLKDSRVRTGPTLEIPQVSQKDVGIWECSVYGPEGRLGAVEYGLQITGAQVSGPPTIFSGQVTFGLML
TLFFLLMVCILALGLQKRARLPASFPALERIVAVTVPKEMEESQKETIQQTEC

***<u>Mus musculus</u>* <u>(mouse)</u>**

CD4

Source: GenBank NP_038516

Sequence:

MCRAISLRRLLLLLLQLSQLLAVTQGKTLVLGKEGESAELPCESSQKKITVFTWKFSDQRKILGQHGKGVLIRGGSPS
QFDRFDSKKGAWEKGSFPLIINKLKMEDSQTYICELENRKEEVELWVFKVTFSPGTSLLQGQSLTLTLDSNSKVSNP
LTECKHKKGKVVSGSKVLSMSNLRVQDSDFWNCTVTLDQKKNWFGMTLSVLGFQSTAITAYKSEGESAEFSFPLN
FAEENGWGELMWKAEKDSFFQPWISFSIKNKEVSVQKSTKDLKLQLKETLPLTLKIPQVSLQFAGSGNLTLTLDKG
TLHQEVNLVVMKVAQLNNTLTCEVMGPTSPKMRLTLKQENQEARVSEEQKVVQVVAPETGLWQCLLSEGDKVK
MDSRIQVLSRGVNQTVFLACVLGGSFGFLGFLGLCILCCVRCRHQQRQAARMSQIKRLLSEKKTCQCPHRMQKS
HNLI

LAG-3

Source: GenBank NP_032505

Sequence:

MREDLLLGFLLLGLLWEAPVVSSGPGKELPVVWAQEGAPVHLPCSLKSPNLDPNFLRRGGVIWQHQPDSGQPT
PIPALDLHQGMPSPRQPAPGRYTVLSVAPGGLRSGRQPLHPHVQLEERGLQRGDFSLWLRPALRTDAGEYHATV
RLPNRALSCSLRLRVGQASMIASPSGVLKLSDWVLLNCSFSRPDRPVSVHWFQGQNRVPVYNSPRHFLAETFLLL
PQVSPLDSGTWGCVLTYRDGFNVSITYNLKVLGLEPVAPLTVYAAEGSRVELPCHLPPGVGTPSLLIAKWTPPGGG
PELPVAGKSGNFTLHLEAVGLAQAGTYTCSIHLQGQQLNATVTLAVITVTPKSFGLPGSRGKLLCEVTPASGKERFV
WRPLNNLSRSCPGPVLEIQEARLLAERWQCQLYEGQRLLGATVYAAESSSGAHSARRISGDLKGGHLVLVLILGAL
SLFLLVAGAFGFHWWRKQLLLRRFSALEHGIQPFPAQRKIEELERELETEMGQEPEPEPEPQLEPEPRQL

***Homo sapiens* (human)**

CD4

Source: GenBank NP_000607

Sequence:

MNRGVPFRHLLLVLQLALLPAATQGKKVVLGKKGDTVELTCTASQKKSIQFHWKNSNQIKILGNQGSFLTKGPSKL
NDRADSRRSLWDQGNFPLIIKNLKIEDSDTYICEVEDQKEEVQLLVFGLTANSDTHLLQGQSLTLTLESPPGSSPSV
QCRSPRGKNIQGGKTLSVSQLELQDSGTWTCTVLQNQKKVEFKIDIVVLAFQKASSIVYKKEGEQVEFSFPLAFTV
EKLTGSGELWWQAERASSSKSWITFDLKNKEVSVKRVTQDPKLQMGKKLPLHLTLPQALPQYAGSGNLTLALEAK
TGKLHQEVNLVVMRATQLQKNLTCEVWGPTSPKLMLSLKLENKEAKVSKREKAVWVLNPEAGMWQCLLSDSG
QVLLESNIKVLPTWSTPVQPMALIVLGGVAGLLLFIGLGIFFCVRCRHRRRQAERMSQIKRLLSEKKTCQCPHRFQ
KTCSPI

LAG-3

Source: GenBank NP_002277

Sequence:

MWEAQFLGLLFLQPLWVAPVKPLQPGAEVPVVWAQEGAPAQLPCSPTIPLQDLSLLRRAGVTWQHQPDSGPP
AAAPGHPLAPGPHPAAPSSWGPRPRRYTVLSVGPGGLRSGRLPLQPRVQLDERGRQRGDFSLWLRPARRADAG
EYRAAVHLRDRALSCRLRLRLGQASMTASPPGSLRASDWVILNCSFSRPDRPASVHWFRNRGQGRVPVRESPHH
HLAESFLFLPQVSPMDSGPWGCILTYRDGFNVSIMYNLTVLGLEPPTPLTVYAGAGSRVGLPCRLPAGVGTRSFLT
AKWTPPGGGPDLLVTGDNGDFTLRLEDVSQAQAGTYTCHIHLQEQQLNATVTLAIITVTPKSFGSPGSLGKLLCEV
TPVSGQERFVWSSLDTPSQRSFSGPWLEAQEAQLLSQPWQCQLYQGERLLGAAVYFTELSSPGAQRSGRAPGAL
PAGHLLLFLILGVLSLLLLVTGAFGFHLWRRQWRPRRFSALEQGIHPPQAQSKIEELEQEPEPEPEPEPEPEPEPEPE
QL
