# Supplementary material for: CD4 and LAG-3 from sharks to humans: related molecules with motifs for opposing functions
Source: Front Immunol. 2023 Dec 21;14:1267743. doi: 10.3389/fimmu.2023.1267743 (PMC10768021; doi:10.3389/fimmu.2023.1267743)
Supplement: Supplementary file 4 [file DataSheet_4.pdf]

Supplementary file 4

Top-matches with shark CD4 and LAG-3 amino acid sequences among non-chondrichthyan sequences in GenBank

Deduced CD4 and LAG-3 amino acid sequences as listed in Supplementary file 2 for *Chiloscyllium griseum* (gray bambooshark), *Ginglymostoma cirratum* (nurse shark), *Scyliorhinus canicula* (smaller spotted catshark), *Scyliorhinus torazame* (cloudy catshark), and *Heterodontus zebra* (zebra bullhead shark) were compared by blastp analysis (https://blast.ncbi.nlm.nih.gov/Blast.cgi?PAGE=Proteins, accessed on 20 June 2023) with the non-redundant sequences at GenBank under the exclusion of sequences of Chondrichthyes. The searches were performed under default settings (Word size 5; Matrix BLOSUM62; Gap costs: Existence 11, Extension 1; Conditional compositional score matrix adjustment). For each of the searches, the top 10 matches are shown as depicted by the software. Most of these matches are LAG-3 sequences, probably because LAG-3 evolution is a bit more conservative (goes a bit slower), but there are also CD4 sequences among them, highlighting that after these hundreds of years of evolution the overall sequence similarity can still recognize a CD4/LAG-3 family signature but cannot distinguish well between CD4 and LAG-3.

The top-matches were:

Chiloscyllium griseum (gray bambooshark) CD4

	Description	Scientific Name	Max Score	Total Score	Query Cover	E value	Per. Ident	Acc. Len	Accession
☑	PREDICTED: lymphocyte activation gene 3 protein [Phaethon lepturus]	Phaethon lepturus	125	125	80%	2e-26	26.18%	507	XP_010288941.1
☑	lymphocyte activation gene 3 protein [Geotrypetes seraphini]	Geotrypetes seraphini	124	124	80%	3e-26	26.55%	511	XP_033781272.1
☑	PREDICTED: lymphocyte activation gene 3 protein [Colius striatus]	Colius striatus	123	123	82%	7e-26	27.20%	507	XP_010204371.1
☑	lymphocyte activation gene 3 protein [Oxyura jamaicensis]	Oxyura jamaicensis	123	123	80%	9e-26	25.11%	505	XP_035199058.1
☑	PREDICTED: lymphocyte activation gene 3 protein [Sturnus vulgaris]	Sturnus vulgaris	122	122	81%	1e-25	24.73%	506	XP_014739614.1
☑	LOW QUALITY PROTEIN: lymphocyte activation gene 3 protein [Anser cygnoides]	Anser cygnoides	122	122	80%	2e-25	25.27%	505	XP_013054937.2
☑	lymphocyte activation gene 3 protein [Anas platyrhynchos]	Anas platyrhynchos	121	121	81%	2e-25	25.32%	505	XP_027305580.1
☑	PREDICTED: lymphocyte activation gene 3 protein [Fulmarus glacialis]	Fulmarus glacialis	121	121	80%	3e-25	27.02%	507	XP_009572768.1
☑	PREDICTED: lymphocyte activation gene 3 protein [Balearica regulorum gibbericeps]	Balearica regulorum gibbericeps	121	121	80%	4e-25	26.34%	507	XP_010310182.1
☑	LOW QUALITY PROTEIN: lymphocyte activation gene 3 protein [Cygnus atratus]	Cygnus atratus	121	121	80%	4e-25	25.27%	505	XP_035401653.1

Chiloscyllium griseum (gray bambooshark) LAG-3

	Description	Scientific Name	Max Score	Total Score	Query Cover	E value	Per. Ident	Acc. Len	Accession
☑	T-cell surface glycoprotein CD4-like isoform X2 [Protopterus annectens]	Protopterus annectens	144	144	94%	1e-33	27.81%	509	XP_043935555.1
☑	T-cell surface glycoprotein CD4-like isoform X1 [Protopterus annectens]	Protopterus annectens	139	139	88%	1e-31	27.11%	510	XP_043935554.1
☑	PREDICTED: lymphocyte activation gene 3 protein isoform X1 [Latimeria chalumnae]	Latimeria chalumnae	137	137	98%	5e-31	26.17%	505	XP_014349213.1
☑	PREDICTED: lymphocyte activation gene 3 protein isoform X2 [Latimeria chalumnae]	Latimeria chalumnae	134	134	95%	5e-30	26.37%	484	XP_006004759.1
☑	PREDICTED: lymphocyte activation gene 3 protein [Sturnus vulgaris]	Sturnus vulgaris	134	134	91%	6e-30	28.97%	506	XP_014739614.1
☑	PREDICTED: lymphocyte activation gene 3 protein [Phaethon lepturus]	Phaethon lepturus	133	133	91%	1e-29	29.03%	507	XP_010288941.1
☑	hypothetical protein DV515_00010167 [Chloebia gouldiae]	Chloebia gouldiae	132	132	91%	2e-29	29.12%	505	RLV99057.1
☑	lymphocyte activation gene 3 protein [Athene cunicularia]	Athene cunicularia	130	130	91%	1e-28	29.42%	502	XP_026712062.1
☑	PREDICTED: lymphocyte activation gene 3 protein [Balearica regulorum gibbericeps]	Balearica regulorum gibbericeps	129	129	91%	3e-28	29.18%	507	XP_010310182.1
☑	PREDICTED: lymphocyte activation gene 3 protein [Pseudopodoces humilis]	Pseudopodoces humilis	129	129	91%	4e-28	28.91%	506	XP_005523892.1

Ginglymostoma cirratum (nurse shark) CD4

	Description	Scientific Name	Max Score	Total Score	Query Cover	E value	Per. Ident	Acc. Len	Accession
☑	lymphocyte activation gene 3 protein [Podarcis muralis]	Podarcis muralis	110	110	66%	9e-22	26.54%	490	XP_028568687.1
☑	lymphocyte activation gene 3 protein [Lacerta agilis]	Lacerta agilis	110	110	66%	1e-21	25.94%	498	XP_033029458.1
☑	PREDICTED: lymphocyte activation gene 3 protein [Anolis carolinensis]	Anolis carolinensis	102	102	58%	1e-19	27.44%	362	XP_008118461.2
☑	CD4-1 molecule isoform X1 [Polypterus senegalus]	Polypterus senegalus	103	103	67%	2e-19	23.08%	480	XP_039619589.1
☑	Lymphocyte activation 3-like protein [Ophiophagus hannah]	Ophiophagus hannah	103	103	47%	2e-19	28.62%	420	ETE72093.1
☑	lymphocyte activation gene 3 protein [Eublepharis macularius]	Eublepharis macularius	104	104	67%	2e-19	25.77%	526	XP_054858893.1
☑	CD4-1 molecule isoform X2 [Polypterus senegalus]	Polypterus senegalus	103	103	67%	2e-19	22.88%	479	XP_039619593.1
☑	CD4-1 molecule isoform X1 [Erpetoichthys calabaricus]	Erpetoichthys calabaricus	102	102	70%	5e-19	23.57%	477	XP_028665851.1
☑	lymphocyte activation gene 3 protein [Protobothrops mucrosquamatus]	Protobothrops mucrosquamatus	103	103	47%	5e-19	28.62%	530	XP_015669534.2
☑	lymphocyte activation gene 3 protein [Notechis scutatus]	Notechis scutatus	102	102	47%	5e-19	28.62%	488	XP_026542053.1

Ginglymostoma cirratum (nurse shark) LAG-3

	Description	Scientific Name	Max Score	Total Score	Query Cover	E value	Per. Ident	Acc. Len	Accession
☑	diverse immunoglobulin domain-containing protein 2.1 [Polyodon spathula]	Polyodon spathula	138	138	95%	2e-31	27.77%	495	XP_041123852.1
☑	lymphocyte activation gene 3 protein-like [Acipenser ruthenus]	Acipenser ruthenus	134	134	96%	8e-30	28.29%	495	XP_033897857.1
☑	lymphocyte activation gene 3 protein-like isoform X1 [Erpetoichthys calabaricus]	Erpetoichthys calabaricus	126	126	97%	3e-27	24.44%	498	XP_028665848.2
☑	lymphocyte activation gene 3 protein-like isoform X1 [Polypterus senegalus]	Polypterus senegalus	124	124	97%	2e-26	23.63%	498	XP_039619595.1
☑	lymphocyte activation gene 3 protein-like isoform X2 [Erpetoichthys calabaricus]	Erpetoichthys calabaricus	122	122	80%	4e-26	24.94%	449	XP_028665849.2
☑	lymphocyte activation 3 protein [Alligator mississippiensis]	Alligator mississippiensis	123	123	94%	4e-26	29.88%	505	KYO22308.1
☑	PREDICTED: lymphocyte activation gene 3 protein [Gavialis gangeticus]	Gavialis gangeticus	121	121	79%	1e-25	30.94%	445	XP_019382326.1
☑	PREDICTED: lymphocyte activation gene 3 protein [Alligator mississippiensis]	Alligator mississippiensis	121	121	88%	1e-25	29.78%	445	XP_019331667.1
☑	PREDICTED: lymphocyte activation gene 3 protein [Crocodylus porosus]	Crocodylus porosus	122	122	94%	1e-25	30.00%	506	XP_019404203.1
☑	lymphocyte activation gene 3 protein-like isoform X2 [Polypterus senegalus]	Polypterus senegalus	120	120	80%	2e-25	24.38%	449	XP_039619596.1

Scyliorhinus canicula (smaller spotted catshark) CD4

Description	Scientific Name	Max Score	Total Score	Query Cover	E value	Per. Ident	Acc. Len	Accession
lymphocyte activation gene 3 protein [Oenanthe melanoleuca]	Oenanthe melanoleuca	142	142	81%	1e-32	26.26%	498	XP_056337966.1
PREDICTED: lymphocyte activation gene 3 protein [Sturnus vulgaris]	Sturnus vulgaris	141	141	82%	3e-32	25.81%	506	XP_014739614.1
PREDICTED: lymphocyte activation gene 3 protein [Balearica regulorum gibbericeps]	Balearica regulorum gibbericeps	135	135	84%	5e-30	26.27%	507	XP_010310182.1
PREDICTED: lymphocyte activation gene 3 protein [Pseudopodoces humilis]	Pseudopodoces humilis	135	135	82%	6e-30	25.43%	506	XP_005523892.1
lymphocyte activation gene 3 protein [Cyanistes caeruleus]	Cyanistes caeruleus	134	134	82%	7e-30	25.21%	506	XP_023795721.1
lymphocyte activation gene 3 protein [Parus major]	Parus major	134	134	81%	7e-30	25.32%	506	XP_015473376.1
lymphocyte activation gene 3 protein [Dryobates pubescens]	Dryobates pubescens	134	134	85%	2e-29	26.32%	504	XP_009897909.2
lymphocyte activation gene 3 protein [Onychostruthus taczanowskii]	Onychostruthus taczanowskii	134	134	81%	2e-29	24.30%	506	XP_041266992.1
LOW QUALITY PROTEIN: lymphocyte activation gene 3 protein [Anser cygnoides]	Anser cygnoides	133	133	85%	2e-29	26.20%	505	XP_013054937.2
LOW QUALITY PROTEIN: lymphocyte activation gene 3 protein [Cygnus atratus]	Cygnus atratus	133	133	81%	2e-29	25.97%	505	XP_035401653.1

Scyliorhinus canicula (smaller spotted catshark) LAG-3

Description	Scientific Name	Max Score	Total Score	Query Cover	E value	Per. Ident	Acc. Len	Accession
diverse immunoglobulin domain-containing protein 2.1 [Polyodon spathula]	Polyodon spathula	141	141	93%	1e-32	30.48%	495	XP_041123852.1
PREDICTED: lymphocyte activation gene 3 protein isoform X1 [Latimeria chalumnae]	Latimeria chalumnae	139	139	99%	1e-31	25.25%	505	XP_014349213.1
PREDICTED: lymphocyte activation gene 3 protein isoform X2 [Latimeria chalumnae]	Latimeria chalumnae	137	137	95%	4e-31	25.42%	484	XP_006004759.1
T-cell surface glycoprotein CD4-like isoform X2 [Protopterus annectens]	Protopterus annectens	127	127	95%	2e-27	28.13%	509	XP_043935555.1
hypothetical protein NDU88_000921 [Pleurodeles waltl]	Pleurodeles waltl	126	126	95%	2e-27	26.90%	493	KAJ1122434.1
lymphocyte activation gene 3 protein [Podarcis muralis]	Podarcis muralis	125	125	92%	8e-27	27.25%	490	XP_028568687.1
lymphocyte activation gene 3 protein-like [Acipenser ruthenus]	Acipenser ruthenus	124	124	93%	1e-26	27.98%	495	XP_033897857.1
T-cell surface glycoprotein CD4-like isoform X1 [Protopterus annectens]	Protopterus annectens	123	123	84%	4e-26	28.38%	510	XP_043935554.1
lymphocyte activation gene 3 protein [Lacerta agilis]	Lacerta agilis	117	117	92%	4e-24	26.46%	498	XP_033029458.1
lymphocyte activation gene 3 protein [Sphaerodactylus townsendi]	Sphaerodactylus townsendi	115	115	95%	2e-23	26.99%	500	XP_048360351.1

Scyliorhinus torazame (cloudy catshark) CD4

Description	Scientific Name	Max Score	Total Score	Query Cover	E value	Per. Ident	Acc. Len	Accession
LOW QUALITY PROTEIN: lymphocyte activation gene 3 protein [Cygnus atratus]	Cygnus atratus	145	145	81%	2e-33	26.72%	505	XP_035401653.1
LOW QUALITY PROTEIN: lymphocyte activation gene 3 protein [Anser cygnoides]	Anser cygnoides	144	144	85%	4e-33	26.51%	505	XP_013054937.2
lymphocyte activation gene 3 protein [Oenanthe melanoleuca]	Oenanthe melanoleuca	143	143	81%	5e-33	25.77%	498	XP_056337966.1
PREDICTED: lymphocyte activation gene 3 protein [Chaetura pelagica]	Chaetura pelagica	141	141	73%	3e-32	28.12%	501	XP_009996861.1
PREDICTED: lymphocyte activation gene 3 protein [Charadrius vociferus]	Charadrius vociferus	140	140	74%	6e-32	27.19%	507	XP_009890770.1
lymphocyte activation gene 3 protein isoform X3 [Grus americana]	Grus americana	139	139	78%	9e-32	27.17%	451	XP_054690091.1
lymphocyte activation gene 3 protein isoform X1 [Grus americana]	Grus americana	140	140	78%	1e-31	27.06%	507	XP_054690064.1
PREDICTED: lymphocyte activation gene 3 protein [Phaethon lepturus]	Phaethon lepturus	138	138	81%	5e-31	26.45%	507	XP_010288941.1
PREDICTED: lymphocyte activation gene 3 protein [Balearica regulorum gibbericeps]	Balearica regulorum gibbericeps	137	137	86%	9e-31	26.29%	507	XP_010310182.1
PREDICTED: lymphocyte activation gene 3 protein [Pelecanus crispus]	Pelecanus crispus	137	137	74%	1e-30	26.87%	507	XP_009482985.1

Scyliorhinus torazame (cloudy catshark) LAG-3

	Description	Scientific Name	Max Score	Total Score	Query Cover	E value	Per. Ident	Acc. Len	Accession
☑	PREDICTED: lymphocyte activation gene 3 protein isoform X2 [Latimeria chalumnae]	Latimeria chalumnae	147	147	94%	7e-35	25.85%	484	XP_006004759.1
☑	PREDICTED: lymphocyte activation gene 3 protein isoform X1 [Latimeria chalumnae]	Latimeria chalumnae	147	147	99%	1e-34	25.90%	505	XP_014349213.1
☑	diverse immunoglobulin domain-containing protein 2.1 [Polyodon spathula]	Polyodon spathula	146	146	96%	2e-34	27.58%	495	XP_041123852.1
☑	T-cell surface glycoprotein CD4-like isoform X2 [Protopterus annectens]	Protopterus annectens	133	133	95%	2e-29	27.87%	509	XP_043935555.1
☑	hypothetical protein NDU88_000921 [Pleurodeles waltl]	Pleurodeles waltl	132	132	95%	3e-29	27.14%	493	KAJ1122434.1
☑	T-cell surface glycoprotein CD4-like isoform X1 [Protopterus annectens]	Protopterus annectens	131	131	84%	5e-29	28.38%	510	XP_043935554.1
☑	lymphocyte activation gene 3 protein-like [Acipenser ruthenus]	Acipenser ruthenus	127	127	96%	1e-27	26.48%	495	XP_033897857.1
☑	lymphocyte activation gene 3 protein [Eublepharis macularius]	Eublepharis macularius	119	119	95%	1e-24	27.00%	526	XP_054858893.1
☑	lymphocyte activation gene 3 protein [Podarcis muralis]	Podarcis muralis	117	117	92%	6e-24	26.74%	490	XP_028568687.1
☑	lymphocyte activation gene 3 protein-like isoform X1 [Polypterus senegalus]	Polypterus senegalus	112	112	95%	2e-22	23.67%	498	XP_039619595.1

Heterodontus zebra (zebra bullhead shark) CD4

	Description	Scientific Name	Max Score	Total Score	Query Cover	E value	Per. Ident	Acc. Len	Accession
☑	lymphocyte activation gene 3 protein [Oenanthe melanoleuca]	Oenanthe melanoleuca	130	130	79%	3e-28	26.68%	498	XP_056337966.1
☑	diverse immunoglobulin domain-containing protein 2.1 [Polyodon spathula]	Polyodon spathula	127	127	66%	4e-27	27.74%	495	XP_041123852.1
☑	PREDICTED: lymphocyte activation gene 3 protein [Ficedula albicollis]	Ficedula albicollis	124	124	84%	5e-26	25.51%	506	XP_016153056.1
☑	PREDICTED: lymphocyte activation gene 3 protein isoform X2 [Latimeria chalumnae]	Latimeria chalumnae	120	230	82%	6e-25	26.63%	484	XP_006004759.1
☑	lymphocyte activation gene 3 protein [Phasianus colchicus]	Phasianus colchicus	120	120	84%	8e-25	26.18%	504	XP_031466144.1
☑	PREDICTED: lymphocyte activation gene 3 protein isoform X1 [Latimeria chalumnae]	Latimeria chalumnae	119	233	82%	1e-24	26.63%	505	XP_014349213.1
☑	PREDICTED: lymphocyte activation gene 3 protein [Calidris pugnax]	Calidris pugnax	119	119	84%	1e-24	24.80%	508	XP_014812618.1
☑	lymphocyte activation gene 3 protein [Apus apus]	Apus apus	119	119	77%	1e-24	24.17%	506	XP_051491895.1
☑	lymphocyte activation gene 3 protein [Melopsittacus undulatus]	Melopsittacus undulatus	119	119	80%	2e-24	24.95%	507	XP_005146701.2
☑	hypothetical protein NDU88_000921 [Pleurodeles waltl]	Pleurodeles waltl	119	119	81%	2e-24	26.28%	493	KAJ1122434.1

Heterodontus zebra (zebra bullhead shark) LAG-3

	Description	Scientific Name	Max Score	Total Score	Query Cover	E value	Per. Ident	Acc. Len	Accession
☑	PREDICTED: lymphocyte activation gene 3 protein isoform X2 [Latimeria chalumnae]	Latimeria chalumnae	180	180	93%	1e-46	27.80%	484	XP_006004759.1
☑	PREDICTED: lymphocyte activation gene 3 protein isoform X1 [Latimeria chalumnae]	Latimeria chalumnae	180	180	93%	1e-46	27.80%	505	XP_014349213.1
☑	lymphocyte activation gene 3 protein [Podarcis muralis]	Podarcis muralis	154	154	93%	3e-37	28.90%	490	XP_028568687.1
☑	lymphocyte activation gene 3 protein [Lacerta agilis]	Lacerta agilis	152	152	88%	3e-36	29.75%	498	XP_033029458.1
☑	PREDICTED: lymphocyte activation gene 3 protein [Balearica regulorum gibbericeps]	Balearica regulorum gibbericeps	150	150	94%	1e-35	28.69%	507	XP_010310182.1
☑	PREDICTED: lymphocyte activation gene 3 protein [Phaethon lepturus]	Phaethon lepturus	144	144	95%	2e-33	29.28%	507	XP_010288941.1
☑	PREDICTED: lymphocyte activation gene 3 protein [Gavialis gangeticus]	Gavialis gangeticus	142	142	86%	3e-33	32.04%	445	XP_019382326.1
☑	hypothetical protein NDU88_000921 [Pleurodeles waltl]	Pleurodeles waltl	143	143	95%	3e-33	28.54%	493	KAJ1122434.1
☑	diverse immunoglobulin domain-containing protein 2.1 [Polyodon spathula]	Polyodon spathula	143	143	98%	4e-33	25.59%	495	XP_041123852.1
☑	PREDICTED: lymphocyte activation gene 3 protein [Colius striatus]	Colius striatus	143	143	97%	5e-33	29.20%	507	XP_010204371.1
