# Supplementary material for: CD4 and LAG-3 from sharks to humans: related molecules with motifs for opposing functions
Source: Front Immunol. 2023 Dec 21;14:1267743. doi: 10.3389/fimmu.2023.1267743 (PMC10768021; doi:10.3389/fimmu.2023.1267743)

Supplementary file 5

Phylogenetic tree analysis of deduced C*D4* and *LAG-3* amino acid sequences by Maximum Likelihood method

The figures show Maximum Likelihood (ML) trees of alignments made by the "MUSCLE" program in the MEGA7 software for all CD4 and LAG-3 sequences listed in Supplementary file 2 (A) or only those in that list in the more primitive species while excluding those in gar, teleosts, and tetrapods above the level of amphibians (B). The latter was done to exclude some of the CD4 and LAG-3 sequences that showed a more divergent evolution.

The evolutionary histories were inferred by using the Maximum Likelihood method based on the JTT matrix-based model [1]. The trees with the highest log likelihood are shown. The percentage of trees in which the associated taxa clustered together is shown next to the branches. Initial tree(s) for the heuristic search were obtained automatically by applying Neighbor-Join and BioNJ algorithms to a matrix of pairwise distances estimated using a JTT model, and then selecting the topology with superior log likelihood value. The tree is drawn to scale, with branch lengths measured in the number of substitutions per site. All positions with less than 95% site coverage were eliminated. That is, fewer than 5% alignment gaps, missing data, and ambiguous bases were allowed at any position. There were a total of 255 positions in the final dataset. Evolutionary analyses were conducted in MEGA7 [2].

1. Jones D.T., Taylor W.R., and Thornton J.M. (1992). The rapid generation of mutation data matrices from protein sequences. Computer Applications in the Biosciences 8: 275-282.

2. Kumar S., Stecher G., and Tamura K. (2016). MEGA7: Molecular Evolutionary Genetics Analysis version 7.0 for bigger datasets.Molecular Biology and Evolution 33:1870-1874.

(A) MJ phylogenetic tree of all the CD4 and LAG-3 sequences in Supplementary file 2

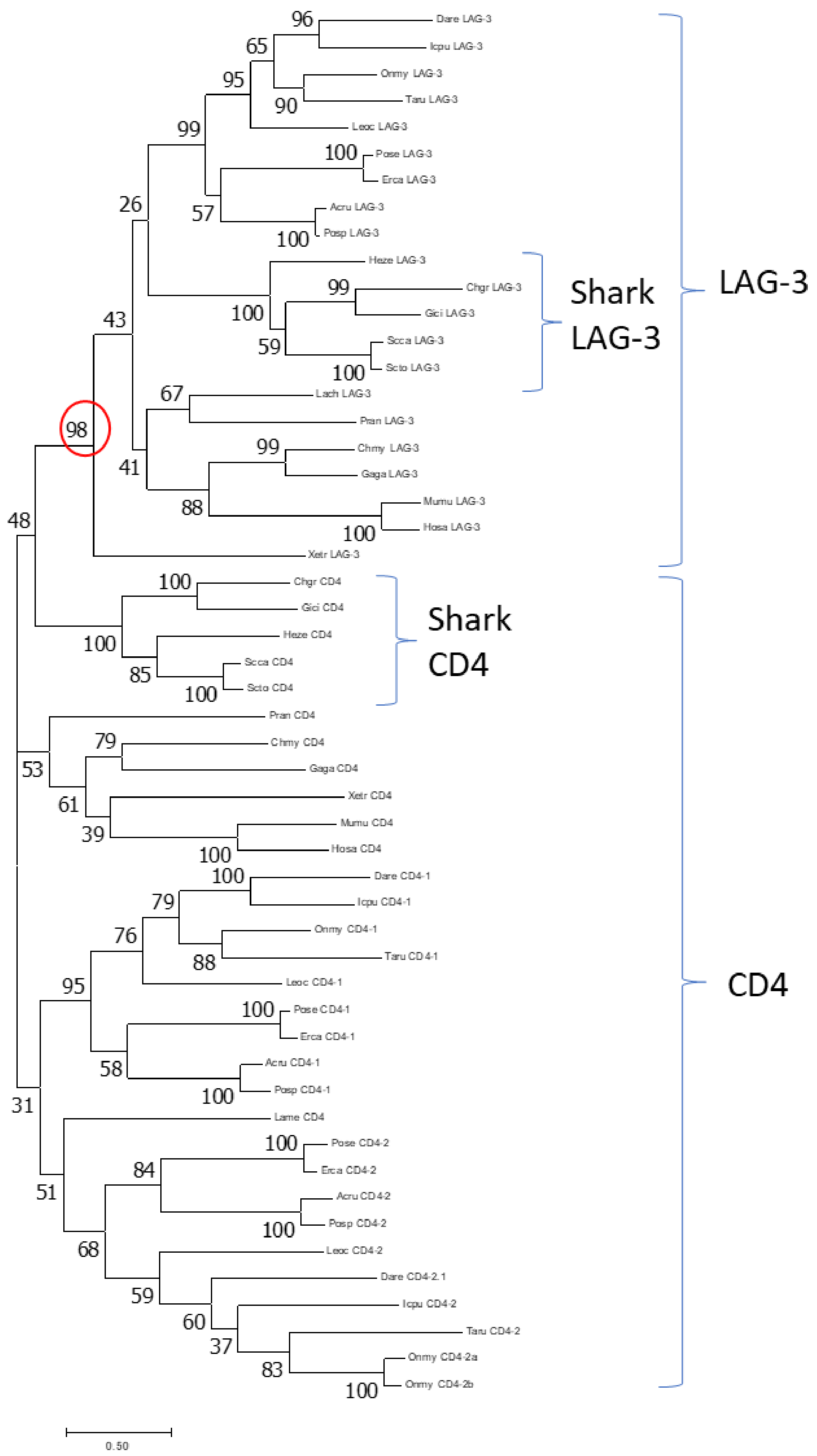

(B) MJ phylogenetic tree of the CD4 and LAG-3 sequences in Supplementary file 2 with the exclusion of those in gar, ray-finned fish, and tetrapod species more evolved than amphibians

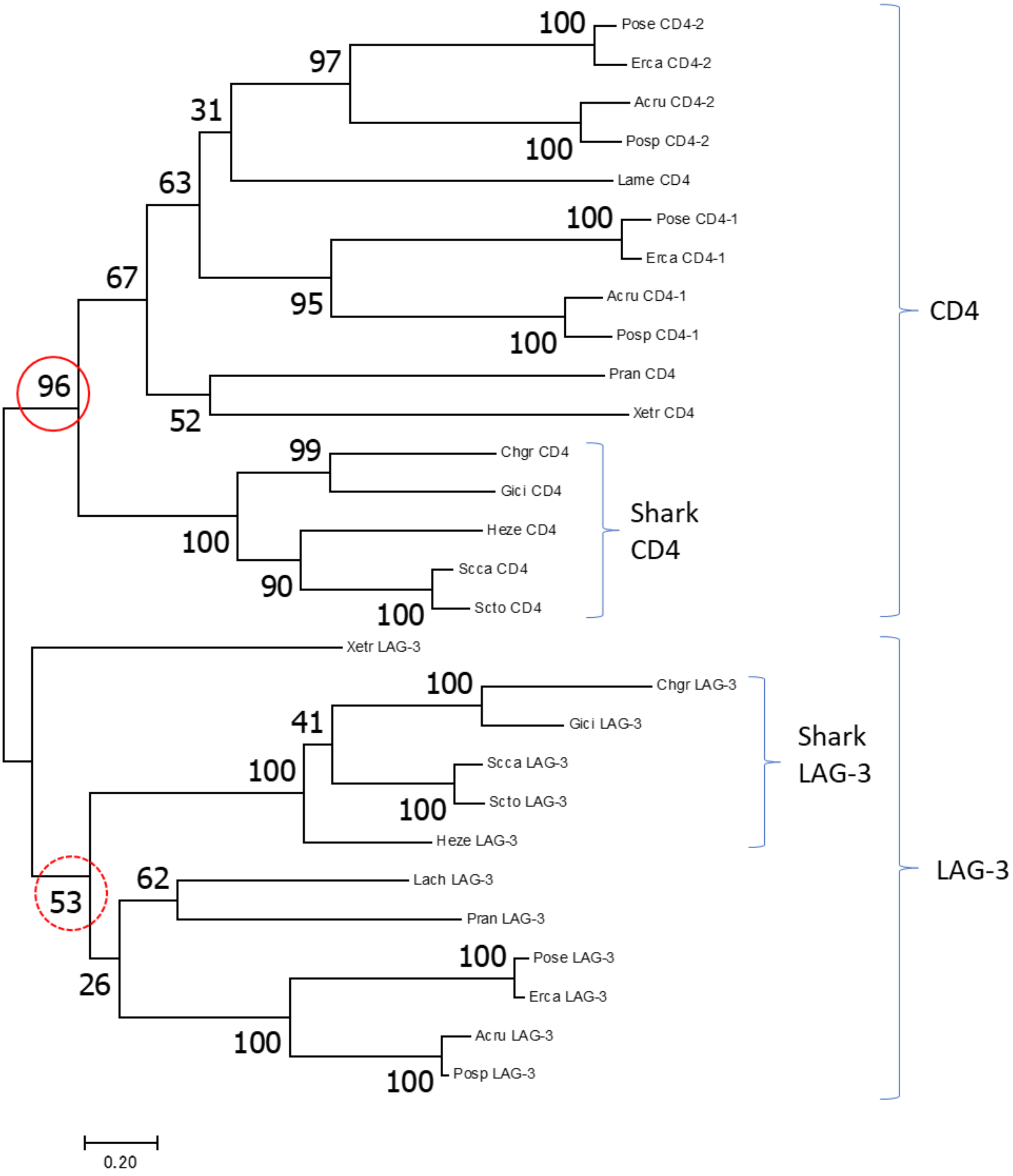

Supplement: Supplementary file 5 [file DataSheet_5.pdf]
