# Supplementary material for: CD4 and LAG-3 from sharks to humans: related molecules with motifs for opposing functions
Source: Front Immunol. 2023 Dec 21;14:1267743. doi: 10.3389/fimmu.2023.1267743 (PMC10768021; doi:10.3389/fimmu.2023.1267743)
Supplement: Supplementary file 6 [file DataSheet_6.pdf]

Supplementary file 6

Top-matches between CD4 and LAG-3 of non-chondrichthyan species and shark CD4 and LAG-3

In Supplementary file 4, we compared the shark CD4 and LAG-3 sequences listed in Supplementary file 3 (and shown in Fig. 3) with all non-chondrichthyan protein sequences in the NCBI non-redundant protein database. Although this clearly confirmed a CD4/LAG-3 family signature, the large variation in available CD4 and LAG-3 sequences seems to have prohibited a consistent finding of orthologues (CD4 for CD4, LAG-3 for LAG-3) as top-matches.

Therefore, shown in the present file, to reduce the possibility that "coincidental" matches in varying sequences affect the CD4 versus LAG-3 identity of the top-matches found, we also compared all non-chondrichthyan CD4 and LAG-3 sequences listed in Supplementary file 2 with the combined ten CD4 and LAG-3 sequences of sharks listed in that file. Compared to CD4 and LAG-3 sequences in some other species, these shark sequences are relatively conservative. For comparison we used the "BLASTP / Align 2 or more sequences" function of NCBI (https://blast.ncbi.nlm.nih.gov) with default settings (Matrix, BLOSUM62; Existence 11; Extension 1; Conditional compositional score matrix adjustment).

The detailed results are included at the end of this file, showing that generally the orthologous sequences do give the best matches but that these overall full-length similarity distinctions between CD4 and LAG-3 are only very subtle. The short summary of these results is that from the 42 analyzed CD4 and LAG-3 sequences only three did not retrieve an orthologous sequence as top-match.

The longer summary of the results is shown here, listing the shark top-match for the different CD4 and LAG-3 molecules and with the three top-matches that were not orthologous underlined and in Italic font:

Gray-bichir CD4-1	:	Nurse shark CD4
Gray-bichir CD4-2	:	Cloudy catshark CD4
Gray-bichir LAG-3	:	Nurse shark LAG-3
Reedfish CD4-1	:	Nurse shark CD4
Reedfish CD4-2	:	Cloudy catshark CD4
Reedfish LAG-3	:	Nurse shark LAG-3
Sterlet sturgeon CD4-1	:	Cloudy catshark CD4
Sterlet sturgeon CD4-2	:	Cloudy catshark CD4
Sterlet sturgeon LAG-3	:	Nurse shark LAG-3
M. paddlefish CD4-1	:	Small-spotted catshark CD4
M. paddlefish CD4-2	:	Cloudy catshark CD4

M. paddlefish LAG-3	:	Cloudy catshark LAG-3
Spotted gar CD4-1	:	Zebra bullhead shark CD4
Spotted gar CD4-2	:	Small-spotted catshark CD4
Spotted gar LAG-3	:	Small-spotted catshark LAG-3
Zebrafish CD4-1	:	Cloudy catshark CD4
Zebrafish CD4-2.1	:	Gray bambooshark CD4
Zebrafish LAG-3	:	Nurse shark LAG-3
Channel catfish CD4-1	:	Cloudy catshark CD4
Channel catfish CD4-2	:	Small-spotted catshark CD4
Channel catfish LAG-3	:	Nurse shark LAG-3
Rainbow trout CD4-1	:	Small-spotted catshark CD4
Rainbow trout CD4-2a	:	Cloudy catshark CD4
Rainbow trout CD4-2b	:	Cloudy catshark CD4
Rainbow trout LAG-3	*:*	*Zebra bullhead shark CD4*
Fugu CD4-1	:	Small-spotted catshark CD4
Fugu CD4-2	:	Small-spotted catshark CD4
Fugu LAG-3	:	Gray bambooshark LAG-3
Coelacanth CD4	:	Cloudy catshark CD4
Coelacanth LAG-3	:	Zebra bullhead shark LAG-3
W.A. lungfish CD4	:	Zebra bullhead shark CD4
W.A. lungfish LAG-3	:	Zebra bullhead shark LAG-3
Tr. clawed frog CD4	:	Small-spotted catshark CD4
Tr. clawed frog LAG-3	:	Small-spotted catshark LAG-3
Green sea turtle CD4	:	Cloudy catshark CD4
Green sea turtle LAG-3	:	Zebra bullhead shark LAG-3
Chicken CD4	:	Gray bambooshark CD4
Chicken LAG-3	*:*	*Cloudy catshark CD4*
Mouse CD4	*:*	*Nurse shark LAG-3*
Mouse LAG-3	:	Zebra bullhead shark LAG-3
Human CD4	:	Zebra bullhead shark CD4
Human LAG-3	:	Zebra bullhead shark LAG-3

The detailed results, as provided by the NCBI BLASTP software, were (abbreviations are Chgr: Chiloscyllium griseum [gray bambooshark]; Gici: Ginglymostoma cirratum [nurse shark]; Scca: Scyliorhinus canicula [small-spotted catshark]; Scto: Scyliorhinus torazame [cloudy catshark]; Heze: Heterodontus zebra [zebra bullhead shark]):

***Polypterus senegalus* (gray bichir)**

CD4-1

Descriptions | Graphic Summary | Alignments

Sequences producing significant alignments — Download — Select columns — Show 100

select all *10 sequences selected* — Graphics — Distance tree of results — Multiple alignment — MSA Viewer

Description	Scientific Name	Max Score	Total Score	Query Cover	E value	Per. Ident	Acc. Len	Accession
Gici_CD4		96.3	122	74%	1e-24	22.70%	532	Query_56406
Scto_CD4		87.8	87.8	91%	8e-22	20.86%	525	Query_56410
Heze_CD4		85.9	85.9	82%	4e-21	20.05%	533	Query_56412
Chgr_CD4		84.3	115	74%	1e-20	21.93%	535	Query_56404
Scca_CD4		82.4	82.4	87%	4e-20	21.00%	525	Query_56408
Scto_LAG-3		61.2	108	86%	2e-13	22.86%	480	Query_56411
Scca_LAG-3		57.0	100	85%	4e-12	22.25%	474	Query_56409
Gici_LAG-3		50.8	119	71%	4e-10	21.97%	488	Query_56407
Heze_LAG-3		42.4	94.7	66%	2e-07	21.16%	489	Query_56413
Chgr_LAG-3		39.3	102	58%	2e-06	18.60%	486	Query_56405

CD4-2

Descriptions | Graphic Summary | Alignments

Sequences producing significant alignments — Download — Select columns — Show 100

select all *10 sequences selected* — Graphics — Distance tree of results — Multiple alignment — MSA Viewer

Description	Scientific Name	Max Score	Total Score	Query Cover	E value	Per. Ident	Acc. Len	Accession
Scto_CD4		77.4	159	72%	1e-18	22.12%	525	Query_76372
Scca_CD4		75.5	173	75%	6e-18	23.05%	525	Query_76370
Heze_CD4		71.6	204	57%	9e-17	22.67%	533	Query_76374
Chgr_CD4		67.8	150	77%	1e-15	24.71%	535	Query_76366
Gici_LAG-3		50.1	159	56%	6e-10	25.25%	488	Query_76369
Scca_LAG-3		49.7	75.5	71%	7e-10	20.12%	474	Query_76371
Gici_CD4		48.1	131	81%	3e-09	20.06%	532	Query_76368
Heze_LAG-3		45.8	122	63%	1e-08	23.84%	489	Query_76375
Scto_LAG-3		44.3	113	68%	4e-08	18.94%	480	Query_76373
Chgr_LAG-3		30.4	141	67%	9e-04	22.29%	486	Query_76367

LAG-3

Descriptions | Graphic Summary | Alignments

Sequences producing significant alignments Download Select columns Show 100

select all *10 sequences selected* Graphics Distance tree of results Multiple alignment MSA Viewer

Description	Scientific Name	Max Score	Total Score	Query Cover	E value	Per. Ident	Acc. Len	Accession
Gici_LAG-3		120	120	83%	1e-32	24.53%	488	Query_17017
Scto_LAG-3		107	129	94%	2e-28	23.72%	480	Query_17021
Scca_LAG-3		100	129	90%	4e-26	23.24%	474	Query_17019
Heze_LAG-3		99.4	142	95%	1e-25	22.15%	489	Query_17023
Scto_CD4		95.5	140	84%	3e-24	22.52%	525	Query_17020
Chgr_LAG-3		90.5	115	80%	1e-22	22.84%	486	Query_17015
Heze_CD4		85.1	175	80%	7e-21	22.97%	533	Query_17022
Scca_CD4		84.7	123	80%	1e-20	21.33%	525	Query_17018
Gici_CD4		77.4	115	76%	2e-18	21.41%	532	Query_17016
Chgr_CD4		65.5	115	86%	1e-14	21.51%	535	Query_17014

Erpetoichthys calabaricus **(Reedfish)**

CD4-1

Descriptions | Graphic Summary | Alignments

Sequences producing significant alignments Download Select columns Show 100

select all *10 sequences selected* Graphics Distance tree of results Multiple alignment MSA Viewer

Description	Scientific Name	Max Score	Total Score	Query Cover	E value	Per. Ident	Acc. Len	Accession
Gici_CD4		95.5	125	89%	2e-24	23.25%	532	Query_27910
Scto_CD4		92.4	92.4	90%	2e-23	20.69%	525	Query_27914
Chgr_CD4		91.7	154	78%	4e-23	23.14%	535	Query_27908
Heze_CD4		85.9	117	82%	3e-21	21.63%	533	Query_27916
Scca_CD4		84.0	84.0	87%	1e-20	20.90%	525	Query_27912
Scca_LAG-3		77.8	127	85%	1e-18	23.06%	474	Query_27913
Scto_LAG-3		75.1	132	88%	9e-18	23.90%	480	Query_27915
Heze_LAG-3		71.2	108	91%	2e-16	21.74%	489	Query_27917
Gici_LAG-3		48.9	95.9	84%	2e-09	23.61%	488	Query_27911
Chgr_LAG-3		38.9	124	68%	3e-06	20.42%	486	Query_27909

CD4-2

Descriptions | Graphic Summary | Alignments

Sequences producing significant alignments Download Select columns Show 100

select all *10 sequences selected* Graphics Distance tree of results Multiple alignment MSA Viewer

Description	Scientific Name	Max Score	Total Score	Query Cover	E value	Per. Ident	Acc. Len	Accession
Scto_CD4		90.9	165	74%	4e-23	22.50%	525	Query_97054
Heze_CD4		85.1	157	74%	4e-21	21.79%	533	Query_97056
Scca_CD4		80.5	155	74%	1e-19	23.91%	525	Query_97052
Chgr_CD4		68.6	147	66%	9e-16	23.89%	535	Query_97048
Gici_CD4		62.8	163	69%	5e-14	23.69%	532	Query_97050
Gici_LAG-3		54.3	169	60%	2e-11	24.31%	488	Query_97051
Scto_LAG-3		51.6	145	59%	2e-10	20.99%	480	Query_97055
Scca_LAG-3		50.4	137	74%	4e-10	20.09%	474	Query_97053
Heze_LAG-3		42.7	143	63%	1e-07	23.26%	489	Query_97057
Chgr_LAG-3		32.3	139	53%	2e-04	22.87%	486	Query_97049

LAG-3

Descriptions | Graphic Summary | Alignments

Sequences producing significant alignments — Download — Select columns — Show 100

select all *10 sequences selected* — Graphics | Distance tree of results | Multiple alignment | MSA Viewer

Description	Scientific Name	Max Score	Total Score	Query Cover	E value	Per. Ident	Acc. Len	Accession
Gici_LAG-3		125	125	84%	2e-34	24.65%	488	Query_27261
Scto_LAG-3		112	132	95%	5e-30	24.34%	480	Query_27265
Scca_LAG-3		102	131	91%	7e-27	23.67%	474	Query_27263
Heze_LAG-3		95.5	138	95%	2e-24	22.11%	489	Query_27267
Scto_CD4		95.9	110	91%	2e-24	23.93%	525	Query_27264
Chgr_LAG-3		90.1	113	91%	2e-22	22.82%	486	Query_27259
Heze_CD4		87.8	193	80%	9e-22	24.13%	533	Query_27266
Scca_CD4		84.3	123	80%	1e-20	22.27%	525	Query_27262
Gici_CD4		81.3	113	72%	1e-19	23.66%	532	Query_27260
Chgr_CD4		76.3	76.3	63%	4e-18	22.80%	535	Query_27258

Acipenser ruthenus (sterlet sturgeon)

CD4-1

Descriptions | Graphic Summary | Alignments

Sequences producing significant alignments — Download — Select columns — Show 100

select all *10 sequences selected* — Graphics | Distance tree of results | Multiple alignment | MSA Viewer

Description	Scientific Name	Max Score	Total Score	Query Cover	E value	Per. Ident	Acc. Len	Accession
Scto_CD4		125	125	86%	2e-34	25.68%	525	Query_64420
Scca_CD4		114	114	93%	1e-30	24.20%	525	Query_64418
Heze_CD4		111	192	89%	2e-29	22.88%	533	Query_64422
Gici_CD4		97.4	174	90%	6e-25	22.70%	532	Query_64416
Chgr_CD4		96.7	169	76%	1e-24	22.49%	535	Query_64414
Scca_LAG-3		82.4	82.4	81%	4e-20	23.06%	474	Query_64419
Scto_LAG-3		82.0	133	83%	5e-20	21.10%	480	Query_64421
Heze_LAG-3		76.6	165	88%	3e-18	21.97%	489	Query_64423
Gici_LAG-3		47.4	183	72%	6e-09	22.44%	488	Query_64417
Chgr_LAG-3		34.7	125	64%	5e-05	19.59%	486	Query_64415

CD4-2

Descriptions | Graphic Summary | Alignments

Sequences producing significant alignments — Download — Select columns — Show 100

select all *10 sequences selected* — Graphics | Distance tree of results | Multiple alignment | MSA Viewer

Description	Scientific Name	Max Score	Total Score	Query Cover	E value	Per. Ident	Acc. Len	Accession
Scto_CD4		65.5	115	81%	1e-14	21.17%	525	Query_12998
Scca_CD4		60.5	122	59%	4e-13	20.52%	525	Query_12996
Chgr_CD4		56.6	103	75%	7e-12	20.10%	535	Query_12992
Gici_CD4		47.0	104	88%	7e-09	20.52%	532	Query_12994
Heze_CD4		45.4	64.3	71%	3e-08	20.46%	533	Query_13000
Heze_LAG-3		42.7	192	52%	2e-07	21.25%	489	Query_13001
Scto_LAG-3		37.7	64.3	86%	6e-06	20.37%	480	Query_12999
Gici_LAG-3		34.7	113	49%	5e-05	25.00%	488	Query_12995
Scca_LAG-3		32.0	61.2	61%	4e-04	21.73%	474	Query_12997
Chgr_LAG-3		25.4	131	46%	0.041	25.53%	486	Query_12993

LAG-3

Descriptions | Graphic Summary | Alignments

Sequences producing significant alignments Download Select columns Show 100

select all 10 sequences selected Graphics Distance tree of results Multiple alignment MSA Viewer

Description	Scientific Name	Max Score	Total Score	Query Cover	E value	Per. Ident	Acc. Len	Accession
Gici_LAG-3		135	135	84%	7e-38	28.19%	488	Query_201185
Scca_LAG-3		131	131	92%	9e-37	28.60%	474	Query_201187
Scto_LAG-3		131	131	94%	1e-36	26.48%	480	Query_201189
Heze_CD4		123	199	80%	1e-33	28.65%	533	Query_201190
Scto_CD4		120	196	89%	1e-32	24.40%	525	Query_201188
Heze_LAG-3		116	116	84%	2e-31	26.00%	489	Query_201191
Chgr_CD4		103	125	90%	5e-27	24.27%	535	Query_201182
Scca_CD4		102	180	83%	1e-26	23.64%	525	Query_201186
Chgr_LAG-3		90.1	109	79%	1e-22	24.65%	486	Query_201183
Gici_CD4		85.5	204	83%	5e-21	23.30%	532	Query_201184

Polyodon spathula **(Mississippi paddlefish)**

CD4-1

Descriptions | Graphic Summary | Alignments

Sequences producing significant alignments Download Select columns Show 100

select all 10 sequences selected Graphics Distance tree of results Multiple alignment MSA Viewer

Description	Scientific Name	Max Score	Total Score	Query Cover	E value	Per. Ident	Acc. Len	Accession
Scca_CD4		120	120	88%	1e-32	23.45%	525	Query_238204
Scto_CD4		111	111	87%	1e-29	24.28%	525	Query_238206
Heze_CD4		110	186	80%	4e-29	23.79%	533	Query_238208
Chgr_CD4		99.8	114	90%	1e-25	21.93%	535	Query_238200
Gici_CD4		97.4	182	88%	7e-25	23.80%	532	Query_238202
Scto_LAG-3		79.0	185	83%	5e-19	21.08%	480	Query_238207
Scca_LAG-3		78.2	124	79%	1e-18	22.28%	474	Query_238205
Heze_LAG-3		70.1	180	86%	3e-16	22.79%	489	Query_238209
Gici_LAG-3		50.4	70.5	71%	6e-10	21.83%	488	Query_238203
Chgr_LAG-3		40.0	140	85%	1e-06	28.00%	486	Query_238201

CD4-2

Descriptions | Graphic Summary | Alignments

Sequences producing significant alignments Download Select columns Show 100

select all 10 sequences selected Graphics Distance tree of results Multiple alignment MSA Viewer

Description	Scientific Name	Max Score	Total Score	Query Cover	E value	Per. Ident	Acc. Len	Accession
Scto_CD4		65.1	102	74%	1e-14	22.29%	525	Query_420598
Chgr_CD4		62.4	122	75%	1e-13	22.05%	535	Query_420592
Scca_CD4		57.0	57.0	81%	6e-12	20.05%	525	Query_420596
Heze_CD4		53.9	99.3	65%	5e-11	23.53%	533	Query_420600
Gici_CD4		51.6	99.3	61%	3e-10	21.41%	532	Query_420594
Scto_LAG-3		51.2	80.5	86%	3e-10	20.98%	480	Query_420599
Scca_LAG-3		47.4	77.8	86%	5e-09	20.68%	474	Query_420597
Heze_LAG-3		42.4	118	67%	2e-07	33.67%	489	Query_420601
Gici_LAG-3		32.7	109	68%	2e-04	23.24%	488	Query_420595
Chgr_LAG-3		30.4	158	39%	0.001	24.43%	486	Query_420593

LAG-3

Descriptions Graphic Summary Alignments

Sequences producing significant alignments Download Select columns Show 100

select all *10 sequences selected* Graphics Distance tree of results Multiple alignment MSA Viewer

Description	Scientific Name	Max Score	Total Score	Query Cover	E value	Per. Ident	Acc. Len	Accession
Scto_LAG-3		144	144	95%	2e-41	27.81%	480	Query_1887
Scca_LAG-3		141	141	92%	3e-40	30.48%	474	Query_1885
Gici_LAG-3		136	136	83%	3e-38	27.60%	488	Query_1883
Heze_CD4		132	175	65%	8e-37	28.32%	533	Query_1888
Heze_LAG-3		129	129	87%	1e-35	26.08%	489	Query_1889
Scto_CD4		114	194	78%	1e-30	25.91%	525	Query_1886
Scca_CD4		100	201	78%	6e-26	22.70%	525	Query_1884
Gici_CD4		100	178	76%	8e-26	24.78%	532	Query_1882
Chgr_LAG-3		98.2	98.2	84%	3e-25	24.72%	486	Query_1881
Chgr_CD4		97.8	97.8	72%	5e-25	24.48%	535	Query_1880

Lepisosteus oculatus **(spotted gar)**

CD4-1

Descriptions Graphic Summary Alignments

Sequences producing significant alignments Download Select columns Show 100

select all *10 sequences selected* Graphics Distance tree of results Multiple alignment MSA Viewer

Description	Scientific Name	Max Score	Total Score	Query Cover	E value	Per. Ident	Acc. Len	Accession
Heze_CD4		67.4	129	70%	3e-15	21.39%	533	Query_39660
Gici_CD4		64.3	80.5	74%	3e-14	22.79%	532	Query_39654
Scto_CD4		58.9	73.5	86%	1e-12	20.64%	525	Query_39658
Chgr_CD4		58.5	125	77%	2e-12	32.41%	535	Query_39652
Scca_CD4		57.0	83.2	75%	5e-12	18.83%	525	Query_39656
Heze_LAG-3		44.7	118	70%	4e-08	20.23%	489	Query_39661
Scto_LAG-3		44.3	97.8	88%	5e-08	20.59%	480	Query_39659
Chgr_LAG-3		39.3	147	80%	2e-06	22.95%	486	Query_39653
Gici_LAG-3		38.5	128	55%	3e-06	24.24%	488	Query_39655
Scca_LAG-3		35.8	139	71%	2e-05	20.89%	474	Query_39657

CD4-2

Descriptions Graphic Summary Alignments

Sequences producing significant alignments Download Select columns Show 100

select all *10 sequences selected* Graphics Distance tree of results Multiple alignment MSA Viewer

Description	Scientific Name	Max Score	Total Score	Query Cover	E value	Per. Ident	Acc. Len	Accession
Scca_CD4		79.7	158	75%	2e-19	24.52%	525	Query_64654
Heze_CD4		77.8	116	75%	9e-19	24.75%	533	Query_64658
Scto_CD4		69.7	200	74%	3e-16	23.59%	525	Query_64656
Gici_CD4		67.0	166	61%	2e-15	25.76%	532	Query_64652
Chgr_CD4		58.9	149	72%	1e-12	23.55%	535	Query_64650
Heze_LAG-3		48.1	137	58%	3e-09	26.24%	489	Query_64659
Scto_LAG-3		45.1	126	65%	3e-08	26.05%	480	Query_64657
Scca_LAG-3		43.5	133	71%	7e-08	26.79%	474	Query_64655
Gici_LAG-3		38.1	120	41%	3e-06	24.21%	488	Query_64653
Chgr_LAG-3		25.4	123	63%	0.029	25.23%	486	Query_64651

LAG-3

Descriptions | Graphic Summary | Alignments

Sequences producing significant alignments Download Select columns Show 100

select all *10 sequences selected* Graphics Distance tree of results Multiple alignment MSA Viewer

Description	Scientific Name	Max Score	Total Score	Query Cover	E value	Per. Ident	Acc. Len	Accession
Scca_LAG-3		99.4	99.4	90%	1e-25	26.22%	474	Query_46243
Gici_LAG-3		90.5	120	66%	9e-23	27.83%	488	Query_46241
Scto_LAG-3		82.4	97.0	76%	3e-20	27.37%	480	Query_46245
Heze_LAG-3		80.9	95.9	66%	1e-19	26.29%	489	Query_46247
Scto_CD4		79.3	105	86%	5e-19	23.13%	525	Query_46244
Scca_CD4		76.6	97.0	86%	3e-18	23.08%	525	Query_46242
Heze_CD4		75.9	134	79%	5e-18	23.46%	533	Query_46246
Gici_CD4		75.5	75.5	65%	7e-18	25.46%	532	Query_46240
Chgr_CD4		62.8	83.9	85%	8e-14	24.66%	535	Query_46238
Chgr_LAG-3		49.7	49.7	55%	1e-09	25.78%	486	Query_46239

Danio rerio (zebrafish)

CD4-1

Descriptions | Graphic Summary | Alignments

Sequences producing significant alignments Download Select columns Show 100

select all *10 sequences selected* Graphics Distance tree of results Multiple alignment MSA Viewer

Description	Scientific Name	Max Score	Total Score	Query Cover	E value	Per. Ident	Acc. Len	Accession
Scto_CD4		76.3	91.6	76%	4e-18	22.87%	525	Query_18272
Scca_CD4		72.8	72.8	76%	5e-17	21.87%	525	Query_18270
Heze_CD4		50.1	95.5	71%	8e-10	23.55%	533	Query_18274
Gici_CD4		46.2	61.2	77%	1e-08	20.76%	532	Query_18268
Chgr_CD4		42.7	75.5	77%	2e-07	19.57%	535	Query_18266
Gici_LAG-3		42.0	68.2	71%	2e-07	21.23%	488	Query_18269
Scca_LAG-3		39.7	59.7	84%	2e-06	18.92%	474	Query_18271
Scto_LAG-3		38.1	183	81%	4e-06	22.93%	480	Query_18273
Chgr_LAG-3		32.0	117	47%	4e-04	25.00%	486	Query_18267
Heze_LAG-3		26.6	128	62%	0.018	20.95%	489	Query_18275

CD4-2.1

Descriptions | Graphic Summary | Alignments

Sequences producing significant alignments Download Select columns Show 100

select all *10 sequences selected* Graphics Distance tree of results Multiple alignment MSA Viewer

Description	Scientific Name	Max Score	Total Score	Query Cover	E value	Per. Ident	Acc. Len	Accession
Chgr_CD4		47.8	92.0	66%	3e-09	23.79%	535	Query_19106
Scca_CD4		45.4	175	78%	2e-08	26.14%	525	Query_19110
Heze_LAG-3		39.7	187	40%	1e-06	27.35%	489	Query_19115
Scto_CD4		39.7	132	79%	1e-06	21.56%	525	Query_19112
Heze_CD4		39.3	143	62%	1e-06	23.96%	533	Query_19114
Scto_LAG-3		38.5	106	44%	3e-06	23.21%	480	Query_19113
Gici_CD4		38.1	76.2	48%	4e-06	24.46%	532	Query_19108
Scca_LAG-3		32.0	126	47%	3e-04	25.60%	474	Query_19111
Chgr_LAG-3		24.3	99.3	33%	0.079	31.03%	486	Query_19107
Gici_LAG-3		23.5	127	61%	0.15	23.08%	488	Query_19109

LAG-3

Descriptions | Graphic Summary | Alignments

Sequences producing significant alignments Download Select columns Show 100

select all *10 sequences selected* Graphics Distance tree of results Multiple alignment MSA Viewer

Description	Scientific Name	Max Score	Total Score	Query Cover	E value	Per. Ident	Acc. Len	Accession
Gici_LAG-3		89.4	152	67%	2e-22	25.74%	488	Query_39553
Scto_CD4		80.5	152	74%	1e-19	24.72%	525	Query_39556
Scca_LAG-3		79.3	94.0	91%	3e-19	22.47%	474	Query_39555
Scca_CD4		78.2	202	83%	1e-18	26.21%	525	Query_39554
Chgr_LAG-3		75.9	97.0	78%	5e-18	25.66%	486	Query_39551
Gici_CD4		75.9	178	75%	5e-18	25.55%	532	Query_39552
Heze_LAG-3		70.9	70.9	63%	2e-16	23.60%	489	Query_39559
Chgr_CD4		69.3	209	88%	6e-16	24.59%	535	Query_39550
Scto_LAG-3		68.6	100	79%	1e-15	21.71%	480	Query_39557
Heze_CD4		63.5	227	76%	4e-14	24.09%	533	Query_39558

Ictalurus punctatus **(channel catfish)**

CD4-1

Descriptions | Graphic Summary | Alignments

Sequences producing significant alignments Download Select columns Show 100

select all *10 sequences selected* Graphics Distance tree of results Multiple alignment MSA Viewer

Description	Scientific Name	Max Score	Total Score	Query Cover	E value	Per. Ident	Acc. Len	Accession
Scto_CD4		100	144	76%	5e-26	24.73%	525	Query_38378
Scca_CD4		94.4	94.4	75%	6e-24	24.73%	525	Query_38376
Heze_CD4		89.7	110	74%	2e-22	25.49%	533	Query_38380
Chgr_CD4		85.1	140	99%	5e-21	24.54%	535	Query_38372
Gici_CD4		82.8	116	81%	3e-20	22.25%	532	Query_38374
Scto_LAG-3		51.6	71.2	87%	2e-10	20.77%	480	Query_38379
Heze_LAG-3		47.8	137	70%	4e-09	22.45%	489	Query_38381
Chgr_LAG-3		44.7	165	62%	4e-08	26.89%	486	Query_38373
Scca_LAG-3		43.5	102	72%	8e-08	30.36%	474	Query_38377
Gici_LAG-3		42.7	65.8	84%	2e-07	21.56%	488	Query_38375

CD4-2

Descriptions | Graphic Summary | Alignments

Sequences producing significant alignments Download Select columns Show 100

select all *10 sequences selected* Graphics Distance tree of results Multiple alignment MSA Viewer

Description	Scientific Name	Max Score	Total Score	Query Cover	E value	Per. Ident	Acc. Len	Accession
Scca_CD4		51.6	164	91%	2e-10	25.00%	525	Query_30938
Gici_CD4		50.4	81.2	57%	5e-10	23.51%	532	Query_30936
Scto_CD4		48.5	129	88%	2e-09	24.00%	525	Query_30940
Chgr_CD4		48.1	142	70%	3e-09	22.56%	535	Query_30934
Scto_LAG-3		45.1	72.4	41%	2e-08	24.71%	480	Query_30941
Heze_CD4		42.7	137	68%	1e-07	20.27%	533	Query_30942
Gici_LAG-3		42.4	151	68%	1e-07	21.96%	488	Query_30937
Scca_LAG-3		40.8	75.9	67%	5e-07	23.95%	474	Query_30939
Heze_LAG-3		29.6	205	71%	0.001	29.09%	489	Query_30943
Chgr_LAG-3		28.9	74.3	61%	0.003	19.65%	486	Query_30935

LAG-3

Description	Max Score	Total Score	Query Cover	E value	Per. Ident	Acc. Len	Accession
Gici_LAG-3	82.8	109	64%	3e-20	25.37%	488	Query_4679
Heze_CD4	75.9	155	85%	7e-18	25.29%	533	Query_4684
Chgr_CD4	63.9	123	79%	4e-14	25.07%	535	Query_4676
Heze_LAG-3	62.8	115	61%	9e-14	22.62%	489	Query_4685
Scto_LAG-3	62.4	79.3	80%	1e-13	22.45%	480	Query_4683
Scca_LAG-3	57.8	57.8	87%	3e-12	20.77%	474	Query_4681
Gici_CD4	57.8	111	68%	3e-12	22.55%	532	Query_4678
Chgr_LAG-3	55.8	105	63%	1e-11	23.50%	486	Query_4677
Scto_CD4	53.1	68.2	68%	1e-10	23.42%	525	Query_4682
Scca_CD4	47.8	93.2	85%	5e-09	22.05%	525	Query_4680

Oncorhynchus mykiss (rainbow trout)

CD4-1

Description	Max Score	Total Score	Query Cover	E value	Per. Ident	Acc. Len	Accession
Scca_CD4	82.4	142	78%	4e-20	22.22%	525	Query_56620
Scca_LAG-3	79.3	79.3	80%	3e-19	21.27%	474	Query_56621
Chgr_CD4	79.0	125	82%	6e-19	25.98%	535	Query_56616
Scto_CD4	78.6	78.6	80%	9e-19	21.09%	525	Query_56622
Scto_LAG-3	77.8	108	85%	1e-18	22.30%	480	Query_56623
Gici_LAG-3	75.5	113	80%	7e-18	20.75%	488	Query_56619
Heze_CD4	68.9	107	91%	9e-16	21.34%	533	Query_56624
Gici_CD4	65.1	147	78%	2e-14	23.06%	532	Query_56618
Chgr_LAG-3	60.8	117	77%	3e-13	22.65%	486	Query_56617
Heze_LAG-3	59.7	102	71%	7e-13	20.06%	489	Query_56625

CD4-2a

Description	Max Score	Total Score	Query Cover	E value	Per. Ident	Acc. Len	Accession
Scto_CD4	53.5	108	58%	3e-11	23.71%	525	Query_41228
Scca_CD4	50.4	107	55%	3e-10	25.00%	525	Query_41226
Heze_LAG-3	42.0	112	53%	1e-07	24.43%	489	Query_41231
Heze_CD4	39.3	127	58%	9e-07	22.83%	533	Query_41230
Gici_CD4	39.3	145	76%	1e-06	22.91%	532	Query_41224
Scto_LAG-3	37.4	115	53%	4e-06	24.68%	480	Query_41229
Chgr_LAG-3	35.4	124	61%	2e-05	25.24%	486	Query_41223
Chgr_CD4	35.0	105	50%	2e-05	21.26%	535	Query_41222
Scca_LAG-3	28.1	92.8	62%	0.004	23.50%	474	Query_41227
Gici_LAG-3	26.6	100	66%	0.011	25.88%	488	Query_41225

CD4-2b

Descriptions | Graphic Summary | Alignments

Sequences producing significant alignments Download Select columns Show 100

select all *10 sequences selected* Graphics Distance tree of results Multiple alignment MSA Viewer

	Description	Scientific Name	Max Score	Total Score	Query Cover	E value	Per. Ident	Acc. Len	Accession
☑	Scto_CD4		52.4	127	49%	7e-11	27.14%	525	Query_36756
☑	Scca_CD4		48.1	122	42%	2e-09	30.70%	525	Query_36754
☑	Gici_CD4		37.4	136	74%	5e-06	23.26%	532	Query_36752
☑	Heze_LAG-3		36.2	83.9	47%	9e-06	25.79%	489	Query_36759
☑	Scto_LAG-3		35.4	130	59%	2e-05	26.76%	480	Query_36757
☑	Chgr_CD4		35.0	86.6	65%	2e-05	25.21%	535	Query_36750
☑	Heze_CD4		32.7	106	44%	1e-04	21.74%	533	Query_36758
☑	Chgr_LAG-3		32.0	112	53%	2e-04	25.00%	486	Query_36751
☑	Scca_LAG-3		30.0	92.8	47%	9e-04	21.51%	474	Query_36755
☑	Gici_LAG-3		28.9	87.8	63%	0.002	22.73%	488	Query_36753

LAG-3

Descriptions | Graphic Summary | Alignments

Sequences producing significant alignments Download Select columns Show 100

select all *10 sequences selected* Graphics Distance tree of results Multiple alignment MSA Viewer

	Description	Scientific Name	Max Score	Total Score	Query Cover	E value	Per. Ident	Acc. Len	Accession
☑	Heze_CD4		88.2	150	77%	6e-22	27.93%	533	Query_42678
☑	Scto_CD4		87.0	121	86%	2e-21	23.32%	525	Query_42676
☑	Heze_LAG-3		85.9	85.9	67%	3e-21	25.00%	489	Query_42679
☑	Scca_LAG-3		79.3	79.3	90%	4e-19	23.54%	474	Query_42675
☑	Chgr_CD4		77.0	216	88%	2e-18	24.93%	535	Query_42670
☑	Gici_CD4		76.3	120	72%	4e-18	25.14%	532	Query_42672
☑	Scca_CD4		71.2	124	85%	2e-16	21.92%	525	Query_42674
☑	Scto_LAG-3		70.1	70.1	83%	3e-16	23.02%	480	Query_42677
☑	Gici_LAG-3		68.9	68.9	78%	9e-16	24.88%	488	Query_42673
☑	Chgr_LAG-3		64.3	83.9	82%	2e-14	23.60%	486	Query_42671

Takifugu rubripes (fugu)

CD4-1

Descriptions | Graphic Summary | Alignments

Sequences producing significant alignments Download Select columns Show 100

select all *10 sequences selected* Graphics Distance tree of results Multiple alignment MSA Viewer

	Description	Scientific Name	Max Score	Total Score	Query Cover	E value	Per. Ident	Acc. Len	Accession
☑	Scca_CD4		70.1	86.6	93%	4e-16	24.73%	525	Query_44350
☑	Scto_CD4		68.2	68.2	91%	1e-15	22.56%	525	Query_44352
☑	Scca_LAG-3		64.7	64.7	70%	1e-14	23.26%	474	Query_44351
☑	Chgr_CD4		63.5	118	78%	4e-14	24.60%	535	Query_44346
☑	Gici_CD4		55.8	72.8	92%	1e-11	22.29%	532	Query_44348
☑	Heze_CD4		50.8	50.8	96%	4e-10	23.17%	533	Query_44354
☑	Scto_LAG-3		48.9	87.4	68%	2e-09	22.42%	480	Query_44353
☑	Gici_LAG-3		45.1	90.9	82%	3e-08	22.99%	488	Query_44349
☑	Heze_LAG-3		44.7	102	75%	4e-08	22.92%	489	Query_44355
☑	Chgr_LAG-3		40.4	131	30%	8e-07	29.73%	486	Query_44347

CD4-2

Descriptions | Graphic Summary | Alignments

Sequences producing significant alignments Download Select columns Show 100

select all *10 sequences selected* Graphics Distance tree of results Multiple alignment MSA Viewer

Description	Scientific Name	Max Score	Total Score	Query Cover	E value	Per. Ident	Acc. Len	Accession
Scca_CD4		44.7	118	66%	2e-08	23.32%	525	Query_49208
Heze_LAG-3		41.6	164	66%	2e-07	22.10%	489	Query_49213
Scto_CD4		41.2	141	75%	2e-07	25.17%	525	Query_49210
Scto_LAG-3		38.1	148	70%	2e-06	21.19%	480	Query_49211
Chgr_LAG-3		36.6	168	67%	7e-06	24.72%	486	Query_49205
Gici_CD4		36.2	105	62%	1e-05	25.00%	532	Query_49206
Scca_LAG-3		34.3	143	66%	3e-05	22.46%	474	Query_49209
Chgr_CD4		31.2	131	69%	4e-04	24.40%	535	Query_49204
Gici_LAG-3		30.4	110	60%	6e-04	22.68%	488	Query_49207
Heze_CD4		27.3	141	51%	0.006	21.78%	533	Query_49212

LAG-3

Descriptions | Graphic Summary | Alignments

Sequences producing significant alignments Download Select columns Show 100

select all *10 sequences selected* Graphics Distance tree of results Multiple alignment MSA Viewer

Description	Scientific Name	Max Score	Total Score	Query Cover	E value	Per. Ident	Acc. Len	Accession
Chgr_LAG-3		80.1	98.6	91%	2e-19	26.03%	486	Query_39591
Scto_LAG-3		76.6	107	69%	2e-18	21.98%	480	Query_39597
Gici_LAG-3		75.5	103	72%	6e-18	25.21%	488	Query_39593
Heze_LAG-3		73.9	73.9	79%	2e-17	22.07%	489	Query_39599
Scto_CD4		73.6	73.6	88%	3e-17	22.20%	525	Query_39596
Chgr_CD4		71.2	124	75%	2e-16	24.07%	535	Query_39590
Scca_CD4		68.9	145	86%	9e-16	22.58%	525	Query_39594
Scca_LAG-3		65.5	80.5	73%	1e-14	21.25%	474	Query_39595
Heze_CD4		63.2	92.0	80%	6e-14	23.22%	533	Query_39598
Gici_CD4		60.8	95.1	66%	3e-13	22.94%	532	Query_39592

Latimeria menadoensis (Menado coelacanth)

CD4

Descriptions | Graphic Summary | Alignments

Sequences producing significant alignments Download Select columns Show 100

select all *10 sequences selected* Graphics Distance tree of results Multiple alignment MSA Viewer

Description	Scientific Name	Max Score	Total Score	Query Cover	E value	Per. Ident	Acc. Len	Accession
Scto_CD4		99.8	99.8	85%	1e-25	21.80%	525	Query_8942
Heze_CD4		99.8	155	81%	1e-25	24.59%	533	Query_8944
Scca_CD4		98.2	98.2	85%	4e-25	21.88%	525	Query_8940
Chgr_CD4		89.0	203	93%	4e-22	22.37%	535	Query_8936
Gici_CD4		80.9	189	77%	2e-19	22.39%	532	Query_8938
Scca_LAG-3		75.9	75.9	79%	5e-18	21.29%	474	Query_8941
Heze_LAG-3		70.1	118	63%	4e-16	20.91%	489	Query_8945
Scto_LAG-3		65.1	65.1	79%	1e-14	21.19%	480	Query_8943
Gici_LAG-3		57.4	132	65%	4e-12	21.25%	488	Query_8939
Chgr_LAG-3		39.7	160	69%	2e-06	23.14%	486	Query_8937

Latimeria chalumnae (West Indian Ocean coelacanth)

LAG-3

Descriptions | Graphic Summary | Alignments

Sequences producing significant alignments Download Select columns Show 100

select all *10 sequences selected* Graphics Distance tree of results Multiple alignment MSA Viewer

	Description	Scientific Name	Max Score	Total Score	Query Cover	E value	Per. Ident	Acc. Len	Accession
☑	Heze_LAG-3		162	162	92%	2e-47	27.59%	489	Query_58815
☑	Scto_LAG-3		144	144	97%	3e-41	25.75%	480	Query_58813
☑	Scca_LAG-3		139	156	99%	3e-39	25.25%	474	Query_58811
☑	Gici_LAG-3		127	127	81%	3e-35	25.29%	488	Query_58809
☑	Chgr_LAG-3		123	123	96%	1e-33	25.39%	486	Query_58807
☑	Heze_CD4		119	172	66%	3e-32	26.61%	533	Query_58814
☑	Scca_CD4		114	227	83%	1e-30	25.54%	525	Query_58810
☑	Scto_CD4		111	240	83%	2e-29	25.40%	525	Query_58812
☑	Gici_CD4		95.5	110	75%	3e-24	22.66%	532	Query_58808
☑	Chgr_CD4		91.3	189	87%	8e-23	24.59%	535	Query_58806

Protopterus annectens (West African Lungfish)

CD4

Descriptions | Graphic Summary | Alignments

Sequences producing significant alignments Download Select columns Show 100

select all *10 sequences selected* Graphics Distance tree of results Multiple alignment MSA Viewer

	Description	Scientific Name	Max Score	Total Score	Query Cover	E value	Per. Ident	Acc. Len	Accession
☑	Heze_CD4		96.3	96.3	84%	1e-24	24.83%	533	Query_50584
☑	Scto_CD4		95.9	153	84%	2e-24	24.59%	525	Query_50582
☑	Chgr_CD4		92.0	92.0	86%	3e-23	23.99%	535	Query_50576
☑	Scca_CD4		87.8	87.8	85%	8e-22	23.82%	525	Query_50580
☑	Scto_LAG-3		77.4	111	80%	2e-18	21.45%	480	Query_50583
☑	Gici_LAG-3		72.8	72.8	83%	5e-17	22.57%	488	Query_50579
☑	Gici_CD4		72.0	131	74%	1e-16	21.12%	532	Query_50578
☑	Heze_LAG-3		64.3	64.3	79%	2e-14	21.65%	489	Query_50585
☑	Chgr_LAG-3		60.5	76.6	83%	5e-13	22.91%	486	Query_50577
☑	Scca_LAG-3		57.8	72.4	79%	2e-12	20.39%	474	Query_50581

LAG-3

Descriptions | Graphic Summary | Alignments

Sequences producing significant alignments Download Select columns Show 100

select all *10 sequences selected* Graphics Distance tree of results Multiple alignment MSA Viewer

	Description	Scientific Name	Max Score	Total Score	Query Cover	E value	Per. Ident	Acc. Len	Accession
☑	Heze_LAG-3		141	141	79%	6e-40	28.71%	489	Query_59515
☑	Gici_LAG-3		139	139	83%	4e-39	27.69%	488	Query_59509
☑	Scto_LAG-3		133	155	93%	4e-37	27.66%	480	Query_59513
☑	Scca_LAG-3		131	131	92%	1e-36	28.13%	474	Query_59511
☑	Chgr_LAG-3		126	141	92%	1e-34	27.61%	486	Query_59507
☑	Scto_CD4		123	138	87%	2e-33	24.29%	525	Query_59512
☑	Scca_CD4		121	166	86%	8e-33	24.34%	525	Query_59510
☑	Heze_CD4		103	103	79%	1e-26	22.60%	533	Query_59514
☑	Chgr_CD4		88.6	124	88%	5e-22	23.08%	535	Query_59506
☑	Gici_CD4		82.4	106	71%	5e-20	20.38%	532	Query_59508

***Xenopus tropicalis* (tropical clawed frog)**

CD4

Descriptions | Graphic Summary | Alignments

Sequences producing significant alignments — Download — Select columns — Show 100

select all *10 sequences selected* — Graphics | Distance tree of results | Multiple alignment | MSA Viewer

Description	Scientific Name	Max Score	Total Score	Query Cover	E value	Per. Ident	Acc. Len	Accession
Scca_CD4		57.4	140	88%	4e-12	22.53%	525	Query_105430
Scto_CD4		54.3	154	92%	3e-11	23.29%	525	Query_105432
Heze_CD4		52.4	101	77%	1e-10	23.78%	533	Query_105434
Gici_CD4		52.0	86.2	77%	2e-10	22.10%	532	Query_105428
Gici_LAG-3		36.2	94.3	78%	1e-05	21.99%	488	Query_105429
Heze_LAG-3		36.2	105	49%	2e-05	26.24%	489	Query_105435
Chgr_CD4		33.1	121	51%	1e-04	23.97%	535	Query_105426
Chgr_LAG-3		32.3	108	46%	2e-04	29.17%	486	Query_105427
Scca_LAG-3		32.0	93.5	82%	4e-04	32.43%	474	Query_105431
Scto_LAG-3		31.6	90.9	79%	5e-04	22.02%	480	Query_105433

LAG-3

Descriptions | Graphic Summary | Alignments

Sequences producing significant alignments — Download — Select columns — Show 100

select all *10 sequences selected* — Graphics | Distance tree of results | Multiple alignment | MSA Viewer

Description	Scientific Name	Max Score	Total Score	Query Cover	E value	Per. Ident	Acc. Len	Accession
Scca_LAG-3		114	114	98%	8e-31	27.22%	474	Query_33839
Heze_LAG-3		107	191	85%	2e-28	26.05%	489	Query_33843
Scto_LAG-3		102	102	87%	6e-27	26.00%	480	Query_33841
Heze_CD4		100	100	87%	6e-26	25.87%	533	Query_33842
Scto_CD4		96.7	96.7	87%	7e-25	23.74%	525	Query_33840
Gici_CD4		87.4	87.4	87%	7e-22	24.83%	532	Query_33836
Chgr_LAG-3		85.9	85.9	85%	2e-21	25.65%	486	Query_33835
Scca_CD4		85.1	212	85%	4e-21	24.10%	525	Query_33838
Chgr_CD4		80.9	112	89%	1e-19	24.04%	535	Query_33834
Gici_LAG-3		68.2	119	82%	1e-15	25.60%	488	Query_33837

***Chelonia mydas* (green sea turtle)**

CD4

Descriptions | Graphic Summary | Alignments

Sequences producing significant alignments — Download — Select columns — Show 100

select all 10 sequences selected — Graphics — Distance tree of results — Multiple alignment — MSA Viewer

Description	Scientific Name	Max Score	Total Score	Query Cover	E value	Per. Ident	Acc. Len	Accession
Scto_CD4		80.9	118	76%	1e-19	23.02%	525	Query_28250
Gici_CD4		76.3	76.3	86%	4e-18	23.90%	532	Query_28246
Scto_LAG-3		75.5	92.8	86%	6e-18	21.22%	480	Query_28251
Scca_CD4		73.2	115	76%	4e-17	22.17%	525	Query_28248
Chgr_CD4		68.6	90.1	83%	1e-15	21.28%	535	Query_28244
Scca_LAG-3		63.2	79.3	86%	5e-14	20.37%	474	Query_28249
Gici_LAG-3		60.5	60.5	82%	4e-13	21.69%	488	Query_28247
Heze_CD4		50.4	67.0	86%	6e-10	22.93%	533	Query_28252
Heze_LAG-3		45.1	71.6	77%	3e-08	19.84%	489	Query_28253
Chgr_LAG-3		35.0	175	68%	4e-05	23.44%	486	Query_28245

LAG-3

Descriptions | Graphic Summary | Alignments

Sequences producing significant alignments — Download — Select columns — Show 100

select all 10 sequences selected — Graphics — Distance tree of results — Multiple alignment — MSA Viewer

Description	Scientific Name	Max Score	Total Score	Query Cover	E value	Per. Ident	Acc. Len	Accession
Heze_LAG-3		123	157	94%	9e-34	29.45%	489	Query_426299
Scca_CD4		120	175	88%	1e-32	24.50%	525	Query_426294
Scto_CD4		120	145	85%	1e-32	23.74%	525	Query_426296
Scto_LAG-3		114	130	86%	1e-30	28.92%	480	Query_426297
Scca_LAG-3		108	108	89%	1e-28	27.22%	474	Query_426295
Gici_LAG-3		99.8	114	85%	1e-25	27.63%	488	Query_426293
Chgr_LAG-3		94.0	135	77%	8e-24	28.30%	486	Query_426291
Chgr_CD4		93.2	136	85%	2e-23	25.05%	535	Query_426290
Gici_CD4		92.0	177	84%	4e-23	24.81%	532	Query_426292
Heze_CD4		89.7	107	80%	2e-22	22.25%	533	Query_426298

***Gallus gallus* (chicken)**

CD4

Descriptions | Graphic Summary | Alignments

Sequences producing significant alignments — Download — Select columns — Show 100

select all 10 sequences selected — Graphics — Distance tree of results — Multiple alignment — MSA Viewer

Description	Scientific Name	Max Score	Total Score	Query Cover	E value	Per. Ident	Acc. Len	Accession
Chgr_CD4		59.3	110	79%	1e-12	22.81%	535	Query_381146
Scto_CD4		58.5	104	81%	2e-12	22.78%	525	Query_381152
Heze_CD4		50.1	220	86%	7e-10	24.72%	533	Query_381154
Heze_LAG-3		45.4	105	71%	2e-08	19.19%	489	Query_381155
Scca_CD4		44.7	123	87%	4e-08	21.32%	525	Query_381150
Gici_LAG-3		42.7	126	88%	1e-07	19.77%	488	Query_381149
Gici_CD4		41.2	120	70%	4e-07	21.00%	532	Query_381148
Scto_LAG-3		38.5	121	66%	3e-06	18.98%	480	Query_381153
Scca_LAG-3		38.1	106	76%	4e-06	20.06%	474	Query_381151
Chgr_LAG-3		29.6	177	46%	0.002	28.57%	486	Query_381147

LAG-3

Descriptions | Graphic Summary | Alignments

Sequences producing significant alignments Download Select columns Show 100

select all *10 sequences selected* Graphics Distance tree of results Multiple alignment MSA Viewer

Description	Scientific Name	Max Score	Total Score	Query Cover	E value	Per. Ident	Acc. Len	Accession
Scto_CD4		128	128	90%	2e-35	25.58%	525	Query_482102
Scca_CD4		125	125	85%	3e-34	25.33%	525	Query_482100
Heze_LAG-3		110	110	84%	3e-29	27.40%	489	Query_482105
Heze_CD4		110	110	84%	3e-29	24.94%	533	Query_482104
Chgr_CD4		105	162	97%	1e-27	24.69%	535	Query_482096
Chgr_LAG-3		96.7	96.7	77%	9e-25	26.38%	486	Query_482097
Gici_LAG-3		89.4	114	97%	2e-22	27.09%	488	Query_482099
Scca_LAG-3		87.8	87.8	95%	7e-22	25.00%	474	Query_482101
Scto_LAG-3		85.9	100	85%	3e-21	27.07%	480	Query_482103
Gici_CD4		83.2	102	91%	3e-20	23.30%	532	Query_482098

Mus musculus **(mouse)**

CD4

Descriptions | Graphic Summary | Alignments

Sequences producing significant alignments Download Select columns Show 100

select all *10 sequences selected* Graphics Distance tree of results Multiple alignment MSA Viewer

Description	Scientific Name	Max Score	Total Score	Query Cover	E value	Per. Ident	Acc. Len	Accession
Gici_LAG-3		45.8	108	69%	2e-08	23.60%	488	Query_423851
Chgr_CD4		44.3	61.6	71%	5e-08	26.60%	535	Query_423848
Gici_CD4		43.9	79.7	69%	6e-08	24.00%	532	Query_423850
Scca_LAG-3		38.5	91.6	41%	3e-06	27.74%	474	Query_423853
Scto_LAG-3		37.4	88.5	43%	7e-06	24.52%	480	Query_423855
Heze_LAG-3		34.7	65.8	27%	5e-05	21.64%	489	Query_423857
Scto_CD4		34.7	116	49%	5e-05	25.62%	525	Query_423854
Heze_CD4		33.9	136	77%	8e-05	20.25%	533	Query_423856
Scca_CD4		32.3	81.6	54%	3e-04	23.45%	525	Query_423852
Chgr_LAG-3		28.1	99.3	53%	0.005	20.34%	486	Query_423849

LAG-3

Descriptions | Graphic Summary | Alignments

Sequences producing significant alignments Download Select columns Show 100

select all *10 sequences selected* Graphics Distance tree of results Multiple alignment MSA Viewer

	Description	Scientific Name	Max Score	Total Score	Query Cover	E value	Per. Ident	Acc. Len	Accession
☑	Heze_LAG-3		65.5	142	70%	1e-14	29.89%	489	Query_55273
☑	Chgr_CD4		62.4	173	84%	1e-13	26.08%	535	Query_55264
☑	Gici_LAG-3		60.5	94.3	68%	5e-13	28.03%	488	Query_55267
☑	Scca_LAG-3		60.1	104	81%	5e-13	24.56%	474	Query_55269
☑	Scto_LAG-3		57.8	144	78%	3e-12	26.67%	480	Query_55271
☑	Chgr_LAG-3		57.0	108	80%	5e-12	26.29%	486	Query_55265
☑	Scto_CD4		54.7	74.7	85%	3e-11	21.76%	525	Query_55270
☑	Gici_CD4		53.1	161	63%	1e-10	24.06%	532	Query_55266
☑	Heze_CD4		47.0	47.0	80%	9e-09	20.67%	533	Query_55272
☑	Scca_CD4		44.7	171	82%	5e-08	25.77%	525	Query_55268

***Homo sapiens* (human)**

CD4

Descriptions | Graphic Summary | Alignments

Sequences producing significant alignments Download Select columns Show 100

select all *10 sequences selected* Graphics Distance tree of results Multiple alignment MSA Viewer

	Description	Scientific Name	Max Score	Total Score	Query Cover	E value	Per. Ident	Acc. Len	Accession
☑	Heze_CD4		56.6	102	45%	6e-12	26.92%	533	Query_43442
☑	Gici_CD4		55.5	89.7	51%	2e-11	29.21%	532	Query_43436
☑	Scto_CD4		51.6	125	87%	2e-10	25.13%	525	Query_43440
☑	Chgr_CD4		49.3	148	63%	1e-09	25.34%	535	Query_43434
☑	Scca_CD4		46.6	176	86%	8e-09	25.21%	525	Query_43438
☑	Gici_LAG-3		43.9	133	60%	5e-08	21.83%	488	Query_43437
☑	Scto_LAG-3		43.5	148	36%	8e-08	26.12%	480	Query_43441
☑	Heze_LAG-3		37.4	115	57%	7e-06	22.22%	489	Query_43443
☑	Scca_LAG-3		34.7	131	49%	4e-05	25.00%	474	Query_43439
☑	Chgr_LAG-3		32.0	140	60%	3e-04	19.70%	486	Query_43435

LAG-3

Descriptions | Graphic Summary | Alignments

Sequences producing significant alignments Download Select columns Show 100

select all *10 sequences selected* Graphics Distance tree of results Multiple alignment MSA Viewer

	Description	Scientific Name	Max Score	Total Score	Query Cover	E value	Per. Ident	Acc. Len	Accession
☑	Heze_LAG-3		74.3	74.3	76%	2e-17	26.48%	489	Query_116417
☑	Gici_CD4		68.9	112	68%	1e-15	24.55%	532	Query_116410
☑	Chgr_CD4		64.7	130	76%	2e-14	24.41%	535	Query_116408
☑	Gici_LAG-3		59.3	108	59%	1e-12	26.51%	488	Query_116411
☑	Scto_CD4		58.9	132	74%	1e-12	22.14%	525	Query_116414
☑	Scca_LAG-3		55.5	115	76%	2e-11	25.21%	474	Query_116413
☑	Chgr_LAG-3		55.5	135	82%	2e-11	26.45%	486	Query_116409
☑	Heze_CD4		52.8	233	78%	1e-10	22.22%	533	Query_116416
☑	Scto_LAG-3		49.3	128	78%	2e-09	23.53%	480	Query_116415
☑	Scca_CD4		44.3	122	82%	5e-08	20.00%	525	Query_116412
