# Supplementary material for: CD4 and LAG-3 from sharks to humans: related molecules with motifs for opposing functions
Source: Front Immunol. 2023 Dec 21;14:1267743. doi: 10.3389/fimmu.2023.1267743 (PMC10768021; doi:10.3389/fimmu.2023.1267743)
Supplement: Supplementary file 7 [file DataSheet_7.pdf]

Supplementary file 7

Alignment of CD8α and CD8β cytoplasmic tails of representative species

This alignment shows the deduced amino acid sequences of the transmembrane and cytoplasmic tail regions of CD8α and CD8β molecules of representative species. It reveals that only from the evolutionary level of lungfishes the CxH motif in CD8α changed to CxC and the CxH motif in CD8β was lost. In many salamanders, here represented by axolotl, the CxC motif in CD8α has been reverted to a CxH motif again, supportive of the two motifs having a similar function (the binding of LCK). This alignment figure focuses on the Cx(C/H) motif and does not address possible splicing variation. The coloring per residue types and the shading of the CxC versus CxH motifs is as in main text Fig. 3, as are the indications of exon border positions with their phases between brackets. Other conservations that we like to highlight, somewhat intuitively, are shaded gray. The database sources for the depicted sequences are explained below the alignment.

```
Nurse shark CD8a       KTEDPLSCHFIFWAPLTGAAALLLIALTSVSIAYCRR(1)--PRRRRCQHQFRKR--(2)PIAEEDR-L--SNRYL
S.z. catshark CD8a     IHQDGLSCAMIIWAPLAGGTLLLLLALILVSIVYCRT(1)--PRRRRCQHQFRKR--(2)PVADEVRRP--PNGYY
Elephant shark CD8a    IADDRFSCHLIIWTSLAGVSSLLLVILVTVSILYCKR(1)--PRRRRCQHQFRKR--(2)PMPEEDRRL--PNNYY
Reedfish CD8a      HDATGFKPGLKCSIAIFAPLAVGCIVLLILLIT-SIAVCNS(1)--IRTRHCPHHYKRR--(2)LRKLNSGQYV
M. paddlefish CD8a    VQRDTRLSCDVFIWAPLCGAAGLLLLILIV-TIIMCSK(1)--VRTRRCPHHYRKR--(2)PQKDMSGRPSVTDRYV
Rainbow trout CD8a    GKVDPTASCELIVWAPLTAGCGFLFLLLII-TVCHCNR(1)--IRTKRCPHHYKRQ--(2)PRMAAPGQQHPIANNRLF
Coelacanth CD8a           SGFPCKLYIWTPLAGASLLLLITLII-TIIVCNS(1)--SRKKRCPHHIRKR--(2)TMPEHKGKPSVPDRYA
W.A. lungfish CD8a   RMSPKDLFACELYIWIPLAAISVLLVISLIITLSVGKKS(1)---RRRICKCKNHYKNR(2)PTPEGHGRPQFPDRYI
Tr. clawed frog CD8a SVPIDLGIQCNIYIWGSLAALCSLLLIALLTTSILLCKR(1)--GRPRRCRCKHA----(2)PVAERNNKPKPPARY
Axolotl CD8a       KILTAESLDFSCSLYILVPMAAVGGVLLIAFLVASSLLCKI(1)-FIRRKRCKHHGKKR--(2)PMNENIPRPNIPSR
Chicken CD8a        TSSENILNLYCEIFIWAPLAGVCLVLLVALIV-TIVLCQK(1)   TRRRRCRCKR-----(2)PPNGKPGAKPCVPTRHI
Mouse CD8a            KGTGLDFACDIYIWAPLAGICVALLLSLII-TLICYHR(1)   SRKRVCKCPR-----(2)PLVRQEGKPRPSEKIV
Human CD8a            HTRGLDFACDIYIWAPLAGTCGVLLLSLVI-TLYCNHR(1)   NRRRVCKCPR-----(2)PVVKSGDKPSLSARYV
Nurse shark CD8b            YACNWSIWVSLA-VCNLMLLTSVIFVVIKHKIQS---KGWRRCPHQLRKR
S.s. catshark CD8b          YMCNWAIWGSLV-ACNLLLLISIVAIVIHRRKRS(1)-SRRRCPHQFQKR
Reedfish CD8b               AYCLPKILGPLL-GTAAVLCILLVCTLYYFSK(1)---LPKKCRHGMIT---(2)KKQIR
M. paddlefish CD8b          FSCGSLVLGPLA-GGAAALLVAVVSTLYYFSR(1)---LPKKCRHRMIK---(2)KKLLQ
Rainbow trout CD8b            CGYKVLWPLV-GVLLTLAVALIYTLYYFSR(1)---LPKKCRHQFAK---(2)KRPMR
Coelacanth CD8b             SLCEIFVWAPLL-GCVVILLIIYVVTVIWFKR(1)---KMRRCPHHFKK
W.A. lungfish CD8b          LPCSSMILCILL-GSIAVLAVLEVAVIICFKN(1)---IRRPYPHYFLKQ
Tr. clawed frog CD8b         MCSTVIWAPLA-GFALILVIVLGATISYTKR(1)---IYRRTRHFYRKQ
Axolotl CD8b                VACSSVIYAPLA-TGLVMLVISLVVMINHLQH(1)--FHRRYRRHFRKQLVK
Chicken CD8b                 ACTPMVWVPLA-AGALLLLLSLIPTIRRFYR(1)--LRRRLWVRAHRR
Mouse CD8b                  LTCSLTTLSLLV-VCILLLLAFL-GVAVYFYC(1)--VRRRARIHFMKQ---(2)FHK
Human CD8b                  PLCSPITLGLLV-AGILVLLVSL-GVAIHLCC(1)--RRRRARLRFMKQ---(2)FYK
```

The sequences compared in the alignment are: Nurse shark (*Ginglymostoma cirratum*) CD8α (GenBank AGN91183) and CD8β (AGQ17914; an intron seems to have been deleted); Small-spotted catshark (*Scyliorhinus canicula*) CD8α (XP_038648937) and CD8β (UBR18749); Elephant

shark (*Callorhinchus milii*) CD8α (XP_007896195); Reedfish (*Erpetoichthys calabaricus*) CD8α (XP_051786116) and CD8β (XP_028660486); Mississippi paddlefish (*Polyodon spathula*) CD8α (XP_041129628) and CD8β (XP_041094710); Rainbow trout (*Oncorhynchus mykiss*) CD8α (NP_001117735) and CD8β (NP_001117480); Coelacanth (*Latimeria chalumnae*) CD8α (XP_006009902, modified with NW_005821155; this GenBank accession is incorrectly annotated as CD8β) and CD8β (deduced from genomic sequence NW_005821155; full-length sequence is MKLVSLFYVISFVITGTDSSIALIQTPSSVLALKDSPAQLTCAMKQNSMDHLGVFWYRQPEGSQEFQFILHATPMN RYTHGENFKDRFNVVRDAFRGSYTLSITSVQLSDNGTYYCVISHTFTLAFGNGTQLNVVNSLPPPPKPTVKPLQKP VKRCGNRDKSIRRTTKTKASLCEIFVWAPLLGCVVILLIIYVVTVIWFKRKMRRCPHHFKK); W.A. lungfish (*Protopterus annectens*) CD8α (XP_043918272) and CD8β (XP_043918273); Tropical clawed frog (*Xenopus tropicalis*) CD8α (XP_031750940) and CD8β (XP_031750941); Axolotl (*Ambystoma mexicanum*) CD8α (GFBM010764279) and CD8β (XP_031750941); Chicken (*Gallus gallus*) CD8α (NP_990566) and CD8β (CAA81258); Mouse (*Mus musculus*) CD8α (NP_001074579) and CD8β (EDK98935); Human (*Homo sapiens*) CD8α (NP_001139345) and CD8β (NP_004922).
