# Supplementary material for: CD4 and LAG-3 from sharks to humans: related molecules with motifs for opposing functions
Source: Front Immunol. 2023 Dec 21;14:1267743. doi: 10.3389/fimmu.2023.1267743 (PMC10768021; doi:10.3389/fimmu.2023.1267743)
Supplement: Supplementary file 8 [file DataSheet_8.pdf]

Supplementary file 8

Alignment of cytoplasmic tails of CD4 in diverse Chondrichthyes

This alignment shows the deduced amino acid sequences of the transmembrane and cytoplasmic tail regions of CD4 molecules of representative Chondrichthyes. It reveals that not only the CxH motif is conserved but also various other residues, which we highlight here, somewhat intuitively, with gray shading. The coloring per residue types and the shading of the CxC versus CxH motifs are as in main text Fig. 3, as are the indications of exon border positions with their phases between brackets. The database sources for the depicted sequences are explained below the alignment.

The CxH motif is bracketed at both sides by basic residues, which is reminiscent of the CxH motif in CD8 (Supplementary file 7). This motif, which considering the CD8 situation is probably sufficient for LCK binding, is separated at its N-terminal side by a proline-rich stretch and several other conserved motifs that may confer additional functions or help bind LCK.

```
Chgr-CD4   PNLGQLGVLSLIQVIGIATGFLTFLLILCVTIVCLIKRSRRR(?)RRALKRLRHPLCREHSYQ(?)LSNQPLCPWSDYSPAERPLPPPPVRYCPHRQPRKGIPPQGRGGR(?)TSSRKGNGGR
Gici-CD4   LTLSEFGDPPLVLIIAASVGAFVLLLLATVIAVCLSKRARRR(?)RRALKRLRHPLCREHSYQ(?)LSNQPLCHSNDYTPGDRPLPPPPIPYCPHRQPRKGRPSHARGSR(?)HARRSQFGP
Scca-CD4   PVVSIFGKDNLILMIGTSASLLLLIVIFTLIGICLAKRARRR(0)RRALRRLRHPLCREHSHQ(2)LSNQPLCNSNDYTSTERPLPPLP-RYCPH-QPRRGRSSQGKGNR(2)LGPRGQYTA
Scto-CD4   PVISIFGKDNLILMIGTSASLLFLIVIFTLIGNCLAKRARRR(0)RQALRRLRHPLCREHSHQ(2)LSNQPLCNSNDYTKTERPLPPLP-RYCPH-QPRRGRSSQGKGNR(2)LGPRGQYIA
Heze-CD4   APHRLFTTGTMIITIGTSAAAVAAILIATLIGIFLAKRARRR(?)RRAVRRLRHPLCREHSHQ(?)LSSQPLYNGNDYILTDRPLPPPPIRYCPHNQPRRGRPSQATSSR(?)RAPKGSYVA
Teca-CD4   AVAILVLLQNNLILFVGLGAGTGVLMVVLVAVTIAVLKRRKK(?)RRRRVVRRPYLCQEHSNQ(?)----PLCQSNDYTDRSRPLPRPPIGRCPHNPPRRGRSPHAKPKR
Cami-CD4                LYYVTPGVLGLLIVILVLSVCSIKHRQTR(?)RRRLRRMKYPLCRVHSNQ(?)LSNQPLCGSNDYVPSHRPLPTPPRMSCPHKQRTTR----------(?)KPRAGAGGRVY
Hyaf-CD4                                                                  (2)LSNQPLCGSNDYSPSHRPLPTPPRGPCPHNQRANR----------(2)
```

The shark CD4 sequences compared in the alignment which are also shown in the main text Fig. 3, and are explained in Supplementary file 3, are for *Chiloscyllium griseum* (Chgr; gray bambooshark), *Ginglymostoma cirratum* (Gici; nurse shark), *Scyliorhinus canicula* (Scca; small-spotted catshark), Scyliorhinus torazame (Scto; cloudy catshark), and *Heterodontus zebra* (Heze; zebra bullhead shark).

Additionally, CD4 sequences are added for *Tetronarce californica* (Pacific electric ray), based on GenBank GFBV01036008 (a TSA sequence), and for the chimaeras *Callorhinchus milii* (Cami; elephant shark), based on overlapping NCBI database single read archive reads (SRAs) (SRR513758.3652837.1, SRR513758.18748387.2, SRR513758.31920741.2, SRR513758.16923920.2, SRR513758.26360757.1, SRR513758.36587725.2, and SRR513758.4218430.1) and *Hydrolagus affinis* (Hyaf; small-eyed rabbitfish), based on the genomic sequence at GenBank JAAILG010093779. In the short JAAILG010093779 scaffold with a *Hyaf-CD4* fragment, we only found information for one of the cytoplasmic tail exons, the last encoded residue being partly speculative (therefore in Italic font). For none of the chimaeras we could find sufficient information to deduce the entire CD4 molecules.
