# Supplementary material for: CD4 and LAG-3 from sharks to humans: related molecules with motifs for opposing functions
Source: Front Immunol. 2023 Dec 21;14:1267743. doi: 10.3389/fimmu.2023.1267743 (PMC10768021; doi:10.3389/fimmu.2023.1267743)
Supplement: Supplementary file 9 [file DataSheet_9.pdf]

Supplementary file 9

Gene Expression analysis

This file shows:

(A; page 2) Expression levels of various genes in cloud catshark (*Scyliorhinus torazame*) were calibrated against not only *EF-1A* (main text Fig. 4), but also against *RPL13*, *ACTB*, and total RNA.

(B; page 5) Northern blot analysis for *CD4*, *CD8A*, and *CD8B* in nurse shark (*Ginglymostoma cirratum*).

(C; page 7) Single nuclei RNA sequencing (snRNA-seq) data analysis of the expression of *CD4*, *LAG-3*, and other immune genes in splenocytes of nurse shark (*Ginglymostoma cirratum*).

(A) Expression levels of various genes in cloud catshark (*Scyliorhinus torazame*) were calibrated against not only *EF-1A* (main text Fig. 4), but also against *RPL13*, *ACTB*, and total RNA

Experiments were done as described for Fig. 4 in the main text, with primers described in Supplementary file 1. Fig. A-I (used as Fig. 4 in the main text) is included here for comparison. Figs. A-II, -III, and -IV show that using *RPL13*, *ACTB*, or total RNA for equilibration did not change the observation that *CD4* expression is highest in the thymus.

Transcript levels of catshark *CD4*, *LAG-3*, *CD3 zeta* (*CD3Z*), *LCK*, *CD8A*, *CD8B*, *T cell receptor alpha* (*TCRA*), *T cell receptor beta* (*TCRB*), and *paired box 5* (*PAX5*) in indicated tissues were analyzed by real-time PCR. A normalized amount of target gene was calculated by dividing the amount of target gene by the amount of *elongation factor 1-alpha* (*EF-1A*) (A-I), *ribosomal protein L13* (*RPL13*) (A-II), actin beta (*ACTB*) (A-III), or total RNA (A-IV). Transcript expression levels of indicated genes were further normalized to those in spleen (set as 1), which are indicated as mean value ± S.D. with individual values (n = 4 fish). One-way ANOVA with Tukey's post-hoc test was used to assess statistically significant differences between the means of respective tissues, which were shown in different letters (P<0.05).

(A-I) *EF-1A*

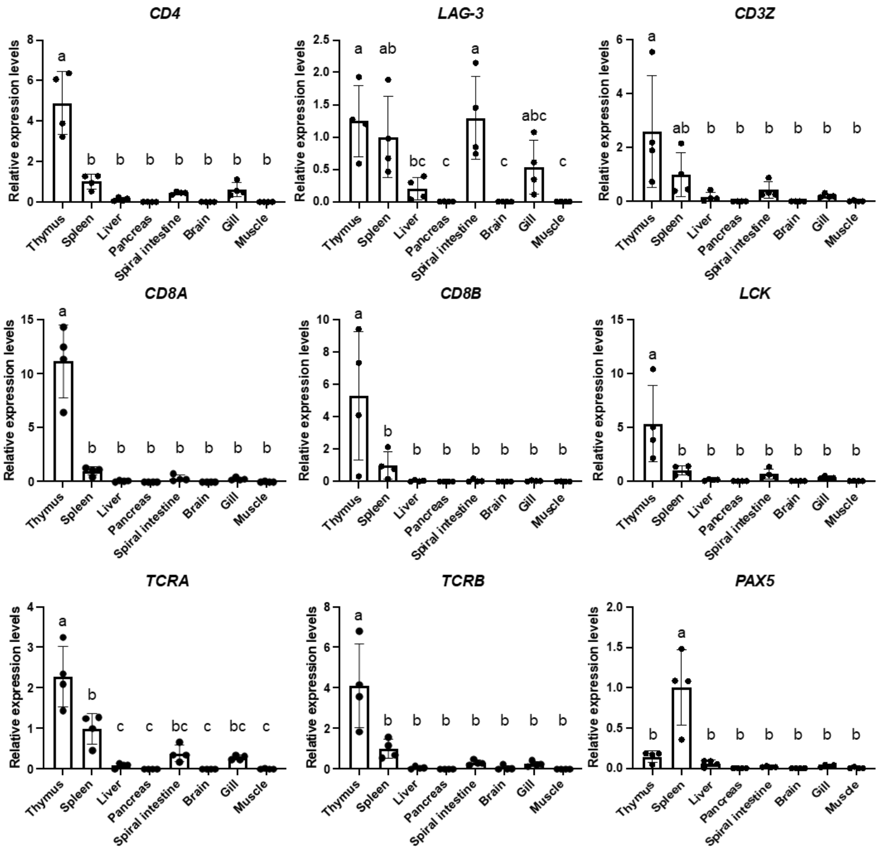

(A-II) *RPL13*

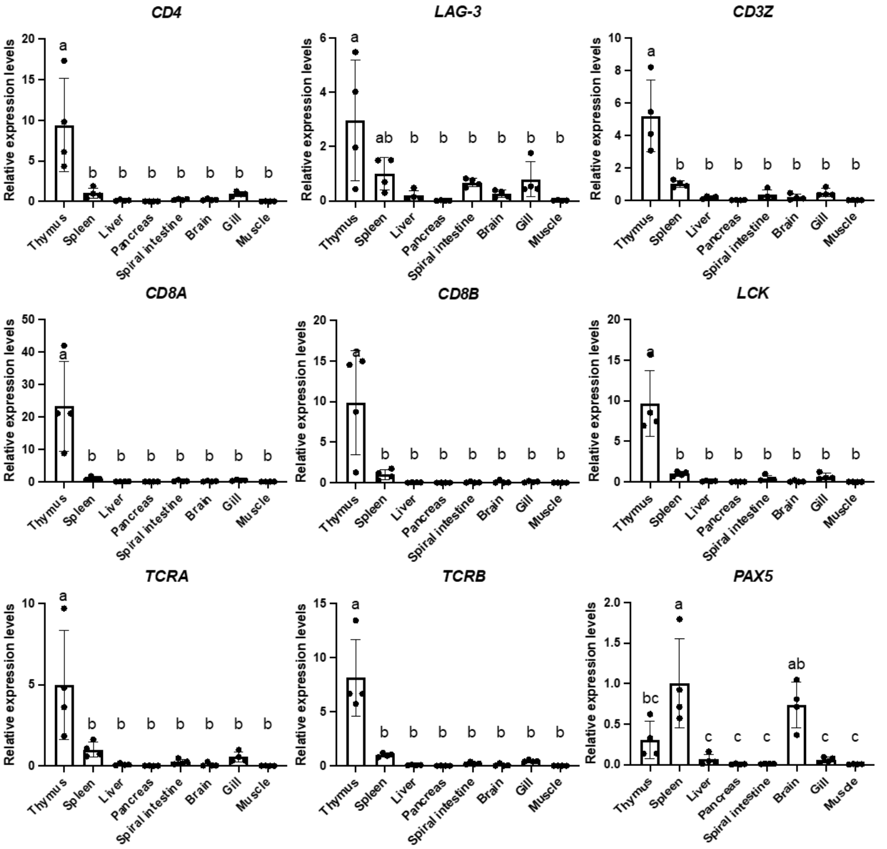

(A-III) *ACTB*

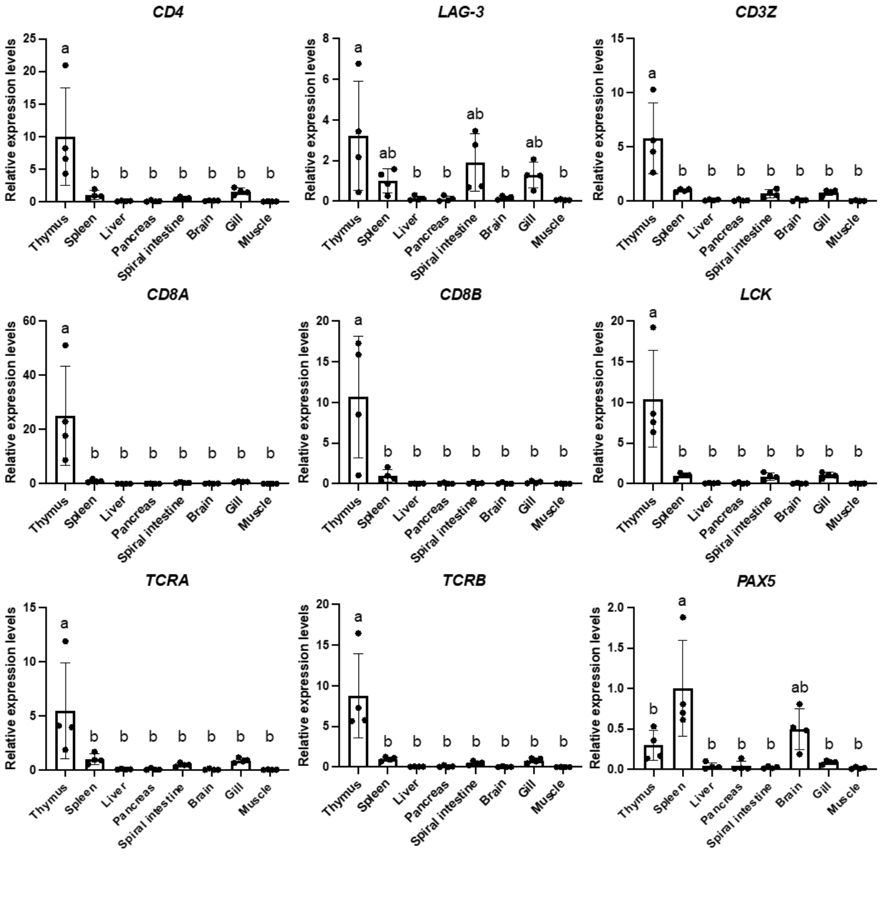

(A-IV) Total RNA

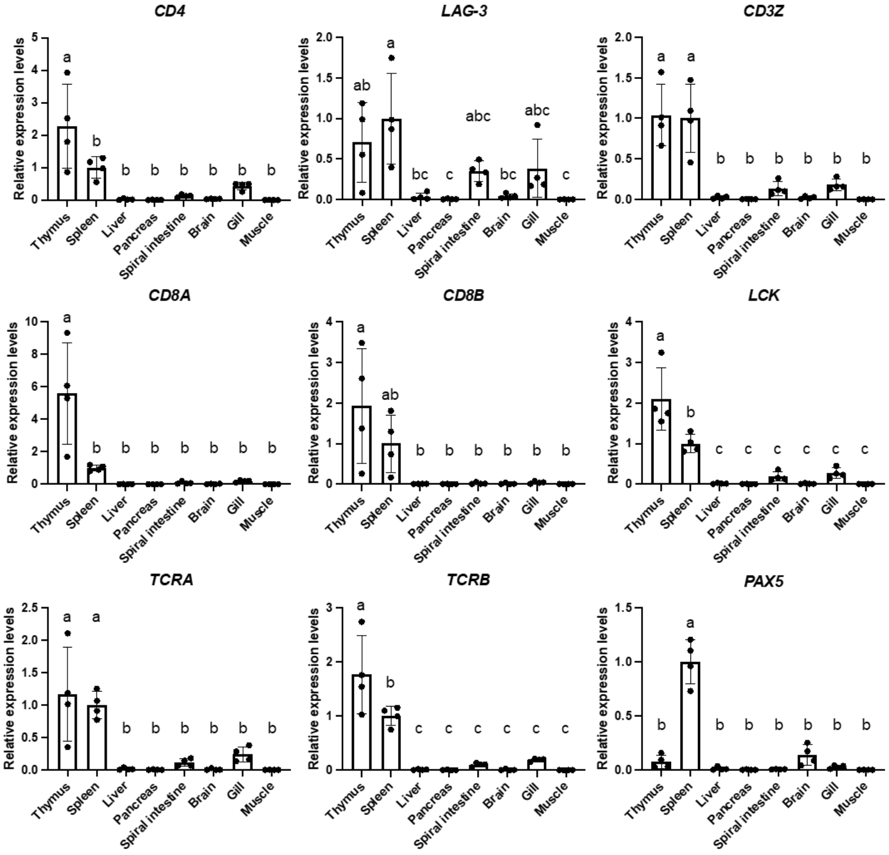

(B) Northern blot analysis for *CD4*, *CD8A*, and *CD8B* in nurse shark (*Ginglymostoma cirratum*)

Methods: A nurse shark individual was captured off the Florida Keys by a licensed collector. The shark was placed and left in a 250 - 500mg/ L buffered MS-222 solution for a minimum of 30 minutes after cessation of opercular movement (anesthesia to respiratory arrest) prior to removal from the anesthetic and pithing to assure euthanasia. All animal procedures were approved by the University of Maryland Baltimore Animal Welfare Department (IACUC). Total RNA was extracted from different tissues using Trizol (Invitrogen) following the manufacturer's protocol. Twenty micrograms of total RNAs, extracted from various tissues of nurse shark were electrophoresed and blotted onto Optitran Nitrocellulose membrane (Schleicher & Schuell). The membrane was hybridized in 50% formamide solution with the following nurse shark probes and washed under high-stringency conditions (Bartl et al., 1997). The probe sequences were entirely or for their largest parts derived from coding sequences (for gene sources see Supplementary file 2):

CD4 probe,

```
CCTGTCGGTACAAACATGTCCCTCGTCTGTAACGTCTCGAGCCACAGCGGCCAGACAGGGATACGGTGGAGGA
GCCCGAGCACGATGGAGGGATTGGAAGACAGGAGGGTCCGAGGAGAGGGATCTCTCCTGATCCGCCTCATCGA
GGTGACCCAGCGCCACGTGGGTGACTGGATCTGCGAGATCAGCCAGGGCGATCAGCTGTGCGGGCAAGGGACC
TACTCCCTCAACATCACAACCCTGACCTTGAGCGAGTTCGGGGACCCGCCCTTGGTCCTGATTATCGCAGCCT
CTGTCGGGGCATTTGTTCTCCTCCTGTTGGCCACCGTGATCGCCGTCTGCCTTTCCAAGCGAGCTCGGAGACG
GAGACGGGCCCTGAAGAGACTGAGACATCCGCTGTGTCGGGAACACAGTTACCAGCTCTCGAACCAGCCCCTG
TGTCACAGCAACGAC;
```

CD8B probe,

```
GAATCAATCCTACAGCAGACTCCGAGAGAAATAGTGACGACCAAAGGCGGACAGATCAAACTCTCCTGTAATC
TGAAGAAGGGCTCACTCGAAGAGATTTTCTGGTACAAACTAGACGGAAACTCCGAGGCCCATTTTCTGAAGTC
TGCCAGTATCCTGAACAAACAGACCTCAGGAGAGGGAATAACCGAACGGTTCGTTTTAACCAAAGATACCTTT
CGACTGTCGTTCAGTTTAAGCATCCAAAACACGCTGCTGTCAGACAACGGCACCTACTACTGCCTGATGATCA
AATCCTACTCCATGTACATGGGAAGTGGAACTGCGGTCATAGTAGTTCCAGAACAAGAGAAAGTAACTTTGCC
GCCTCCAACGACTAAAAGCGGCGGAATCAATCGCGTTACCCAGCCCAAGGTACGACCGAAGAAGAAAGCGGGG
AGTCACGGGTACGCGTGTAACTGGAGTATCTGGGTCTCTCTTGCCGTTTGCAACCTCATGCTCCTGACCTCCG
TCATCTTTGTTGTTATCAAACACAAGATCCAGAGTAAGGG;
```

CD8A probe,

```
CACTGGATGCAGACGAGGGAGTTTATTGGTTCCAACAGCCCAGGAATTCCGGCCCGAAGTTCTTGTTGTATGT
AACAGGCACAGGTAAACCGAAAAGTGCGAGCAACCCCGAAAGACACACTGCAGGGAAATCAGCCAAAAAAGTG
ACTCTGACGATCAAGGAGTCTGTGGAAGAAGACGAGGGGAAGTATTACTGTTTCATGGTCAAGAACATGGTCA
TGATGTTTGGGGACATCACTGACCTGGACATTGAAGGGGTCGCAACCACGCCAGAGCCAACGACCATTCCCAC
CACGACACAAAAAATACCAATTACTACGGACAGCAAAGGATCTACTCAATGTCATTCAACTAAAAGAGAGAAA
ACAGAAGATCCATTGAGCTGCCACTTTATCTTCTGGGCTCCTCTGACTGGCGCTGCCGCTCTGTTGCTCATCG
CACTGACCAGTGTCTCCATCGCTTATTGCAGAAGACCCCGCCGGAGACGCTGTCAGCACCAATTTCGAAAGAG
```

ACCAATAGCTGAAGAAGATAGACTATCAAACAGATATCTTTAACAAGTTCATTCTGGAAAACGTGTGCCATTC ATCCATGG.

Results: Among different nurse shark tissues, the highest expression of *CD4*, *CD8A*, and *CD8B* was found in the thymus. These blots were made a long time ago, and at the time shark *LAG-3* had not been found yet.

Reference: Bartl S, Baish MA, Flajnik MF, Ohta Y. Identification of class I genes in cartilaginous fish, the most ancient group of vertebrates displaying an adaptive immune response. J Immunol 1997 Dec 15;159(12):6097-104

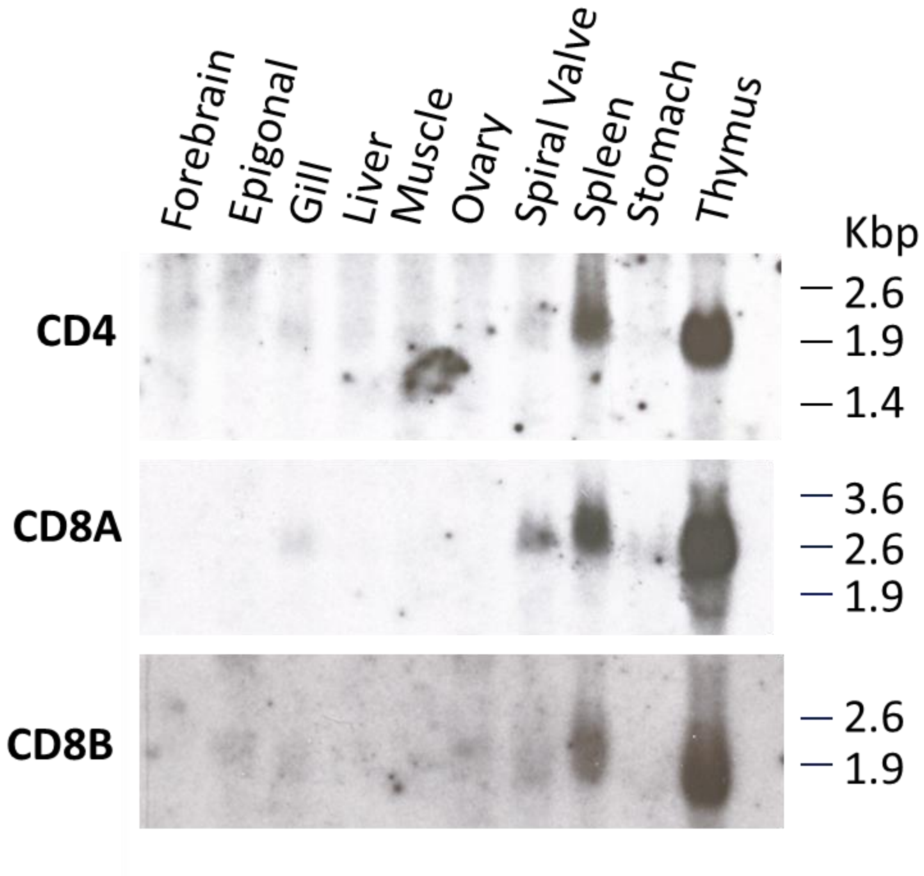

(C) Single nuclei RNA sequencing (snRNA-seq) data analysis of the expression of *CD4*, *LAG-3*, and other immune genes in splenocytes of nurse shark (*Ginglymostoma cirratum*)

Methods:

For single nuclei RNA sequencing (snRNA-seq) analysis of nurse shark splenocytes, we used the data generated by Matz et al., 2023, and deposited as GenBank accession GSE232302.

Matz and co-workers had immunized two animals subcutaneously in the ventral side of the pectoral fin with 300 µg R-phycoerythrin (PE) emulsified in Freund's complete adjuvant, and isolated spleens from two animals, sacrificed at days 40 and 50, for creating snRNA-seq libraries s1 and s2, which we combined for our analysis.

Raw FASTQ files from GSE232302 and reference files were processed according to the previous report (Matz et al. 2023). In brief, reference genome and gene annotation files of whale shark (*Rhincodon typus*), the genetically closest shark species available for published reference files, were obtained from GenBank accession GCF_021869965. Specific nurse shark genes shown in Supplementary file 2 were inserted to whale shark reference files. Raw FASTQ files were processed using StarSolo (version: 2.7.9a) (Dobin et al. 2013) to align reads with following optimized score: –soloCellFilter TopCells 25000, –soloUMIdedup 1MM_Directional, –outMultimapper Order Random, –outFilterScoreMinOverLread 0.15, –outFilterMatchNminOverLread 0.15, –soloFeatures GeneFull. After generating read count data from each nucleus, we conducted quality control for each sample. Specifically, we excluded nuclei containing fewer than 800 UMIs or 500 genes. Additionally, we identified and removed potential doublets from each sample using DoubletFinder (Version: 2.0.3) (McGinnis et al. 2013). After unifying each treated sample into a single file, snRNA-seq data was visualized by Seurat package (Version: 4.9.9.9059) (Hao et al. 2021). First, variable genes were identified as capable makers for classifying nurse shark splenocyte subtypes by applying a variance stabilizing transformation (VST) to a normalized matrix. Next, we conducted principal component analysis (PCA) and created a plot graph using shared nearest neighbor (SNN) clustering of the subtypes. Subsequently, this information was transformed into a Uniform Manifold Approximation and Projection (UMAP) for dimension reduction and intuitive visualization. Nurse shark CD3Z and TCRA enriched clusters were determined as T cell clusters. The gene expression of nurse shark splenocytes was visualized using Feature Plot, Violon Plot, and Dot Plot.

Results:

The results are summarized in the main text and here described in the figure legends.

References

Dobin A, Davis CA, Schlesinger F, Drenkow J, Zaleski C, Jha S, Batut P, Chaisson M, Gingeras TR. STAR: ultrafast universal RNA-seq aligner. Bioinformatics 2013 Jan 1;29(1):15-21.

Hao Y, et al. Integrated analysis of multimodal single-cell data. Cell 2021 Jun 24;184(13):3573-3587.

Matz H, Taylor RS, Redmond AK, Hill TM, Ruiz Daniels R, Beltran M, Henderson NC, Macqueen DJ, Dooley H. Organized B cell sites in cartilaginous fishes reveal the evolutionary foundation of germinal centers. Cell Rep 2023 Jul 25;42(7):112664.

McGinnis CS, Murrow LM, Gartner ZJ. DoubletFinder: Doublet Detection in Single-Cell RNA Sequencing Data Using Artificial Nearest Neighbors. Cell Syst 2019 Apr 24;8(4):329-337

(C-I)

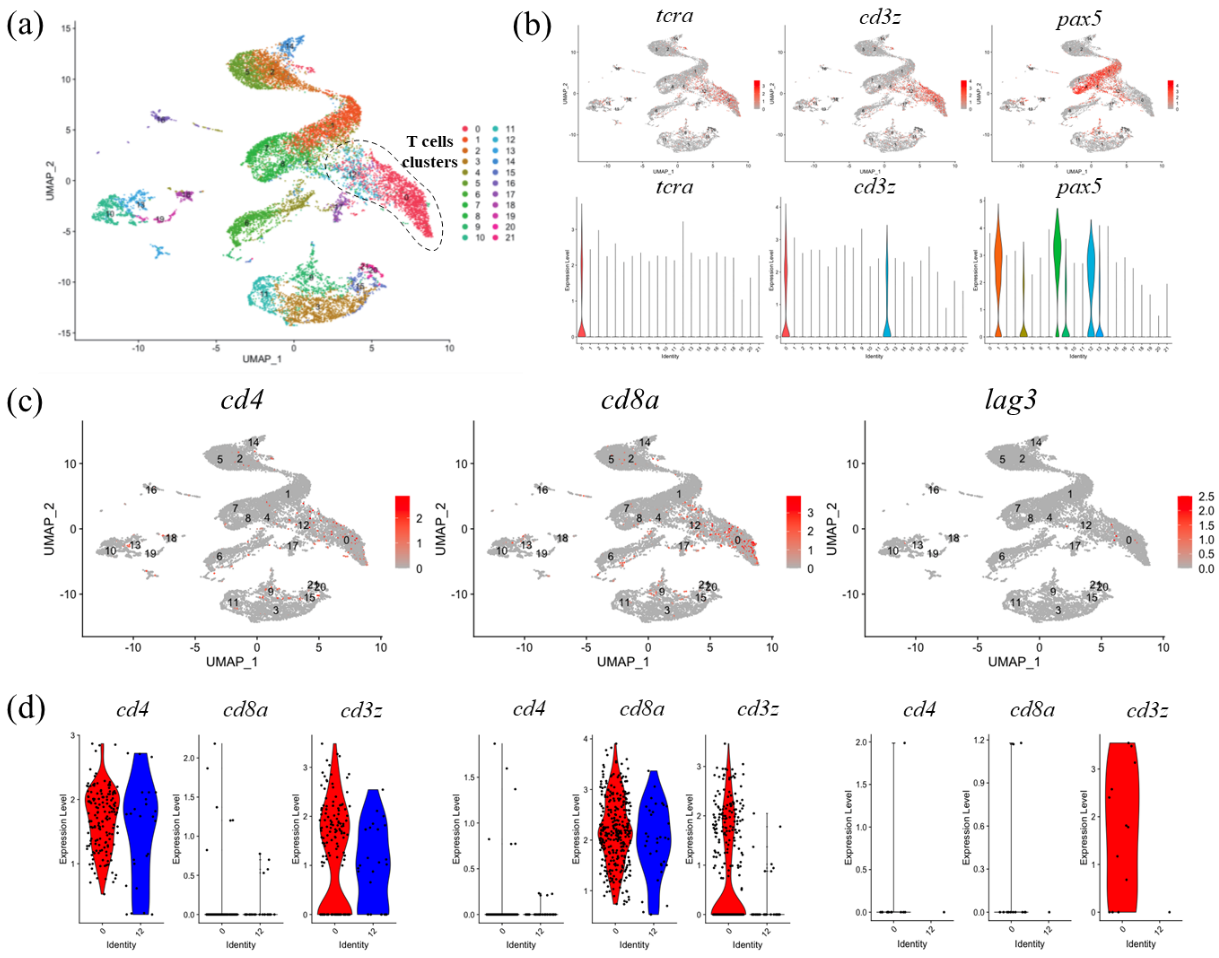

(C-I) (a) snRNA-seq samples from splenocytes have been divided into 22 clusters and are displayed on UMAP plot. The major T cells clusters (positive for *TCRA* and *CD3Z*) are enclosed by a dashed line. **(b)** Gene expression of T cells marker genes (*TCRA* and *CD3Z*) and B cells marker genes (*PAX5*) in each cluster are shown on Feature Plots (upper) and Violin Plots (bottom), respectively. **(c)** Gene expression of *CD4*, *CD8A* and *LAG-3* in whole splenocytes is shown on Feature Plots, respectively. **(d)** Gene expression of *CD4*, *CD8A*, and *CD3Z* in *CD4* positive cells (left), in *CD8A* positive cells (middle) and in *LAG-3* positive cells (right) among T cells clusters (cluster 0 and 12) is shown on Violin Plots, respectively.

(C-II)

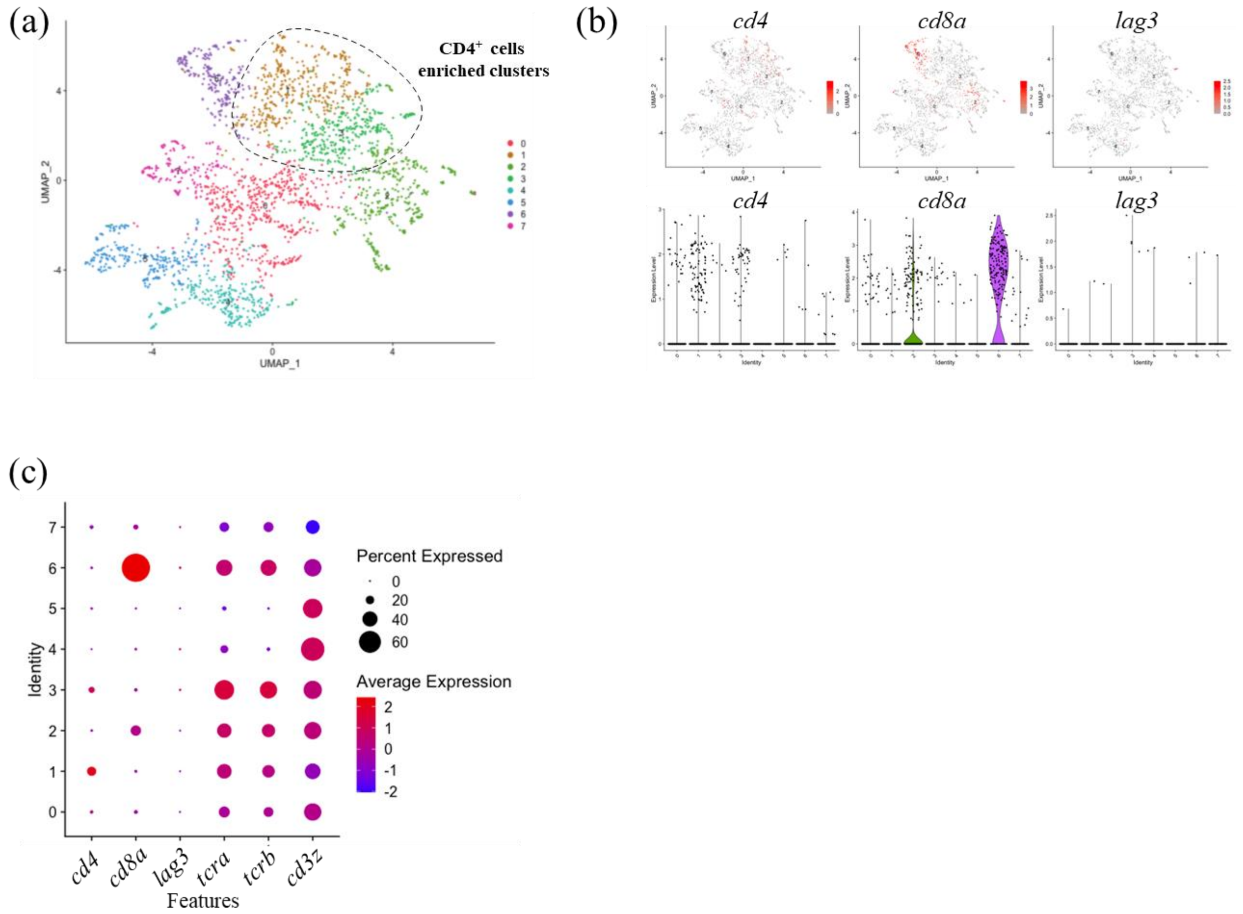

(C-II) (a) T cells clusters (cluster 0 and 12 of whole splenocytes) are further subdivided into 7 clusters and are displayed on UMAP plot. The clusters enriched with *CD4* positive cells are enclosed by a dashed line. **(b)** Gene expression of *CD4*, *CD8A*, and *LAG-3* in *CD4* positive cells (left), in *CD8A* positive cells (middle), and in *LAG-3* positive cells (right) among T cells subclusters is shown on Violin Plots, respectively. **(c)** Gene expression of T cell marker genes *CD4*, *CD8A*, *LAG-3*, *TCRA*, *TCRB*, and *CD3Z* in T cells subclusters is depicted by Dot Plot.
