# Supplementary material for: CD4 and LAG-3 from sharks to humans: related molecules with motifs for opposing functions
Source: Front Immunol. 2023 Dec 21;14:1267743. doi: 10.3389/fimmu.2023.1267743 (PMC10768021; doi:10.3389/fimmu.2023.1267743)
Supplement: Supplementary file 10 [file DataSheet_10.pdf]

Supplementary file 10

A *CD4/LAG-3* hybrid type of gene in lungfishes

As shown in the main text Fig. 1f (repeated here as Fig. 1), in West African lungfish (*Protopterus annectens*), between the *CD4* and *LAG-3* genes a *CD4/LAG-3* hybrid gene appears to be located.

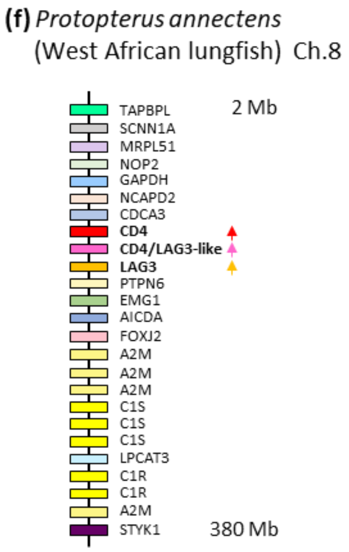

Figure 1. The genomic region surrounding the *CD4* and *LAG-3* genes in *Protopterus annectens* (West African lungfish), according to the PAN1.0. genomic assembly (GenBank GCA_019279795.1). This figure was shown already as Fig. 1f in the main text, and is shown here again for convenience.

In the genomic assembly PAN1.0. at GenBank, these *CD4/LAG-3* family genes are predicted as follows: *CD4* (LOC122808646, encoding XP_043935549), the CD4/LAG-3 hybdrid (LOC122808648, encoding XP_043935552), and *LAG-3* (LOC122808649, encoding XP_043935555) (Fig. 2).

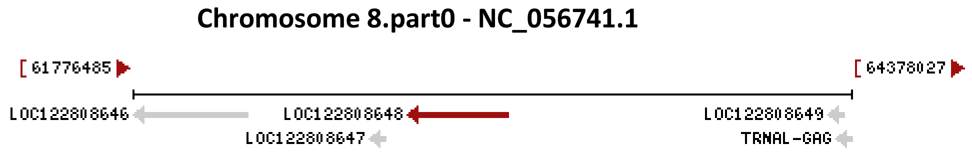

Figure 2. Gene locus annotations in West African lungfish Chr. 8 (dataset PAN1.0) as by GenBank. LOC122808646 is *CD4*, LOC122808648 is a *CD4/LAG-3* hybrid, and LOC122808649 is *LAG-3*. LOC122808647 is not a coding sequence. The figure is similar to Fig. 1 but is shown here to explain the predictions by GenBank.

For the beginning and end of the predicted West African lungfish CD4/LAG-3 hybrid sequence (XP_043935552), non-overlapping TSA transcript accessions are available as GenBank accessions GGXP01068868 and GGXP01068867 (Fig. 3).

The predicted hybrid sequence XP_043935552, however, seems to have an incorrect N-terminus as it appears to start within a CD4 Ig-like D1 sequence with internal phase 1 intron. Comparisons with the genomic sequence and the TSA transcript sequence GGXP01068868 available at GenBank find the N-terminal part of that D1 domain (Fig. 3). We have not been unable to find a leader sequence for the West African lungfish CD4/LAG-3 hybrid yet, but a predicted leader sequence is found in the CD4/LAG-3 hybrid homologue in South American lungfish (*Lepidosiren paradoxa*) encoded by GenBank GEHZ01078121 (a TSA sequence) (Fig. 4). For South American lungfish, also canonical CD4 (GenBank GEHZ01044481, a TSA sequence) and canonical LAG-3 (GenBank GEHZ01011572, also a TSA sequence) were found, suggesting a similar situation in different lungfishes. However, for South American lungfish, there is no genomic information available yet for these genes. The cytoplasmic tail of these hybrid sequences clearly is a LAG-3 type of tail (see the blue shading in Figs. 3 and 4), while upon comparison of the predicted ectodomains with the GenBank database they have CD4 sequence of West African lungfish (GenBank XP_043935549) as top-match. However, the amino acid identity with that sequence is only about 30%, suggesting that the CD4 part of the CD4/LAG-3 hybrid origin included an old *CD4* gene duplication early in the lungfish lineage. The LAG-3-typical tails in these hybrid molecules also do not look like a recent duplication from the canonical lungfish LAG-3 and may also be very old. These lungfish CD4/LAG-3 hybrid molecules are a bit peculiar, and we would need more information for allowing a thorough discussion.

LEILCNVVVSGTAGGSVLLPCMGPAGLKLFKWTDDKGAIIVQIMRSGMIFYG
(1)STIKKDRFKSADYSSGNYSLLMSQLEPHDSGQYTCSSSEADLNKVNLHV
YLA(1)EPSPPEIHTPATERGNTVLPCSAELHNYVSFPWTFMSTSRATIEVR
NGWILPG(1)IYAGGEPEVIFGIVNQSSVLNFTFTNDTRPCPKTTYADLLSG
KITFSHVKGGAQVELAFMNVTQSPLLWKETEGSHSTEFRFSLVVCPTYYMDA
GWYTLTLNFTKGVLQKELRLITMKV(1)THYWSKVTTGTHVNLTCQVSNVLP
FMSLTWFHIINSTQYNKVLDQTGVAELTVTFPNVTADPVGLWRCCLYVKTQK
KVCADYNLE(1)KTNHLSTGISVSLQKILLGGGTVTAGILCLIIIIFCIRRC
RQ(0)SNETKFPYLENALKARRAPADYD

Figure 3. The West African lungfish CD4-LAG-3 hybrid sequence as predicted by GenBank (XP_043935552) plus an N-terminal extension (underlined) for completion of the Ig-like domain sequence as supported by the TSA sequence GGXP01068868 and the PAN1.0. genomic sequence. We have not been able to find a leader sequence for this sequence yet. The TSA sequence GGXP01068868 provides transcript support until its end for the N-terminal part of the depicted sequence until the yellow-shaded arginine, while the TSA sequence GGXP01068867 provides transcript support from its beginning for the C-terminal part of the depicted sequence starting from the magenta-shaded leucine. The numbers between brackets refer to introns and their phases at the indicated position (0) or in the preceding codon (1,2), based on comparison with the PAN1.0. genomic sequence. The cytoplasmic tail is a typical LAG-3 tail, considering the number of exons and the FxxLE motif.

MQKREAYLILHALCLLNTGWGILCEVVVPGTAGGSALLPCVGPLSLKPAFKW
TDPKGVKIVQIMPSGMRFYAPRIKKDRLKFADYSSGNYSLMISQLELHDSGL
YICTSSERNLNKVNLQVYPVPEPSPPPEIHVLATENGNTVLPCSGHPQNSTS
FSWTFISTSRTVIAIRNGQISPGGTADRLHFQLADYMNGNCSLLLVNVMQRD
AGLYQCSCNLQKVRLYVIQVSCQNNERSHPLHSTVLMCAVIPEFHGLTRKWI
HPDGSQVFNTSHYRLYQNSKALELKHLNRNDNGHWVCRISVGQQSLDASIDI
NIIGIYASEQPGTVFSIVNRSAVLHFTFTDDTRPCPKNTDAELLSGKVTFSR
VKGGTHVALAFLNTTQSPLLWNETGDRMITVLRNHSLGLNFSLVVHNTYYTD
AGLYTLTLNFTKGVLQKELQLITMQVTHNPWGKVTAGSQVNLTCQVSDLLPF
MRLTWFHINNTTYTQAVQESDELTVTIPNVTAECFGLWRCCLYVEMQVKTCA
DYNLEQADYHSSGISLSLQKILLGGGTLTVLFLSTILMLIIRRCRQQNEEKF
EALENALKTRLSPADYE

Figure 4. The CD4/LAG-3 hybrid sequence in South American lungfish (*Lepidosiren paradoxa*) as encoded by GenBank accession GEHZ01078121 (a TSA transcript sequence). This sequence does have a predicted leader sequence (shaded gray) and also carries the LAG-3 cytoplasmic tail motif FxxLE.
